# Comparison of submucosal and subserosal approaches toward optimized indocyanine green tracer-guided laparoscopic lymphadenectomy for patients with gastric cancer (FUGES-019): a randomized controlled trial

**DOI:** 10.1186/s12916-021-02125-y

**Published:** 2021-10-27

**Authors:** Qi-Yue Chen, Qing Zhong, Ping Li, Jian-Wei Xie, Zhi-Yu Liu, Xiao-Bo Huang, Guang-Tan Lin, Jia-Bin Wang, Jian-Xian Lin, Jun Lu, Long-Long Cao, Mi Lin, Qiao-Ling Zheng, Ru-Hong Tu, Ze-Ning Huang, Chao-Hui Zheng, Chang-Ming Huang

**Affiliations:** 1 Department of Gastric Surgery, Fujian Medical University Union Hospital, No. 29 Xinquan Rd, Fuzhou, 350001 China; 2 Department of General Surgery, Fujian Medical University Union Hospital, Fuzhou, China; 3 Key Laboratory of Gastrointestinal Cancer (Fujian Medical University), Ministry of Education, Fuzhou, China; 4 Fujian Key Laboratory of Tumor Microbiology, Department of Medical Microbiology, Fujian Medical University, Fuzhou, China; 5 Department of Pathology, Fujian Medical University Union Hospital, Fuzhou, China

**Keywords:** Gastric cancer, Indocyanine green, Submucosal approach, Subserosal approach, Lymphadenectomy

## Abstract

**Background:**

Application of indocyanine green (ICG) fluorescence imaging is effective in guiding laparoscopic radical lymphadenectomy for gastric cancer. However, the optimal approach for indocyanine green injection is controversial. Therefore, the objective of this study was aimed to compare the efficacy and ICG injection between the preoperative submucosal and intraoperative subserosal approaches for lymph node (LN) tracing during laparoscopic gastrectomy.

**Method:**

This randomized controlled trial (ClinicalTrials.gov, NCT04219332) included 266 patients with potentially resectable gastric cancer (cT1–T4a, N0/+, M0) enrolled from a tertiary teaching center between December 2019 and October 2020. The primary endpoint was total number of retrieved LNs.

**Results:**

In total, 259 patients (*n* = 130 and *n* = 129 in the submucosal and subserosal groups, respectively) were included in the per-protocol analysis. There are no significant differences in total number of retrieved LNs between the two groups (49.8 vs. 49.2, *P* = 0.713). The rate of LN noncompliance in the submucosal group was comparable to that in the subserosal group (32.3% vs. 33.3%, *P* = 0.860). No significant difference was found between the submucosal and subserosal groups in terms of the incidence (17.7% vs. 16.3%; *P* = 0.762) or severity of postoperative complications. The mean fluorescence cost in the submucosal group was higher than that in the subserosal group ($335.3 vs. $182.4; *P* < 0.001). The overall treatment satisfaction score was lower in the submucosal group than in the subserosal group (70.5 vs. 76.1%, *P* = 0.048).

**Conclusion:**

ICG administered by subserosal injection was comparable to that administered by submucosal injection for lymph node tracing in gastric cancer. However, the former approach imposed a lower economic and mental burden on patients undergoing laparoscopic D2 lymphadenectomy.

**Trial registration:**

ClinicalTrials.gov, NCT04219332.

**Supplementary Information:**

The online version contains supplementary material available at 10.1186/s12916-021-02125-y.

## Background

Curative treatment of gastric cancer (GC) depends on operation-centered comprehensive treatment. Effectively achieving systematic lymphadenectomy without increasing surgical complications is the goal of surgeons. Indocyanine green (ICG) fluorescence imaging-guided lymphadenectomy, a recently developed technique with upgraded minimally invasive visual display systems, is believed that could be used to achieve this goal [1].

The key to effective intraoperative lymph node (LN) visualization depends on ICG injection. The existing injection methods include the submucosal approach (SMA) and subserosal approach (SSA). The results of previous retrospective studies [2, 3] and randomized controlled trial (RCT) [4] showed that submucosal injection of ICG around tumors 1 day before surgery could achieve good tracing of perigastric LNs, thus significantly increasing the overall number of retrieved LNs without increasing surgery-related complications in patients undergoing laparoscopic surgery.

Traditional preoperative submucosal injections seem to be the preferred method. However, preoperative submucosal ICG injection is generally performed 1 day before surgery when patients have extremely high physical and mental burden [5]. This method may increases patient discomfort and the endoscopist’s workload while performing tracer injection in cases of unresectable GC, such as GC with unpredictable peritoneal metastases, which is prone to medical waste. Moreover, according to the refined modern medical division of labor, in many centers, intraoperative submucosal injection usually requires an extra endoscopic team in addition to the surgeon, which dramatically reduces the convenience and coordination during surgery, which limits the application this technique. Herrera-Almario et al. [6] found that subserosal injection of ICG helps surgeons visualize LNs effectively in robotic gastrectomy, thus improving the quality of lymphadenectomy. A retrospective study by Baiocchi et al. [7] suggested that ICG tracer-guided LN dissection can be achieved either by submucosal or subserosal injection. Compared with submucosal injection 1 day before surgery, intraoperative subserosal injection before lymphadenectomy is theoretically more convenient for surgeons and can reduce the workload of endoscopists; however, it is associated with a possible risk of poor imaging.

Currently, the optimal ICG injection method for laparoscopic fluorescence imaging-guided lymphadenectomy in radical GC surgery, considering the effectiveness of LN tracing, economic benefits, and patient burden, is controversial. Hence, the Fujian Medical University Union Hospital Gastric Surgery Study (FUGES) Group conducted a RCT (FUGES-019) to compare the efficacy, safety, and cost-effectiveness of the SMA and SSA for ICG injection for LN tracing during laparoscopic gastrectomy in patients with GC.

## Methods

### Study design

A phase 3, parallel, open-label RCT (ClinicalTrials.gov, NCT04219332) conducted at Fujian Medical University Union Hospital. The primary endpoint was the total number of retrieved LNs. The secondary endpoints were the total number of fluorescent LNs, postoperative recovery course, morbidity and mortality rates, and 3-year disease-free survival rate. The trial protocol (Additional file 1) was approved by the Institutional Review Board (IRB number 2019YF045-01). All authors had access to the study data and reviewed and approved the final manuscript.

### Participants

The inclusion criteria were as follows: (1) age 18–75 years, (2) primary gastric adenocarcinoma, and (3) a tumor stage of cT1–cT4a, N0/+, M0 at preoperative evaluation. The exclusion criteria were as follows: (1) a history of previous upper abdominal surgery, gastrectomy, endoscopic dissection, and (2) linitis plastica. The detailed inclusion and exclusion criteria are provided in Additional file 2: Table S1. All participants provided written informed consent.

### Randomization and blinding

Eligible patients were randomly assigned in a 1:1 ratio to receive either submucosal or subserosal injection of ICG. The data manager (F.-F.L.), who was not involved in eligibility assessment and recruitment of patients, performed the randomization with a list of randomly ordered treatment identifiers generated by a permuted block design using SAS (version 9.4; SAS Institute Inc.). The allocation sequence was concealed from the surgeons who enrolled patients until they were formally randomized to their groups. Although it was not feasible to blind the surgeons and participants, the pathologists were unaware of the intervention received by the patients. The researcher performing the statistical analyses was blinded to the patient group allocation.

### Interventions

ICG was endoscopically injected around the tumor in patients in the SMA group 1 day before surgery (Additional file 5: Video); 1.25 mg/mL ICG was prepared in sterile water, and 0.5 mL of the solution was injected into the submucosal layer at four quadrants around the primary tumor, amounting to 2.5 mg of ICG. Patients in the SSA group underwent laparoscopic subserosal injection of ICG 20 minutes before lymphadenectomy. Additional file 5: Video shows the preoperative preparation before subserosal injection. Based on the characteristics of perigastric lymph drainage, we created a set of injection procedures according to the proposed surgical resection method, named Huang’s subserosal hexa-points maneuver (Additional file 5: Video). ICG powder is dissolved in 0.5 mg/mL of sterile water, and the prepared solution (1.5 mL for each point) is injected along the subserosa of the stomach at six specific points along the lesser and greater curvature of the stomach (Additional file 3: Fig. S1). If the tumor invades one or more of the six injection points, it is specified in this study protocol that subserosal injection of ICG will be conducted at the tumor non-invasive sites along the greater or lesser curvature of the stomach next to the established injection point. The NOVADAQ fluorescence surgical system (Stryker Corp., Kalamazoo, MI, USA) was used to obtain near-infrared (NIR) fluorescent images. Intraoperatively, the fluorescent mode could be switched according to the situation (Additional file 5: Video).

### Surgical quality control

All surgeries were performed by the same surgical team, and another group of surgeons weekly reviewed the unedited surgery videos using a quality control checklist (Additional file 2: Table S2). D2 lymphadenectomy was performed according to the Japanese GC Treatment Guidelines 2018 [8]. Standard resection methods were routinely performed as previously described [9]. After lymphadenectomy, NIR imaging was routinely performed for final observation of residual fluorescent LNs, and any remaining stained nodes were removed (Additional file 5: Video).

### Outcome measurements

**Fig. 1:**
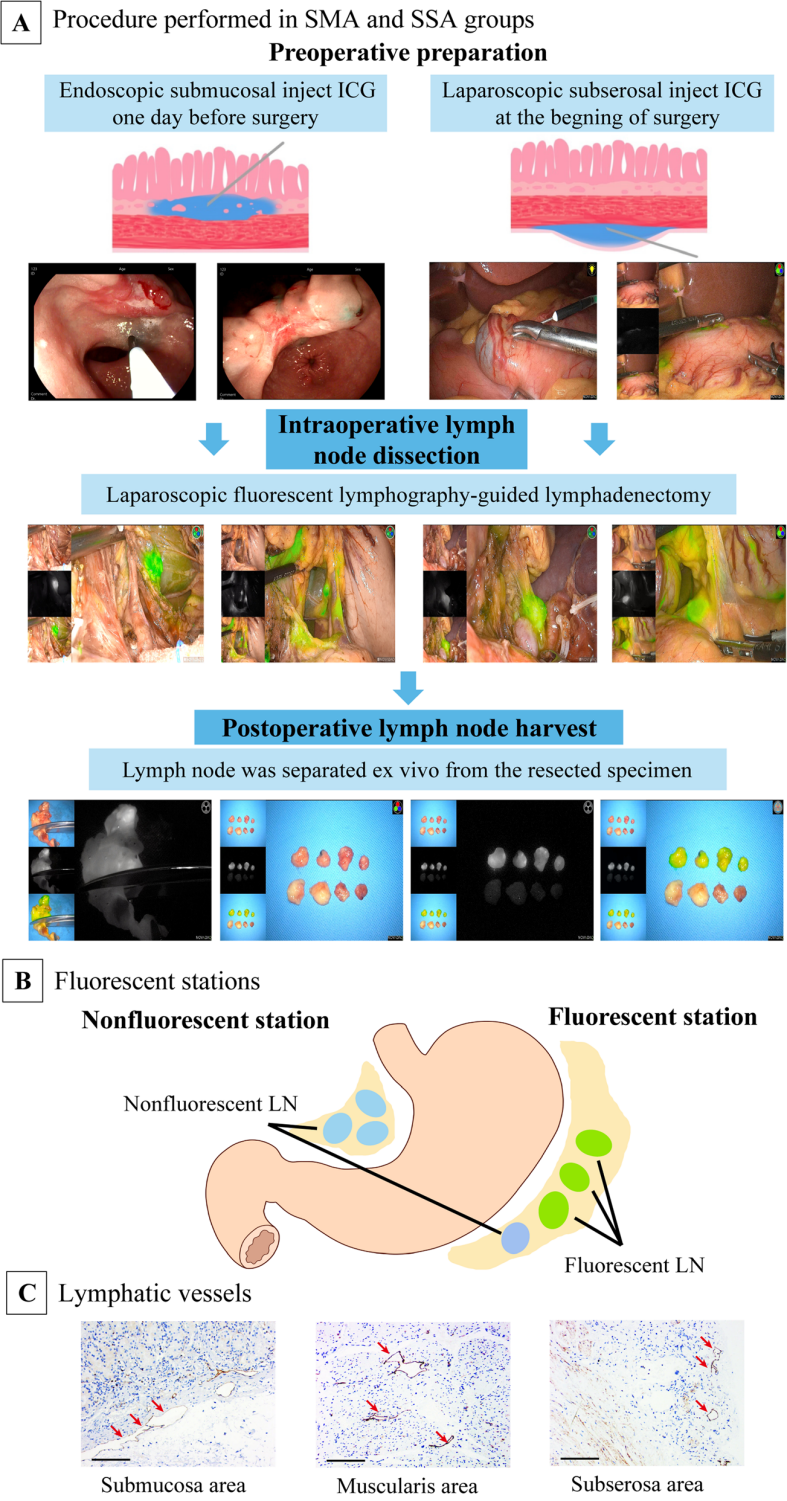
Procedures performed in the SMA and SSA groups, illustration of fluorescent lymph nodes and stations, and lymphatic vessels stained with D2-40 (podoplanin). ICG, indocyanine green; LN, lymph node. The red arrow represents lymphatic vessels. Scale bar, 200 μm

LN-bearing soft tissues were separated from the resected specimens in vitro according to the Japanese classification guidelines [10]. Fluorescent LNs were retrieved from each station directly through NIR imaging (Additional file 5: Video). LNs emitting fluorescence were considered fluorescent LNs. Stations containing fluorescent LNs were classified as fluorescent stations (Fig. 1). Surgeons examined all specimens, which were immediately sent to the pathology department after surgery. All pathological examinations were performed in a standard manner [10].

The LN dissection rate was determined by the number of patients in whom a LN station was harvested divided by the total number of patients who required retrieval at the corresponding LN station. Within the scope of D2 dissection, LN noncompliance was defined as the absence of LNs that should have been resected from > 1 LN station. Major LN noncompliance was defined as > 2 intended LN stations not removed [11]. The American Joint Committee on Cancer suggests that at least 16 regional LNs should be removed pathologically, and the removal of ≥ 30 LNs is desirable [12].

Morbidity and mortality within 30 days after surgery were assessed. Postoperative complications were graded according to the Clavien-Dindo classification [13]. Patient satisfaction with care was measured before discharge from the hospital using the modified European Organisation for Research and Treatment of Cancer (EORTC) IN-PATSAT14 scale (Additional file 2: Table S3). The modified EORTC IN-PATSAT14 contains five multi-item and three single-item scales [14], which were linearly transformed to a 0–100 scale. A higher score reflects a higher level of satisfaction.

The total cost during hospitalization was calculated as the sum of indirect and direct costs [15, 16]. Indirect costs included the overhead cost of the amortization of capital equipment and supplies, maintenance, utilities, and administrative staff. Direct costs included the costs of all items and services during hospitalization, including equipment, laboratory tests, medications, and fluorescence-related costs. The fluorescence-related cost for the SMA included the cost of endoscopy, tracers, materials, and treatment, while that for the SSA included the cost of tracers, materials, and treatment.

The surgeons were routinely instructed to complete the Surgery Task Load Index (Surg-TLX) questionnaire (Additional file 3: Fig. S2) for each procedure [17]. It has six subscales addressing mental, physical, and temporal demands; task complexity; situation; and distractions. All questions were rated on a 20-point scale (0 = low, 20 = high).

We examined the distribution of lymphatic vessels by immunohistochemistry and immunofluorescence to explore the lymphatic drainage in the gastric wall (details in Additional file 4).

### Sample size and statistical analysis

Based on a previous RCT [4], the total number of retrieved LNs was 50.5 (15.9 SD) for patients who underwent ICG tracer-guided lymphadenectomy. A sample size of 111 patients per group was calculated for 80% power to detect a noninferiority margin of 6 with one-sided *α* = .025. Assuming an expected dropout rate of 20%, at least 133 patients were needed in each group. The sample size was calculated using nQuery Advisor 7.0 (Statistical Solutions Ltd.).

This study has been reported in line with the STROCSS criteria [18]. Analyses for all endpoints were performed in the per-protocol population. Continuous variables are presented as mean (SD), and categorical variables are presented as frequencies and percentages. The differences between the groups were assessed using the *t*-test, the Mann-Whitney test, Fisher’s exact test, or the *χ*^2^ test, as appropriate. All tests were two-sided with a significance level of *P* < .05. All data were analyzed using the SPSS statistical software (version 22.0; SPSS Inc.) and R software (version 3.6.1; R Foundation for Statistical Computing).

## Results

### Baseline characteristics

**Fig. 2:**
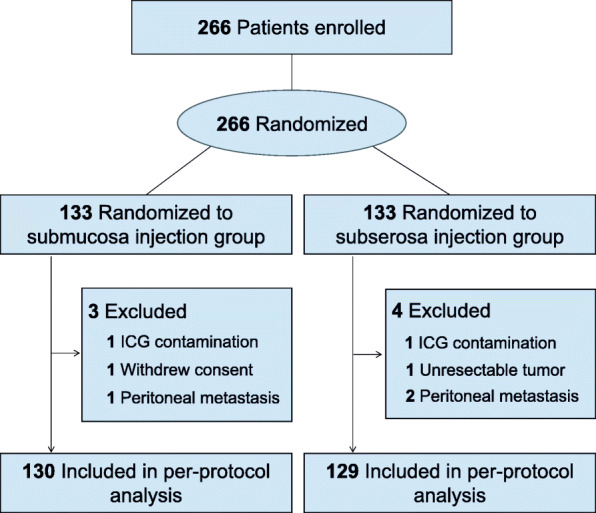
Study flowchart

From December 31, 2019, to October 27, 2020, 266 patients were randomized to either the SMA group or the SSA group. After surgery, three patients were excluded from the SMA group (one with ICG contamination due to leakage caused by mistakenly injecting ICG into the peritoneal space during endoscopy, one withdrew from the study, and one with peritoneal metastasis), and four patients were excluded from the SSA group (one with ICG contamination due to intraoperative leakage of ICG with the spoiling of the NIR view, one with an unresectable tumor, and two with peritoneal metastases). After exclusion, 130 patients in the SMA group and 129 patients in the SSA group were included in the per-protocol analysis (Fig. 2).

The baseline characteristics were well balanced between the groups (Table 1). The mean (SD) patient age was 58.8 (11.3) and 59.0 (10.3) years in the SMA and SSA groups, respectively. A stratified analysis by resection method indicated that the clinicopathological features were also balanced between the two groups (Additional file 2: Table S4).

**Table 1:** Basic characteristics of the SMA and SSA groups

Characteristic	Mean (SD)/No. (%)		***P*** value
	SMA (***n*** = 130)	SSA (***n*** = 129)	
**Age, years**	58.8 (11.3)	59.0 (10.3)	0.886
**BMI, kg/m**^**2**^	22.5 (3.2)	22.3 (3.1)	0.738
**Sex**			
Male	87 (66.9)	88 (68.2)	0.824
Female	43 (33.1)	41 (31.8)	
**ECOG performance status**			
0	107 (82.3)	107 (82.9)	0.892
1	23 (17.7)	22 (17.1)	
**Tumor location**			
Upper	27 (20.8)	21 (16.3)	0.527
Middle	31 (23.8)	37 (28.7)	
Lower	72 (55.4)	71 (55.0)	
**Surgical procedure**			
Distal gastrectomy	71 (54.6)	65 (50.4)	0.496
Total gastrectomy	59 (45.4)	64 (49.6)	
**Reconstruction**			
Billroth I	5 (3.8)	10 (7.8)	0.239
Billroth II	66 (50.8)	55 (42.6)	
Roux-en-Y	59 (45.4)	64 (49.6)	
**Histology**			
Differentiated	55 (42.3)	57 (44.2)	0.760
Undifferentiated	75 (57.7)	72 (55.8)	
**Lymphvascular invasion**			
Negative	74 (56.9)	80 (62.0)	0.404
Positive	56 (43.1)	49 (38.0)	
**Size, cm**			
≤ 4	75 (57.7)	83 (64.3)	0.273
> 4	55 (42.3)	46 (35.7)	
**cT category**			
cT1	41 (31.5)	46 (35.7)	0.871
cT2	19 (14.6)	16 (12.4)	
cT3	42 (32.3)	42 (32.6)	
cT4a	28 (21.5)	25 (19.4)	
**cN category**			
cN0	52 (40.0)	57 (44.2)	0.495
cN+	78 (60.0)	72 (55.8)	
**pT category**			
pT1	39 (30.0)	47 (36.4)	0.528
pT2	19 (14.6)	13 (10.1)	
pT3	48 (36.9)	43 (33.3)	
pT4a	24 (18.5)	26 (20.2)	
**pN category**			
pN0	52 (40.0)	58 (45.0)	0.942
pN1	27 (20.8)	24 (18.6)	
pN2	23 (17.7)	20 (15.5)	
pN3a	20 (15.4)	20 (15.5)	
pN3b	8 (6.2)	7 (5.4)	
**AJCC 8th pTNM staging**			
I	46 (35.4)	49 (38.0)	0.767
II	34 (26.2)	36 (27.9)	
III	50 (38.5)	44 (34.2)	
**CEA, ng/ml**			
< 5	110 (84.6)	104 (80.6)	0.396
≥ 5	20 (15.4)	25 (19.4)	
**CA19-9, U/ml**			
< 37	110 (84.6)	112 (86.8)	0.612
≥ 37	20 (15.4)	17 (13.2)	
**CA72-4, U/ml**			
< 6.9	120 (92.3)	116 (89.9)	0.500
≥ 6.9	10 (7.7)	13 (10.1)	
**AFP, ng/ml**			
< 20	128 (98.5)	125 (96.9)	0.403
≥ 20	2 (1.5)	4 (3.1)	
**CA125, U/ml**			
< 35	127 (97.7)	126 (97.7)	0.992
≥ 35	3 (2.3)	3 (2.3)	

*AJCC* American Joint Committee on Cancer, *BMI* body mass index (calculated as weight in kilograms divided by height in meters squared), *ECOG* Eastern Cooperative Oncology, *LN* lymph node, *CEA* carcinoembryonic antigen, *CA199* carbohydrate antigen 19-9, *CA72-4* carbohydrate antigen 72-4, *AFP* alpha fetoprotein, *CA125* carbohydrate antigen 125, *SMA* submucosa approach, *SSA* subserosa approach, *SD* standard deviation

### Lymph node dissection

In the SSA group, ICG could be seen rapidly drained into the perigastric LNs after subserosal injection. Within the first 15 min, the fluorescence of LNs was gradually enhanced (Additional file 3: Fig. S3). Twenty minutes after injection, the fluorescence of the D2 station was stable and comparable to that of the SMA. The mean (SD) total number of retrieved LNs in the SMA and SSA groups was 49.8 (14.6) and 49.2 (14.0), respectively, with no significant difference (*P* = 0.713). At least 16 LNs were retrieved for all patients in both groups, and a total of ≥ 30 LNs were retrieved from 127 (97.7%) patients in the SMA group and 126 (97.7%) patients in the SSA group (*P* = 0.992). Further stratification showed that the mean total number of LN dissections in the SMA group was similar to that in the SSA group, regardless of the resection method, body mass index, tumor size, and cT or cN category (*P* > 0.05 for all; Table 2).

**Table 2:** Number of retrieved lymph nodes in the SMA and SSA groups

Variable	Mean (SD)/No. (%)		***P*** value
	SMA (***n*** = 130)	SSA (***n*** = 129)	
**Total retrieved LNs**	49.8 (14.6)	49.2 (14.0)	0.713
< 30	3 (2.3)	3 (2.3)	0.992
≥ 30	127 (97.7)	126 (97.7)	
**Surgical procedure**			
Distal gastrectomy	49.1 (13.2)	47.2 (12.6)	0.392
Total gastrectomy	50.6 (16.1)	51.1 (15.0)	0.859
**Age**			
≤ 60	50.6 (14.5)	49.7 (15.1)	0.738
> 60	49.0 (14.7)	48.6 (12.9)	0.860
**BMI, kg/m**^**2**^			
≤ 24	50.5 (13.4)	51.2 (14.3)	0.717
> 24	48.2 (17.0)	43.6 (11.4)	0.179
**Sex**			
Male	49.8 (13.7)	48.5 (14.1)	0.564
Female	49.9 (16.4)	50.5 (13.7)	0.867
**Tumor location**			
Upper	49.8 (14.0)	47.0 (12.2)	0.482
Middle	51.6 (17.7)	47.8 (12.1)	0.304
Lower	49.1 (13.4)	50.5 (15.3)	0.555
**Size, cm**			
≤4	48.9 (13.6)	50.7 (15.6)	0.441
> 4	51.0 (15.8)	46.3 (10.0)	0.086
**Histology**			
Differentiated	49.3 (13.4)	49.4 (14.5)	0.982
Undifferentiated	50.2 (15.4)	49.0 (13.6)	0.622
**Lymphvascular invasion**			
Negative	51.3 (14.5)	50.9 (14.9)	0.867
Positive	47.8 (14.5)	46.3 (11.8)	0.556
**cT category**			
cT1	49.9 (15.5)	52.2 (15.5)	0.488
cT2	47.3 (14.1)	50.2 (14.2)	0.539
cT3	46.8 (12.2)	45.7 (12.1)	0.675
cT4a	55.9 (15.4)	48.7 (13.2)	0.073
**cN category**			
cN0	49.5 (14.8)	52.1 (14.9)	0.365
cN+	50.0 (14.5)	46.8 (12.8)	0.158
**pT category**			
pT1	48.3 (12.2)	51.3 (15.7)	0.319
pT2	50.6 (13.6)	46.3 (12.2)	0.372
pT3	52.4 (16.6)	48.5 (13.8)	0.238
pT4a	46.6 (14.4)	47.7 (11.8)	0.783
**pN category**			
pN0	49.8 (14.7)	51.8 (14.7)	0.478
pN+	49.8 (14.6)	47.0 (13.0)	0.216
**LN confined to D2 lymphadenectomy**	49.1 (14.1)	48.1 (13.2)	0.555
**Surgical procedure**			
Distal gastrectomy	48.6 (13.2)	47.0 (12.4)	0.464
Total gastrectomy	49.7 (15.2)	49.3 (14.1)	0.856

*BMI* body mass index, *LN* lymph node, *SMA* submucosa approach, *SSA* subserosa approach, *SD* standard deviation

**Fig. 3:**
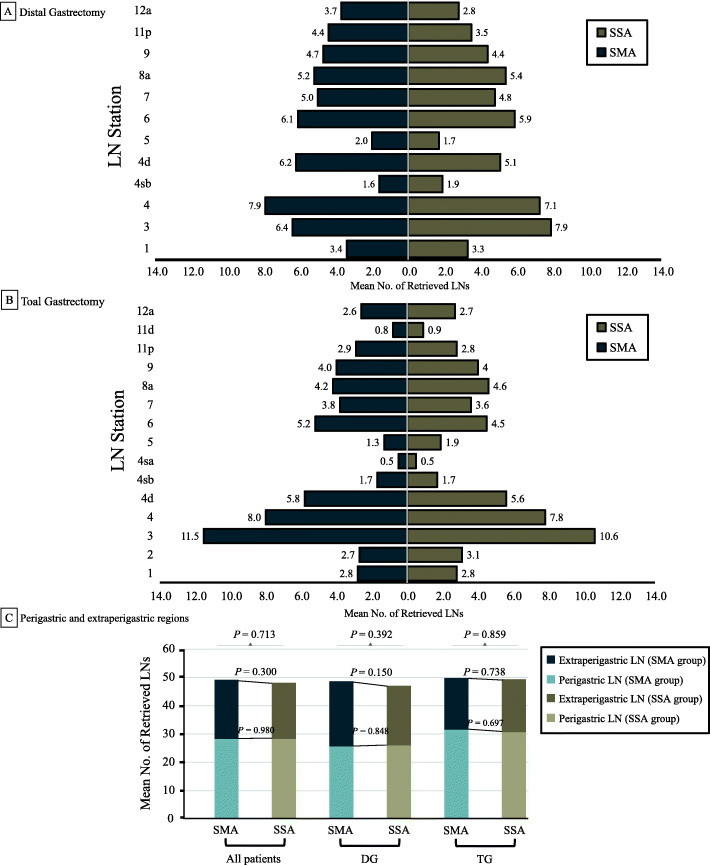
Total number of retrieved lymph nodes in the SMA and SSA groups by the lymph node station. **A** Distal gastrectomy. **B** Total gastrectomy. **C** Perigastric and extraperigastric regions. Perigastric lymph nodes at stations 1, 2, 3, 4, 5, and 6; extraperigastric lymph nodes at stations 7, 8, 9, 11, and 12a. SMA, submucosa approach; SSA, subserosa approach. DG, distal gastrectomy; TG, total gastrectomy

Figure 3 compares the number of LN dissections at each station in the SMA and SSA groups. There was no significant difference in the number of LN dissections between the two groups at the same station, regardless of the resection method. Regarding each LN region, including the perigastric (stations 1–6) and extraperigastric (stations 7–9, 11, and 12a) regions, no statistical difference was noted between the two groups.

In the SMA group, the number of fluorescent LNs was significantly higher than that of nonfluorescent LNs (mean [SD], 26.1 [11.9] vs. 23.7 [11.2]; *P* = 0.046). The mean number of LNs retrieved from fluorescent stations was significantly higher than that retrieved from nonfluorescent stations, regardless of the resection method [distal gastrectomy (DG), 5.8 vs. 1.3; total gastrectomy (TG), 5.3 vs. 2.0; *P* < 0.001 for both; Additional file 3: Fig. S4]. Similarly, in the SSA group, the total number of fluorescent LNs was significantly higher than that of nonfluorescent LNs (mean [SD], 28.9 [11.3] vs. 20.3 [9.2]; *P* < 0.001). Further analysis showed that the mean number of fluorescent LNs in the SMA group was similar to that in the SSA group at each station (Additional file 3: Fig. S5).

### Dissection extent

In D2 dissection, there were no between-group differences in the mean total number of retrieved LNs (mean [SD], 49.1 [14.1] in the SMA group vs. 48.1 [13.2] in the SSA group; *P* = 0.555). There was no significant difference in the mean total number of retrieved LNs between the SMA with SSA within the scope of D2 dissection, regardless of the resection method. Analysis of the extent of dissection (Additional file 2: Table S5) showed that the mean number of LNs dissected at the D1 station was comparable between the SMA and SSA groups (32.8 vs. 32.5; *P* = 0.860). The mean number of LNs dissected at the D1+ station and D2 station was also comparable between the SMA and SSA groups (D1+ station, 10.5 vs. 10.6, *P* = 0.667; D2 station, 5.9 vs. 5.0, *P* = 0.281).

### Lymph node noncompliance

For patients who underwent DG or TG, the LN dissection rates did not significantly differ between the SMA and SSA groups at each station (Additional file 3: Fig. S6). The LN noncompliance rate was comparable between the SMA and SSA groups (32.3% vs. 33.3%; *P* = 0.860). Subgroup analysis revealed that the LN noncompliance rate in the SMA and SSA groups among patients who underwent DG and TG was 26.8% vs. 27.7% (*P* = 0.903) and 39.0% vs. 39.1% (*P* = 0.993), respectively. In addition, there was no significant difference in the major LN noncompliance rate between the SMA and SSA groups (13.8% vs. 17.8%; *P* = 0.380; Additional file 2: Table S6).

### Lymph node metastasis

The number of metastatic LNs in each station in the SMA group was not significantly different from that in the SSA group, regardless of the resection method (Additional file 2: Table S7). Further analysis showed that there were no between-group differences in terms of the number of metastatic LNs, regardless of the D1, D1+, or D2 station (Additional file 2: Table S5). The sensitivity for the detection of metastatic LNs using fluorescent LNs in the SMA and SSA groups was 62.2% (333/535) and 58.0% (284/490), respectively (*P* = 0.161; Additional file 2: Table S8). The sensitivity for detecting metastatic stations using fluorescent lymphography in the SMA and SSA groups was 89.6% and 90.1%, respectively (*P* = 0.841; Additional file 2: Table S9).

### Surgical outcomes, recovery, and laboratory data

No significant differences between the SMA and SSA groups were found in terms of operative time (197.5 vs. 207.1 min; *P* = 0.112) and estimated blood loss (51.1 vs. 52.4 ml; *P* = 0.243; Table 3). The postoperative recovery courses, including time to first flatus, time to ambulation, time to first liquid intake, and length of postoperative hospital stay, were not significantly different between the two groups. Further stratification revealed that the recovery courses were comparable between the two groups, regardless of resection method (Additional file 2: Table S10). No delayed complications associated with NIR imaging of ICG injection were observed in either group. No significant differences were found between the SMA and SSA groups in terms of the incidence (17.7% vs. 16.3%; *P* = 0.762) or severity of postoperative complications.

**Table 3:** Surgical outcomes, postoperative recovery, morbidity, and mortality in the SMA and SSA groups

Outcome	Mean (SD)/No. (%)		***P*** Value
	SMA (***n*** = 130)	SSA (***n*** = 129)	
**Surgical outcome**			
Estimated blood loss (mL)	51.1 (46.2)	52.4 (59.7)	0.243
Surgical time (minutes)	197.5 (43.7)	207.1 (52.1)	0.112
**Postoperative recovery**			
Time to first flatus (days)	3.1 (1.1)	3.3 (0.7)	0.157
Time to ambulation (days)	2.1 (0.6)	2.2 (1.1)	0.580
Time to first liquid intake (days)	3.6 (1.0)	3.8 (1.3)	0.177
Time to first semifluid intake (days)	5.5 (1.9)	5.7 (4.0)	0.672
Postoperative hospital stays (days)	8.3 (5.0)	8.2 (5.6)	0.956
Postoperative transfusion	9 (6.9)	7 (5.4)	0.617
Reoperation^a^	1 (0.8)	0 (0.0)	0.318
Unplanned readmission^b^	1 (0.8)	3 (2.3)	0.310
**Morbidity type**			
Postoperative complication	23 (17.7)	21 (16.3)	0.762
Anastomotic			
Leakage	1 (0.8)	1 (0.8)	0.996
Stenosis	2 (1.5)	2 (1.6)	0.994
Wound problem	3 (2.3)	2 (1.6)	0.658
Intra-abdominal bleeding	1 (0.8)	0 (0.0)	0.318
Abdominal infection	3 (2.3)	3 (2.3)	0.992
Ileus	2 (1.5)	1 (0.8)	0.566
Lymphatic leakage	3 (2.3)	5 (3.9)	0.466
Gastroparesis	0 (0.0)	1 (0.8)	0.315
Pulmonary	12 (9.2)	11 (8.5)	0.842
Cerebrovascular	3 (2.3)	1 (0.8)	0.317
Deep vein thrombosis	2 (1.5)	0 (0.0)	0.157
Hepatic	2 (1.5)	2 (1.6)	0.994
Renal	1 (0.8)	1 (0.8)	0.996
Other	4 (3.1)	3 (2.3)	0.709
**Mortality**	0 (0.0)	0 (0.0)	-
**Clavien-Dindo classification**			
I	1 (0.8)	2 (1.6)	0.850
II	17 (13.1)	15 (11.6)	
IIIa	3 (2.3)	2 (1.6)	
IIIb	1 (0.8)	0 (0.0)	
IV	1 (0.8)	2 (1.6)	
V	0 (0.0)	0 (0.0)	

*LN* lymph node, *SMA* submucosa approach, *SSA* subserosa approach, *SD* standard deviation

^a^Due to one case of postoperative intra-abdominal hemorrhage in the SMA group

^b^Due to one case of postoperative ileus, one case of postoperative abdominal infection and one case of anastomotic stenosis in the SSA group; one case of postoperative abdominal infection in the SMA group

Regarding laboratory findings (Additional file 3: Fig. S7), there was no difference in leukocyte counts, hemoglobin levels, platelet counts, total bilirubin levels, and albumin levels between the SMA and SSA groups preoperatively and on postoperative days 1, 3, and 5.

### Cost-effectiveness and burden evaluation

The mean (SD) total cost during hospitalization was $9860.3 ($970.0) and $9640.6 ($1276.2) in the SMA and SSA groups, respectively, without a significant difference (*P* = .76; Additional file 2: Table S11). Regarding the fluorescence-related cost, the SMA costs $153 more per case than SSA ($335.3 vs. $182.4; *P* < 0.001).

The results of in-patient satisfaction showed lower overall general satisfaction scores in the SMA group than in the SSA group (70.5 vs. 76.1; *P* = 0.048; Additional file 2: Table S12). For special items, including repeat examinations and examination discomfort scores, the mean patient satisfaction score in the SMA group was lower than in the SSA group (63.2 vs. 72.8; *P* < 0.001).

The Surg-TLX score was similar in the SSA and SMA groups (36.6 vs. 36.4, *P* = 0.861). Surgeons experienced similar physical demands, mental demands, and task complexity while operating patients in both group (Additional file 2: Table S13).

## Discussion

To the best of our knowledge, this study is the first RCT comparing the efficacy of different ICG injection modalities for LN tracing during laparoscopic radical GC resection. Preoperative submucosal and intraoperative subserosal ICG injection were comparable in terms of the total number of retrieved LNs, LN noncompliance rates, operative time, and surgical burden. However, intraoperative subserosal ICG injection was associated with better patient satisfaction and lower fluorescence costs compared with preoperative submucosal ICG injection.

Within the specified dissection range, increasing the number of LN dissections and avoiding missed dissection of positive LNs retrieved are significantly associated with accurate staging, subsequent treatment options, and prognosis improvement of GC [19–21]. Therefore, it is important to thoroughly dissect perigastric LNs in resectable GC. Consistent with a previous study [4], we found that ICG fluorescence imaging-guided lymphadenectomy significantly improved the quality of LN dissection in GC. The number of LNs dissected was ≥ 30 in > 95% patients in both groups, and the LN dissection noncompliance rate in both groups was significantly lower than that reported in previous studies [22, 23]. Further analysis showed that the average number of fluorescent LNs detected was significantly higher than that of nonfluorescent LNs in both groups. Compared with the average number of LN dissection in the non-ICG group (42) in the previous study [4], we found that SMA (49.8) or SSA (49.2) in this study (Additional file 3: Fig. S8) can effectively increase the average number of LN dissection (*P* both < 0.001). This indicates that both injection methods are equally effective for LN tracing in D2 lymphadenectomy.

Several studies have suggested that the injection site of the LN tracer should not be limited to the submucosa [24, 25]. Jamieson and Dobson found that the lymphatic fluid flows from the submucosa into the subserosal plexus [26]. In our study, the injected tracer in the submucosa immediately stained the subserosa in postoperative specimens. The submucosa was also stained with a tracer injected into the subserosa in resected specimens. Further, consistent with the previous report [26, 27], our results inferred that submucosal lymphatic vessels are connected with subserosal lymphatic vessels through the intermuscular lymphatic network (Fig. 1C, Additional file 3: Fig. S9). It is postulated that the ICG injected into the submucosa around the tumor would likely disperse through the same route as that injected into the subserosal layer. Therefore, it is assumed that there is no difference in LN dissection results using the SMA or SSA. Moreover, the accuracy of fluorescent lymphography for detecting metastatic stations was comparable between the two methods.

Tajima et al. [28] found that intraoperative subserosal injection was less accurate than preoperative submucosal injection of ICG for detecting sentinel LNs. Moreover, some retrospective studies [29, 30] support the use of submucosal injection of ICG for fluorescence-guided lymphadenectomy the day before surgery. These inconsistent results may be explained by the selection bias inherent in retrospective investigations and varying injection sites, time, and concentrations of ICG used across studies. Therefore, we proposed Huang’s subserosal hexa-points maneuver according to the drainage characteristics of perigastric LNs and the criteria for D2 lymphadenectomy [8, 31]. It overcomes the shortcomings of the traditional four-point peritumor subserosal injection, in which it is challenging to identify the tumor location from the outside of the stomach without intraoperative localization of the tumor, especially in early GC cases [6, 32]. We found that stable and good LN visualization can be achieved at the D2 station after 20 min of subserosal injection. Because the surgeon can perform the essential omental separation and perigastric adhesion separation during this waiting period, our results showed that intraoperative subserosal injection conducted in this way does not significantly increase the total operative time. Therefore, ICG injection followed by sequential lymphadenectomy is easy for the surgeon to control. It will not interfere with the routine operation procedure while ensuring clear fluorescence images.

Patients often experience nausea, vomiting, and coughing during routine gastroscopy. In our study, patients in the SSA group had a better hospital experience than those in the SMA group. Intraoperative subserosal injection is effective in reducing patient anxiety and discomfort compared to preoperative endoscopic submucosal injection. Efficient use of medical resources to provide patients with cost-effective medical solutions has been the new quest in the era of patient-centered precision surgery [33]. Cost-effectiveness analysis has shown that intraoperative subserosal injection as part of a complete procedure can significantly reduce the fluorescence-related cost and workload of endoscopists while achieving comparable LN tracing compared to preoperative submucosal injection. In addition, subserosal injection is a convenient method in surgical centers that do not routinely perform therapeutic gastroscopy, which suits the operation of the surgeon and facilitates the promotion of fluorescence imaging technology. Moreover, for patients with early GC (cT1) who need preoperative endoscopic localization, ICG submucosal injection can go together with preoperative endoscopic localization to efficiently save time in practical application.

We found that among the 259 patients included in the primary analysis of this study, 123 patients underwent ICG fluorescence imaging-guided laparoscopic TG, with an average of 50.9 LNs retrieved, while 136 patients underwent ICG fluorescence imaging-guided laparoscopic DG, with an average of 48.2 LNs retrieved. The number of LNs retrieved in the patients who underwent TG was 2.7 more than those who underwent DG. This is similar to the results of previous studies [4, 34–36]. We also found that, whether DG or TG, the most retrieved LNs were mainly in the infrapyloric area and the suprapancreatic area. In addition, the total number of LNs dissection in patients with gastric cancer has been significantly increased by the use of ICG fluorescence imaging. Compared with the total LNs retrieved (approximately 50), the average difference of 2.7 may not appear that significant. This may be the reason why the number of LNs retrieved in patients who underwent ICG fluorescence imaging-guided laparoscopic TG is not much higher than that in patients who underwent ICG fluorescence imaging-guided laparoscopic DG.

This study has several limitations. First, although the study results showed that both the injection methods were effective in guiding LN dissection, the effect of different injection methods on long-term survival needs to be confirmed. Second, this study was conducted at high-volume referral centers with extensive experience in the surgical treatment of GC, and more future research are needed to solidly establish the sound generalizability of the findings to other centers with different levels of experience. Third, this RCT did not include patients who received neoadjuvant therapy, and patients often have tumor and LN regression and fibrotic response after neoadjuvant therapy. The role of ICG fluorescence imaging-guided surgery in patients who have undergone neoadjuvant therapy need to be further explored. Fourth, ICG is a dye that appears green under natural light [37], which can be clearly distinguished from almost colorless normal saline, a crystalloid solution. At present, there is no well-recognized safe and effective placebo with the same color as ICG approved by FDA for intragastric injection, so it is difficult for endoscopists and surgeons to make blind allocation during operation. This study was not only conducted to compare the efficacy, safety of the SMA, and intraoperative SSA for ICG injection for LN tracing during laparoscopic gastrectomy in patients with GC, but also aimed to evaluate the cost-effectiveness of the two approaches. Therefore, if both groups of patients underwent endoscopy the day before surgery, it cannot truly reflect the impact of the two injection approaches on the treatment experience and the economic burden of patients. Finally, similar to a previous study [28], ICG fluorescence imaging could not accurately indicate metastatic LNs with either subserosal or submucosal injections.

## Conclusions

Among patients with GC, intraoperative subserosal injection of ICG was comparable to preoperative submucosal injection of ICG during laparoscopic fluorescence imaging-guided lymphadenectomy, and the former approach resulted in better patient satisfaction and was cost-effective compared to the latter approach. Subserosal injection of ICG may be a reasonable option for fluorescent lymphography-guided D2 lymphadenectomy in patients with GC.

## Supplementary Information

**Additional file 1.** FUGES-019 Study Protocol.

**Additional file 2:** Table S1-Table S13.

**Additional file 3:** Fig. S1-Fig. S9.

**Additional file 4.** Detailed method.

**Additional file 5:** Video. Part 1. EndoscopicAQ6 Injection of Indocyanine Green Tracer One Day Before Surgery. Part 2. Preoperative Preparation Before Laparoscopic Subserosal Injection of Indocyanine Green Tracer. Part 3. Huang’s Subserosal Hexa-Points Maneuver for the Injection of Indocyanine Green Tracer During LaparoscopicTotal Gastrectomy. Part 4. Indocyanine Green Fluorescence Imaging-guided Lymphadenectomy During Laparoscopic Radical Gastrectomy for Gastric Cancer. Part 5. Indocyanine Green Fluorescence Imaging-guided Complementary Dissection of Residual Lymph Node After Routine Lymphadenectomy for Gastric Cancer. Part 6. Lymph Node Retrieval In Vitro During Direct Near-Infrared (NIR) Imaging After Laparoscopic Gastrectomy and Tracer-guided Lymph Node Dissection for Gastric Cancer.

## Data Availability

The datasets used and/or analyzed during the current study are available from the corresponding author on reasonable request.

## Abbreviations

DG: Distal gastrectomy
FUGES: Fujian Medical University Union Hospital Gastric Surgery Study
GC: Gastric cancer
ICG: Indocyanine green
LN: Lymph node
RCT: Randomized controlled trial
SMA: Submucosal approach
SSA: Subserosal approach
TG: Total gastrectomy

## References

[1] Patti MG, Herbella FA (2020). Indocyanine green tracer-guided lymph node retrieval during radical dissection in gastric cancer surgery. JAMA Surg.

[2] Liu M, Xing J, Xu K, Yuan P, Cui M, Zhang C, Yang H, Yao Z, Zhang N, Tan F, Su X (2020). Application of near-infrared fluorescence imaging with indocyanine green in totally laparoscopic distal gastrectomy. J Gastric Cancer.

[3] Roh CK, Choi S, Seo WJ, Cho M, Son T, Kim HI, Hyung WJ (2020). Indocyanine green fluorescence lymphography during gastrectomy after initial endoscopic submucosal dissection for early gastric cancer. Br J Surg.

[4] Chen QY, Xie JW, Zhong Q, Wang JB, Lin JX, Lu J, Cao LL, Lin M, Tu RH, Huang ZN, Lin JL, Zheng HL, Li P, Zheng CH, Huang CM (2020). Safety and efficacy of indocyanine green tracer-guided lymph node dissection during laparoscopic radical gastrectomy in patients with gastric cancer: a randomized clinical trial. JAMA Surg.

[5] Stamenkovic DM, Rancic NK, Latas MB, Neskovic V, Rondovic GM, Wu JD, Cattano D (2018). Preoperative anxiety and implications on postoperative recovery: what can we do to change our history. Minerva Anestesiol.

[6] Herrera-Almario G, Patane M, Sarkaria I, Strong VE (2016). Initial report of near-infrared fluorescence imaging as an intraoperative adjunct for lymph node harvesting during robot-assisted laparoscopic gastrectomy. J Surg Oncol.

[7] Baiocchi GL, Molfino S, Molteni B, Quarti L, Arcangeli G, Manenti S, Arru L, Botticini M, Gheza F (2020). Fluorescence-guided lymphadenectomy in gastric cancer: a prospective western series. Updates Surg.

[8] Japanese Gastric Cancer Association. Japanese gastric cancer treatment guidelines 2018 (5th edition). Gastric Cancer. 2021;24(1):1–21. 10.1007/s10120-020-01042-y. 10.1007/s10120-020-01042-y PMC7790804 32060757

[9] Huang CM, Zheng CH. Laparoscopic gastrectomy for gastric cancer. Springer; 2015. 10.1007/978-94-017-9873-0.

[10] Association JGC (2011). Japanese classification of gastric carcinoma: 3rd English edition. Gastric Cancer.

[11] de Steur WO, Hartgrink HH, Dikken JL, Putter H, van de Velde CJ (2015). Quality control of lymph node dissection in the Dutch Gastric Cancer Trial. Br J Surg.

[12] Kakar S, Pawlik T, Allen P (2017). AJCC cancer staging manual.

[13] Dindo D, Demartines N, Clavien PA (2004). Classification of surgical complications: a new proposal with evaluation in a cohort of 6336 patients and results of a survey. Ann Surg.

[14] Brédart A, Bottomley A, Blazeby JM, Conroy T, Coens C, D'Haese S, Chie WC, Hammerlid E, Arraras JI, Efficace F (2005). An international prospective study of the EORTC cancer in-patient satisfaction with care measure (EORTC IN-PATSAT32). Eur J Cancer.

[15] Singer E, Kneuertz PJ, D'Souza DM, Moffatt-Bruce SD, Merritt RE (2019). Understanding the financial cost of robotic lobectomy: calculating the value of innovation?. Ann Cardiothorac Surg.

[16] Lu J, Zheng CH, Xu BB, Xie JW, Wang JB, Lin JX, Chen QY, Cao LL, Lin M, Tu RH, Huang ZN, Lin JL, Zheng HL, Huang CM, Li P (2021). Assessment of robotic versus laparoscopic distal gastrectomy for gastric cancer: a randomized controlled trial. Ann Surg.

[17] Wilson MR, Poolton JM, Malhotra N, Ngo K, Bright E, Masters RS (2011). Development and validation of a surgical workload measure: the surgery task load index (SURG-TLX). World J Surg.

[18] Schulz KF, Altman DG, Moher D (2011). CONSORT 2010 statement: updated guidelines for reporting parallel group randomised trials. Int J Surg.

[19] Smith DD, Schwarz RR, Schwarz RE (2005). Impact of total lymph node count on staging and survival after gastrectomy for gastric cancer: data from a large US-population database. J Clin Oncol.

[20] Son T, Hyung WJ, Lee JH, Kim YM, Kim HI, An JY, Cheong JH, Noh SH (2012). Clinical implication of an insufficient number of examined lymph nodes after curative resection for gastric cancer. Cancer.

[21] Woo Y, Goldner B, Ituarte P, Lee B, Melstrom L, Son T, Noh SH, Fong Y, Hyung WJ (2017). Lymphadenectomy with optimum of 29 lymph nodes retrieved associated with improved survival in advanced gastric cancer: a 25,000 patient international database study. J Am Coll Surg.

[22] Claassen YHM, de Steur WO, Hartgrink HH, Dikken JL, van Sandick JW, van Grieken NCT, Cats A, Trip AK, Jansen EPM, Kranenbarg WMM (2018). Surgicopathological quality control and protocol adherence to lymphadenectomy in the CRITICS gastric cancer trial. Ann Surg.

[23] Park YK, Yoon HM, Kim YW, Park JY, Ryu KW, Lee YJ, Jeong O, Yoon KY, Lee JH, Lee SE, Yu W, Jeong SH, Kim T, Kim S, Nam BH, COACT group (2018). Laparoscopy-assisted versus open D2 distal gastrectomy for advanced gastric cancer: results from a randomized phase II multicenter clinical trial (COACT 1001). Ann Surg.

[24] Yaguchi Y, Ichikura T, Ono S, Tsujimoto H, Sugasawa H, Sakamoto N, Matsumoto Y, Yoshida K, Kosuda S, Hase K (2008). How should tracers be injected to detect for sentinel nodes in gastric cancer--submucosally from inside or subserosally from outside of the stomach?. J Exp Clin Cancer Res.

[25] Lee JH, Ryu KW, Kim CG, Kim SK, Choi IJ, Kim YW, Chang HJ, Bae JM, Hong EK (2005). Comparative study of the subserosal versus submucosal dye injection method for sentinel node biopsy in gastric cancer. Eur J Surg Oncol.

[26] Jamieson JK, Dobson JF (1907). Lectures on the lymphatic system of the stomach. Lancet.

[27] Coller F. Regional lymphatic metastasis of carcinoma of the stomach. Arch Surg (Chicago, Ill : 1960). 1941;43:748–61.

[28] Tajima Y, Yamazaki K, Masuda Y, Kato M, Yasuda D, Aoki T, Kato T, Murakami M, Miwa M, Kusano M (2009). Sentinel node mapping guided by indocyanine green fluorescence imaging in gastric cancer. Ann Surg.

[29] Jung MK, Cho M, Roh CK, Seo WJ, Choi S, Son T, Kim HI, Hyung WJ (2021). Assessment of diagnostic value of fluorescent lymphography-guided lymphadenectomy for gastric cancer. Gastric Cancer.

[30] Cianchi F, Indennitate G, Paoli B, Ortolani M, Lami G, Manetti N, Tarantino O, Messeri S, Foppa C, Badii B, Novelli L, Skalamera I, Nelli T, Coratti F, Perigli G, Staderini F (2020). The clinical value of fluorescent lymphography with indocyanine green during robotic surgery for gastric cancer: a matched cohort study. J Gastrointest Surg.

[31] Lirosi MC, Biondi A, Ricci R (2017). Surgical anatomy of gastric lymphatic drainage. Transl Gastroenterol Hepatol.

[32] Lan YT, Huang KH, Chen PH, Liu CA, Lo SS, Wu CW, Shyr YM (2017). Fang WLJSOM, 5,: A pilot study of lymph node mapping with indocyanine green in robotic gastrectomy for gastric cancer. SAGE Open Med.

[33] Kasztura M, Richard A, Bempong NE, Loncar D, Flahault A (2019). Cost-effectiveness of precision medicine: a scoping review. Int J Public Health.

[34] Lin JX, Huang CM, Zheng CH, Li P, Xie JW, Wang JB, Lu J, Chen QY, Lin M, Tu R (2016). Evaluation of laparoscopic total gastrectomy for advanced gastric cancer: results of a comparison with laparoscopic distal gastrectomy. Surg Endosc.

[35] Chen QY, Lin GT, Zhong Q, Zheng CH, Li P, Xie JW, Wang JB, Lin JX, Lu J, Cao LL, Huang CM (2020). Laparoscopic total gastrectomy for upper-middle advanced gastric cancer: analysis based on lymph node noncompliance. Gastric Cancer.

[36] Li Z, Ji G, Bai B, Yu D, Liu Y, Lian B, Zhao Q (2018). Laparoscopy-assisted distal gastrectomy versus laparoscopy-assisted total gastrectomy with D2 lymph node dissection for middle-third advanced gastric cancer. Surg Endosc.

[37] Reinhart MB, Huntington CR, Blair LJ, Heniford BT, Augenstein VA (2016). Indocyanine green: historical context, current applications, and future considerations. Surg Innov.

