# Supplementary material for: Comparison of submucosal and subserosal approaches toward optimized indocyanine green tracer-guided laparoscopic lymphadenectomy for patients with gastric cancer (FUGES-019): a randomized controlled trial
Source: BMC Med. 2021 Oct 27;19:276. doi: 10.1186/s12916-021-02125-y (PMC8549272; doi:10.1186/s12916-021-02125-y)
Supplement: Supplementary file 1 — Additional file 1. FUGES-019 Study Protocol. [file 12916_2021_2125_MOESM1_ESM.pdf]

Submucosal versus Subserosal Approaches of Indocyanine Green Tracer-Guided Lymphadenectomy for Gastric Cancer: A Randomized Clinical Trial

Study Protocol

Bidding party: Fujian Medical University Union Hospital

Principle Investigator:

Prof. Chang-Ming Huang, M.D.

Department of Gastric Surgery, Fujian Medical University Union Hospital,

Address: No. 29 Xinquan Road, Fuzhou 350001 Fujian Province, China.

Telephone: +86-591-83363366, Fax: +86-591-83363366

No. of edition: V1.1

The date of the edition: 2019.11.01

Summary

Scenario Title	Comparison of Submucosal and Subserosal Approaches Toward Optimized Indocyanine Green Tracer-Guided Laparoscopic Lymphadenectomy for Patients with Gastric Cancer: The FUGES-019 Randomized Clinical Trial
Scenario Version	V1.1
Sponsor	Chang-Ming Huang
Research Center	Fujian Medical University Union Hospital
Indications	Patients with potentially resectable gastric adenocarcinoma (cT1-4a, N0/+, M0)
Purpose of research	To compare the efficacy and cost-effectiveness of the submucosal approach (SMA) and subserosal approach (SSA) of ICG injection for lymph node (LN) tracing during radical gastrectomy in patients with gastric cancer.
Research design	Investigator-initiated, single-center, parallel, noninferiority, open-label, randomized controlled
Case grouping	Group A (Study Group): Laparoscopic gastrectomy with subserosal injection of indocyanine green tracer group (SMA group)<br>Group B (Control Group): Laparoscopic gastrectomy with submucosal injection of indocyanine green tracer group (SSA group)
The basis for determining the sample size	This study is a noninferiority test (bilateral), whose primary outcome measure is the total number of retrieving LNs. According to the previous study results, the total number of LNs retrieved in the control group was about 50.5 with a standard deviation was 15.9. A sample size of 111 patients per group was calculated as necessary for 80% power to detect a noninferiority margin of 6 with a 1-sided α of 0.025. Assuming an expected dropout rate of 20%, each group needed to include at least 133 patients, for a total of 266 cases.
Inclusion criteria	● Age from 18 to 75 years (not including 18 and 75 years old)<br>● Primary gastric adenocarcinoma (papillary, tubular, mucinous, signet ring cell, or poorly differentiated) confirmed pathologically by endoscopic biopsy<br>● A tumor stage of T1-4a (cT1-4a), N0/+, M0 at preoperative evaluation according to the American Joint Committee on Cancer (AJCC) Cancer Staging Manual Eighth Edition<br>● No distant metastasis, no direct invasion of pancreas, spleen, or other

	organs nearby in the preoperative examinations<br>● Performance status of 0 or 1 on Eastern Cooperative Oncology Group scale (ECOG)<br>● American Society of Anesthesiology score (ASA) class I, II, or III<br>● Written informed consent
Exclusion criteria	● Women during pregnancy or breast-feeding<br>● Severe mental disorder<br>● History of previous upper abdominal surgery (except laparoscopic cholecystectomy)<br>● History of previous gastrectomy, endoscopic mucosal resection, or endoscopic submucosal dissection<br>● Rejection of laparoscopic resection<br>● History of allergy to iodine agents<br>● Enlarged or bulky regional LN diameter over 3cm by preoperative imaging<br>● History of other malignant diseases within the past five years<br>● History of previous neoadjuvant chemotherapy or radiotherapy<br>● History of unstable angina or myocardial infarction within the past six months<br>● History of a cerebrovascular accident within the past six months<br>● History of continuous systematic administration of corticosteroids within one month<br>● Requirement of simultaneous surgery for other diseases<br>● Emergency surgery due to complication (bleeding, obstruction, or perforation) caused by gastric cancer<br>● Forced expiratory volume in 1 second (FEV1) < 50% of predicted values<br>● Linitis plastica, Widespread
Rejection criteria	● M1 tumor confirmed intraoperatively or postoperatively: distant metastasis only found by intraoperative exploration or postoperative pathological biopsy or a positive postoperative peritoneal lavage cytology examination;<br>● Patients intraoperatively/postoperatively confirmed as T4b, or tumor invading the duodenum;<br>● Patients intraoperatively confirmed as unable to complete D2 LN dissection/R0 resection due to tumor: unable to complete R0 resection due to regional LN integration into a mass or surrounded with important blood vessels, which cannot be resected;

	● Patients requiring simultaneous surgical treatment of other diseases;<br>● Patients with sudden severe comorbidities (cannot tolerate surgery or anesthesia) or in whom the procedure was unable to be implemented as planned after being selected;<br>● Patients confirmed to require emergency surgery by attending physicians due to changes in the patient's condition after inclusion in this study;<br>● Patients who voluntarily quit or discontinued treatment for personal reasons at any stage after inclusion in this study;<br>● LNs that were indistinguishable from ICG-stained soft tissue under NIR imaging due to ICG contamination;<br>● Treatment implemented that was proven to violate study protocol.
Intervention	● For patients who were assigned to the SSA group, after preoperative exploration, the indocyanine green powder (Dandong Yichuang Pharmaceutical Co) is dissolved in 0.5 mg/ml of sterile water and the prepared solution (1.5 ml for each point) is injected along the subserosal of the stomach at 6 specific points along the lesser and greater curvature of the stomach.<br>● For patients who were assigned to the SMA group, submucosal injection of indocyanine green One day before surgery, 1.25 mg/ml indocyanine green (Dandong Yichuang Pharmaceutical Co) was prepared in sterile water and 0.5 ml of the solution was injected into the submucosal layer at 4 quadrants around the primary tumor, amounting to 2.5 mg of indocyanine green.
Outcome Measures	**Primary Outcome Measures**:<br>● Total number of retrieved LNs<br>**Secondary Outcome Measures**:<br>● Total number of fluorescent LNs in groups A and B<br>● True positive rate<br>● False positive rate<br>● True negative rate<br>● False negative rate<br>● Number of metastasis LNs<br>● Metastasis rate of LN<br>● Morbidity and mortality rates<br>● 3-year disease free survival rate<br>● 3-year recurrence pattern<br>● Time to first ambulation

	• Time to first flatus<br>• Time to first liquid diet,<br>• Time to first soft diet<br>• Duration of postoperative hospital stay<br>• The variation of BMI<br>• The variation of weight<br>• Intraoperative morbidity rates<br>• The variation of album<br>• The variation of white blood cell count<br>• The variation of hemoglobin<br>• The variation of C-reactive protein<br>• LN noncompliance rate<br>• Modified EORTC cancer in-patient satisfaction with care measure (EORTC IN-PATSAT14)<br>• The Surgery Task Load Index (SURG-TLX)
Statistical considerations	All data analyses will be performed using the SPSS statistical software (version 22.0; SPSS Inc.) and R software (version 3.6.1; R Foundation for Statistical Computing).<br>The noninferiority analysis for the primary endpoint of the total number of retrieving LNs will be conducted, while the test method of difference for secondary endpoints. All the statistical tests were tested by two sides. A p-value <0.05 is considered statistically significant. The confidence interval of the parameters is estimated with a 95% confidence interval. Baseline data and validity analyses will be conducted on a modified intent-to-treat (MITT) and per-protocol (PP) basis. SAP analysis is used for safety assessment, and this study does not fill in missing values. Normally distributed continuous variables will be presented as mean and standard deviation and compared using the t-test if normally distributed, or as median and interquartile range and compared using the Wilcoxon rank-sum test if non-normally distributed; while categorical data will be presented as number and percentages and compared using the Pearson $\chi 2$ test or the Fisher exact test, as appropriate. Survival data will be analyzed using the Kaplan-Meier method and Cox's proportional hazards model. Sensitivity analysis is used for extreme outlier data. The subgroup analysis is conducted according to the specific situation. Interim analysis will not be conducted in this study.

1. Research background

Since Kitano[1] in Japan first reported laparoscopic distal gastrectomy for GC in 1994, after more than 20 years of development, laparoscopic radical gastrectomy has been widely used in clinical practice.[2-4] Nowadays, lymphadenectomy is often performed under the naked eye according to the surgeon's experience. However, due to the complex vascular anatomy and lymphatic drainage around the stomach, it remains a huge challenge for surgeons, especially young surgeons, to dissect enough lymph nodes (LNs) efficiently and accurately without increasing operate-related complications. Therefore, with the advent of the era of precision minimally invasive surgery, laparoscopic surgeons are still exploring how to perform convenient and accurate real-time LN navigation under laparoscope, to perform systematic, accurate, and sufficient LN dissection. As a new surgical navigation technique, indocyanine green (ICG) near-infrared (NIR) fluorescent imaging has achieved relatively positive results in the localization of sentinel LN in breast cancer, non-small-cell lung cancer, and other cancers.[5-8] With the successful application of ICG fluorescence imaging technology in laparoscopic devices, scholars have found that NIR imaging has better tissue penetration and can better identify LNs in hypertrophic adipose tissue than other dyes in visible light.[9,10] It has important research value, good application prospect, and broad development space, which has attracted wide attention, so that ICG fluorescence imaging-guided minimally invasive treatment such as laparoscopic or robotic radical resection of GC has become a new exploration direction.[11] Our randomized controlled trial (RCT) confirmed that ICG fluorescent lymphography-guided lymphadenectomy can help surgeons harvest more LNs than conventional LN dissection and reduce LN noncompliance in patients with gastric cancer who undergo D2 lymphadenectomy, and indocyanine green fluorescence imaging can be used for routine lymphatic mapping during laparoscopic gastrectomy, especially total gastrectomy.

However, at present, the application of ICG in laparoscopic lymphadenectomy for patients with gastric cancer is in the preliminary stages. In particular, there is no unified standard for ICG injection. At present, there are great differences in the preoperative submucosal or intraoperative subserosal injection methods in each center. Different methods of ICG injection used in different studies are also important reasons for the inconsistent results of ICG related clinical studies. Some scholars[12-14] believe that compared with intraoperative subserosal injection of ICG, preoperative submucosal

injection of ICG under endoscopic guidance one day before surgery was easier to control and saved time, and it was less likely to cause intraoperative leakage of ICG into the surgical field or to interfere with the operation by straying into blood vessels. Some studies[15,16] have shown that the injection under the serosa is easier for surgeons to control and the injection time can be easily unified.

Therefore, there is still a lack of high-level evidence-based large sample RCT to compare the safety, efficacy, and feasibility of preoperative submucosal or intraoperative subserosal injection of ICG on LN tracing and its economic and social benefits in laparoscopic radical gastrectomy worldwide. So, Fujian Medical University Union Hospital Gastric Surgery Study Group (FUGES) first conduct this RCT to assess LN harvest and perioperative safety during laparoscopic ICG-guide radical gastrectomy for GC patients by comparing the preoperative submucosal approach (SMA) and the intraoperative subserosal approach (SSA) of ICG injection at a tertiary referral teaching hospital.

2. Objective

The purpose of this study was to compare the effect of submucosal or subserosal injection of ICG on LN tracing and its economic and social benefits in laparoscopic radical gastrectomy.

3. Research design

Investigator-initiated, single-center, prospective, noninferiority, open-label, phase 3, parallel assignment, randomized controlled.

3.1 Single center

Department of Gastric Surgery, Fujian Medical University Union Hospital

3.2 Case group

Group A (Study Group): Laparoscopic gastrectomy with subserosal injection of indocyanine green tracer group (SMA group)

Group B (Control Group): Laparoscopic gastrectomy with submucosal injection of indocyanine green tracer group (SSA group)

3.3 Estimate Sample Size

This study is a noninferiority test (bilateral), whose primary outcome measure is the total number of retrieving LNs. According to the previous study results, the total number of LNs retrieved in the control group was about 50.5 with a standard deviation was 15.9. A sample size of 111 patients per group was calculated as necessary for 80% power to detect a noninferiority margin of 6 with a 1-sided α of 0.025. Assuming an expected dropout rate of 20%, each group needed to include at least 133 patients, for a total of 266 cases.

3.4 Blind method: This research adopts an open design. Although it is not feasible to blind the surgeons and participants, the pathologists are unaware of the intervention received by the patients. The researcher performing statistical analyses after recruitment is blinded to patient group allocation.

3.5 Research cycle

Estimated enrollment cycle: complete enrollment within 4 years

Follow-up period: begin at the enrollment of the first case and end 1 month after the enrollment of the last case.

Estimated time: 2019.12-2020.12 (to complete enrollment) - 2023.12 (to complete follow-up)

4. Study objects

All patients who meet the inclusion criteria and do not conform to the exclusion criteria are qualified for this study.

4.1 Inclusion criteria

(1) Age from 18 to 75 years

(2) Primary gastric adenocarcinoma (papillary, tubular, mucinous, signet ring cell, or poorly differentiated) confirmed pathologically by endoscopic biopsy

(3) A tumor stage of T1-4a (cT1-4a), N0/+, M0 at preoperative evaluation according to the American Joint Committee on Cancer (AJCC) Cancer Staging Manual Eighth Edition. Preoperative staging was made by conducting mandatory computed tomography (CT) scans and an optional endoscopic ultrasound

(4) No distant metastasis, no direct invasion of pancreas, spleen, or other organs nearby in the preoperative examinations

(5) Performance status of 0 or 1 on Eastern Cooperative Oncology Group scale (ECOG)

(6) American Society of Anesthesiology score (ASA) class I, II, or III

(7) Written informed consent

4.2 Exclusion criteria

(1) Women during pregnancy or breast-feeding

(2) Severe mental disorder

(3) History of previous upper abdominal surgery (except laparoscopic cholecystectomy)

(4) History of previous gastrectomy, endoscopic mucosal resection, or endoscopic submucosal dissection

(5) Rejection of laparoscopic resection

(6) History of allergy to iodine agents

(7) Enlarged or bulky regional LN diameter over 3cm by preoperative imaging

(8) History of other malignant diseases within the past five years

(9) History of previous neoadjuvant chemotherapy or radiotherapy

(10) History of unstable angina or myocardial infarction within the past six months

(11) History of a cerebrovascular accident within the past six months

(12) History of continuous systematic administration of corticosteroids within one month

(13) Requirement of simultaneous surgery for other diseases

(14) Emergency surgery due to complication (bleeding, obstruction, or perforation) caused by gastric cancer

(15) Forced expiratory volume in 1 second (FEV1) < 50% of predicted values

(16) Linitis plastica, Widespread

4.3 Rejection criteria

- M1 tumor confirmed intraoperatively or postoperatively: distant metastasis only found by intraoperative exploration or postoperative pathological biopsy or a positive postoperative peritoneal lavage cytology examination;
- Patients intraoperatively/postoperatively confirmed as T4b, or tumor invading the duodenum;
- Patients intraoperatively confirmed as unable to complete D2 LN dissection/R0 resection due to tumor: unable to complete R0 resection due to regional LN integration into a mass or surrounded with important blood vessels, which cannot be resected;
- Patients requiring simultaneous surgical treatment of other diseases;
- Patients with sudden severe comorbidities (cannot tolerate surgery or anesthesia) or in whom the procedure was unable to be implemented as planned after being selected;
- Patients confirmed to require emergency surgery by attending physicians due to changes in the patient's condition after inclusion in this study;
- Patients who voluntarily quit or discontinued treatment for personal reasons at any stage after inclusion in this study;
- LNs that were indistinguishable from ICG-stained soft tissue under NIR imaging due to ICG contamination;
- Treatment implemented that was proven to violate study protocol.

4.4 Case screening

(1) When Patients admitted to hospital should meet the following criteria: Age between 18 and 75 years old； Performance status of 0 or 1 on the ECOG scale； None-pregnant or no lactating women；Not suffering from a severe mental disorder； No history of previous upper abdominal surgery (except for laparoscopic cholecystectomy)； No history of previous gastric surgery (including ESD/EMR for gastric cancer)； No History of other malignant diseases within the past five years； No history of unstable angina or myocardial infarction within the past six months;

No history of continuous systematic administration of corticosteroids within one month; No requirement of simultaneous surgery for another disease; FEV1≥50% of the predicted values; No history of a cerebrovascular accident within the past six months.

(2) Endoscopic examination of the primary lesion in the patient (recommended endoscopic ultrasound endoscopy, EUS) and histopathological biopsy showed gastric adenocarcinoma (papillary adenocarcinoma [pap], tubular adenocarcinoma [tub], mucinous adenocarcinoma [muc], signet ring cell carcinoma [sig], and poorly differentiated adenocarcinoma [por]). Total abdominal CT was performed on the patient, and no enlarged LNs (maximum diameter ≥ 3 cm) were found in the periplasmic area, including significant enlargement or merging of the No. 10 LNs into a group or local invasion/distance metastasis. No obvious tumor infiltration was found in the spleen and spleen vessels.

(3) Patient is explicitly diagnosed with upper third gastric cancer, has a preoperative staging assessment of T1-4a, N0-3, M0 and is expected to undergo total gastrectomy and D2 LN dissection to obtain R0 surgical results (also indicated for multiple primary cancer).

(4) Patients do not require neoadjuvant chemoradiotherapy or chemotherapy and the attending doctor does not recommend that they receive neoadjuvant chemoradiotherapy or chemotherapy. ASA class I to III.

(5) No requirement for emergency surgery.

(6) Patient does not require emergency surgery.

(7) At this point the patient becomes a potential selected case and enters the 9.1 case selection procedure

5. Outcome Measures

5.1 Primary Outcome Measures

- Total number of retrieved LNs

5.2 Secondary Outcome Measures

- Total number of fluorescent LNs in groups A and B
- True positive rate
- False positive rate
- True negative rate
- False negative rate
- Number of metastasis LNs
- Metastasis rate of LN
- Morbidity and mortality rates
- 3-year disease free survival rate

- 3-year recurrence pattern
- Time to first ambulation
- Time to first flatus
- Time to first liquid diet
- Time to first soft diet
- Duration of postoperative hospital stay
- The variation of BMI
- The variation of weight
- Intraoperative morbidity rates
- The variation of album
- The variation of white blood cell count
- The variation of hemoglobin
- The variation of C-reactive protein
- LN noncompliance rate
- Modified EORTC cancer in-patient satisfaction with care measure (EORTC IN-PATSAT14)
- The Surgery Task Load Index (SURG-TLX)

6. Diagnostic criteria for this study

(1) The AJCC-8th TNM tumor staging system will be used for this study.

(2) Diagnostic criteria and classification of gastric cancer: According to the histopathological international diagnostic criteria, classification will be divided into papillary adenocarcinoma (pap), tubular adenocarcinoma (tub), mucinous adenocarcinoma (muc), signet ring cell carcinoma (sig), and poorly differentiated adenocarcinoma (por).

7 Qualifications of the participated Surgeons

7.1 Basic principle

All candidate surgeons in our study met the following criteria:

Performed at least 100 laparoscopic radical gastrectomy.

Pass the blind surgical video examination.

7.2 Checklist for Determining the Success of D2 Lymphadenectomy

Scoring Method for D2 Lymph Node Dissection	Complete<br>10	Incomplete<br>5	None<br>0
1. Properly full omentectomy	□	□	□
2. Ligation of the left gastroepiploic artery at origin	□	□	□
3. Ligation of the right gastroepiploic artery at origin	□	□	□
4. Full exposure of the common hepatic artery	□	□	□
5. Ligation of the right gastric artery at origin	□	□	□
6. Exposure of the portal vein	□	□	□
7. Exposure of the splenic artery	□	□	□
8. Identification of the splenic vein	□	□	□
9. Ligation of the left gastric artery at origin	□	□	□
10. Exposure of the gastroesophageal junction	□	□	□

1. Properly full omentectomy
   a. Omentectomy was performed close to the transverse colon
   b. Omentectomy was performed from hepatic flexure to splenic flexure
   c. The anterior layer of the transverse colonic mesentery and anterior pancreatic peritoneum were dissected.
2. Ligation of the left gastroepiploic artery at origin
3. Ligation of the right gastroepiploic artery at origin
4. Full exposure of common hepatic artery
   a. More than half of the anterior part of the common hepatic artery was exposed.
5. Ligation of the right gastric artery at origin
6. Exposure of the portal vein
7. Exposure of the splenic artery
   a. The anterior part in the splenic artery was exposed
   b. The splenic artery was exposed from the celiac trunk to the posterior gastric artery (distal gastrectomy)
   c. The splenic artery was exposed from the celiac trunk to the terminal branch of the splenic artery (total gastrectomy)
8. Identification of the splenic vein
9. Ligation of the left gastric artery at origin
10. Exposure of the gastroesophageal junction

a. The anterior and right side of the abdominal esophagus were exposed (distal gastrectomy)

b. The anterior, posterior, left, and right sides of the abdominal esophagus were exposed (total gastrectomy)

- D2 lymphadenectomy was accepted if all three randomly assigned investigators rated ≥85 points regarding checklists in the unedited video review.

8. End point and definition of related result determination

8.1 Definition of recurrence and recurrence date

The following situations are regarded as "recurrence" and should be recorded as evidence of "recurrence" in the CRF.

(1) Recurrence identified by anyone image examination (X-ray, ultrasound, CT, MRI, PET-CT, endoscope, etc.) and, if there are a variety of imaging examinations, results without contradiction determined "recurrence". The earliest date that the recurrence is found is defined as the "recurrence date".

(2) For cases that lack the use of imaging or a pathological diagnosis, the date we diagnose the occurrence of clinical recurrence based on the clinical history and physical examination is defined as the "recurrence date".

(3) For cases without imaging or clinical diagnosis but with a cytology or tissue biopsy pathological diagnosis of recurrence, the earliest date confirmed by cytology or biopsy pathology is considered the "recurrence date".

(4) A rise in CEA or other associated tumor markers alone could not be diagnosed as a relapse.

8.2 Incidence of intraoperative complications

8.2.1 Incidence of postoperative complications

The number of all patients treated with surgery as the denominator and the number of patients with any intraoperative and postoperative complications as the numerator is used to calculate the proportions.

8.2.2 Incidence of overall postoperative complications

The postoperative complication criteria refer to short-term complications after surgery in the postoperative observation project (see 9.4.5). The time is defined as within 30th after surgery, or the first discharge time if the days of hospital stay more than 30 days.

8.2.3 Incidence of postoperative major complications

The standard for postoperative major complications refers to the short-term complications in the postoperative observation project (see 9.4.5) according to the Clavien–Dindo grade, IIIA level and above for serious complications, and when multiple complications occur simultaneously, the highest-ranked complication is the subject.

8.3 Incidence of operative complications

The number of all patients treated with surgery as the denominator and the number of patients with any intraoperative and postoperative complications as the numerator is used to calculate the proportions. The criteria for the intraoperative complications refer to the descriptions of intraoperative complications in the observation project (in 9.3.3).

8.4 Mortality

- The number of all the patients receiving surgery as the denominator and the number of patients in any of the following situations as the numerator is used to calculate proportions. This proportion indicated the operative mortality ratio.
- Situations: patients whose death was identified according to documented intraoperative observation items, including patients who die within 30 days after the surgery (including 30 days) regardless of the causality between the death and the surgery, and patients who die more than 30 days after the surgery (whose death is proved to have a direct causal relationship with the first operation).

8.5 Disease-free survival

Disease-free survival is calculated from the day of surgery to the day of recurrence or death (When the specific date of recurrence of the tumor is unknown, the endpoint is the date of death due to tumor causes). If neither death nor recurrence of the tumor is observed, the endpoint is the final date that a patient is confirmed as relapse-free. (The final date of DFS: The last date of the outpatient visit day or the date of acceptance of the examination). (Follow-up cycle and required examinations are shown in the follow-up process 9.5.3).

8.6 Overall survival time

The overall survival is calculated from the day of surgery until death or until the final follow-up date, whichever occurs first. For survival cases, the endpoint is the last date that survival was confirmed. If the loss to follow-up occurred, the endpoint is the final date that survival could be confirmed.

8.7 Determination of surgical outcomes

8.7.1 Operative time: from skin incision to the skin being sutured

8.7.2 Postoperative recovery indexes

8.7.2.1 Time to ambulation, flatus, recovery of liquid diet, and semi-liquid diet.

- During the day of surgery to the first discharge, the initial time to ambulation, flatus, liquid diet, and semi-liquid diet during the postoperative hospitalization is recorded by the day.
- Flatus on the operation day should be excluded.
- If flatus or resumption of liquid and semi-liquid diet does not occur before hospital discharge, the discharge time should be recorded as the corresponding time.
- The initial time to ambulation, flatus, liquid diet and semi-liquid diet should be recorded according to patients' reports.

8.7.2.2 The maximum temperature

The highest value of body temperature measured at least 3 times a day from the first day to the eighth day after the operation is documented.

8.7.3 Percentage of conversion to laparotomy

Among all the patients who underwent surgery, the number of patients planning to receive a laparoscopic surgery per-protocol is used as the denominator, while the number of the patients who receive a conversion to open surgery is considered the numerator. The proportion calculated is regarded as the rate of transfer laparotomies. In this study, if the length of the auxiliary incision is more than 10 cm, it is considered a conversion to open surgery.

8.8 Fluorescent station and fluorescent LN

After fluorescent lymphography-guided lymphadenectomy, each LN station was first separated ex vivo from the resected specimen in accordance with the definitions of the Japanese classification of gastric carcinoma.[17] Thereafter, LN stations and LNs were examined for fluorescence directly through near-infrared imaging. LNs were classified as “fluorescent” if they were stained with ICG and “nonfluorescent” if they were not stained with ICG. Stations containing fluorescent LNs were classified as “fluorescent” stations, while those without fluorescent LNs were classified as “non-fluorescent” stations.

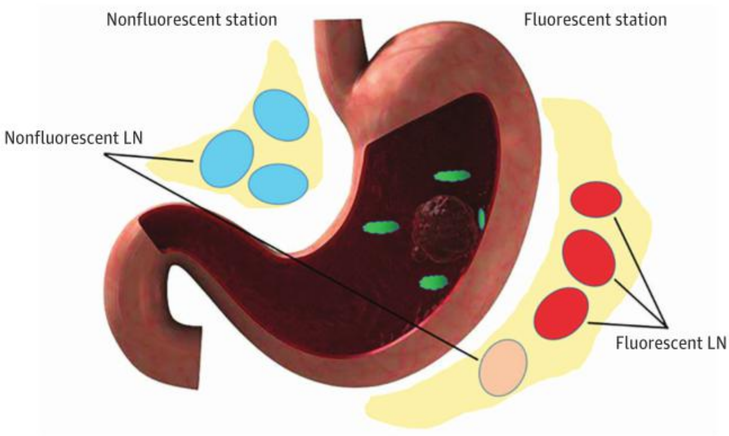

8.9 Diagnostic test

The presence and absence of fluorescent staining were subsequently matched with the results of histopathological analysis. True-positive (TP) stations were defined as fluorescent stations observed through near-infrared imaging, which contained metastatic LNs on histopathological analysis. False-negative (FN) stations were defined as non-fluorescent stations observed through near-infrared imaging, which contained metastatic LNs on histopathological examination. True-positive LNs were defined as fluorescent LNs observed through near-infrared imaging, which were metastatic on histopathological assessment. False-positive (FP) LNs were defined as fluorescent LNs observed through near-infrared imaging, which were not metastatic on histopathological assessment. False-negative LNs were defined as non-fluorescent LNs

observed through near-infrared imaging, which were metastatic on histopathological examination.

The sensitivity, specificity, false-negative rate, negative predictive value (NPV), and positive predictive value (PPV) of fluorescent lymphography for detecting LN metastases based on stations and LNs were evaluated. These proportions were determined by matching the presence or absence of fluorescence staining in stations or LNs with the results of histopathological analysis. First, the sensitivity of fluorescent lymphography was determined from the total number of patients with LN metastases. Therefore, the diagnostic value of ICG fluorescent lymphography was determined on the basis of the total number of stations and LNs. Sensitivity (TP rate) was determined by dividing the number of TP stations or LNs by the number of TP plus FN stations or LNs [TP / (TP + FN)]. Specificity (true-negative rate) was determined by dividing the number of TN stations or LNs by the number of TN plus FP stations or LNs [TN / (TN + FP)]. The FP rate of fluorescent stations or LNs was determined by dividing the number of FP stations or LNs by the number of FP plus true-negative (TN) stations or LNs [FP / (FP + TN)]. The FN rate of fluorescent stations or LNs was determined by dividing the number of FN stations or LNs by the number of FP plus TP stations or LNs [FN / (FN + TP)]. PPV was determined by dividing the number of TP stations or LNs by the number of TP plus FP stations or LNs [TP / TP + FP]. NPV was determined by dividing the number of TN stations or LNs by the number of TN plus FN stations or LNs [TN / (TN + FN)].

8.10 The Surgery Task Load Index

Surgeons were asked to complete one Surg-TLX questionnaire for each procedure in both studies after surgery[18], consisting of 6 subscales addressing mental, physical, and temporal demands, task complexity, situation, and distrations. All questions were rated on a 20-point scale (0 = low, 20 = high).

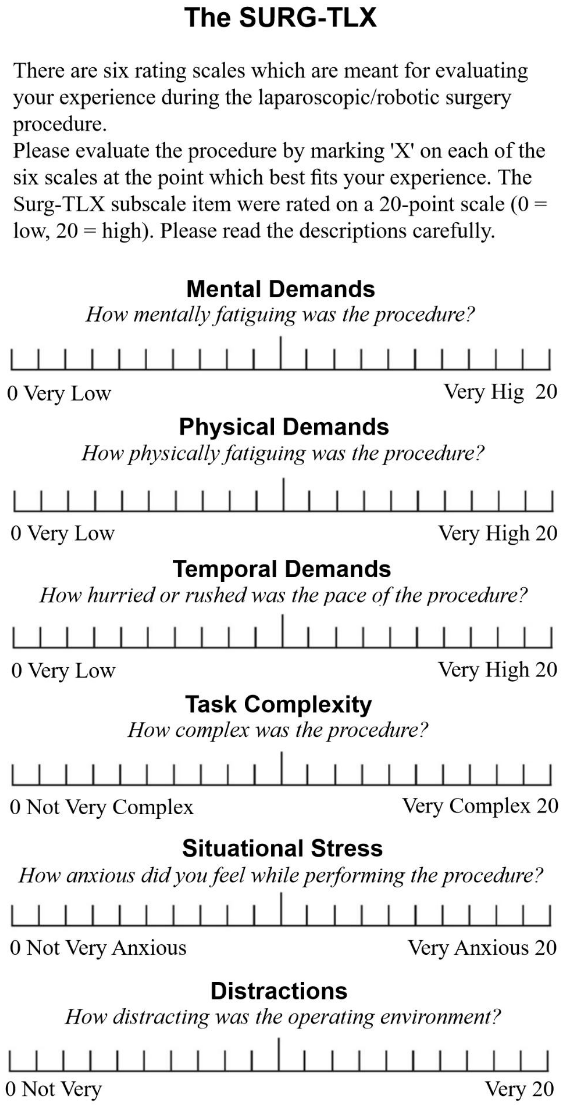

The SURG-TLX

There are six rating scales which are meant for evaluating your experience during the laparoscopic/robotic surgery procedure.
Please evaluate the procedure by marking 'X' on each of the six scales at the point which best fits your experience. The Surg-TLX subscale item were rated on a 20-point scale (0 = low, 20 = high). Please read the descriptions carefully.

Mental Demands
How mentally fatiguing was the procedure?
0 Very Low — Very Hig 20

Physical Demands
How physically fatiguing was the procedure?
0 Very Low — Very High 20

Temporal Demands
How hurried or rushed was the pace of the procedure?
0 Very Low — Very High 20

Task Complexity
How complex was the procedure?
0 Not Very Complex — Very Complex 20

Situational Stress
How anxious did you feel while performing the procedure?
0 Not Very Anxious — Very Anxious 20

Distractions
How distracting was the operating environment?
0 Not Very — Very 20

8.11 LN noncompliance rate

Within the scope of D2 dissection, LN noncompliance was defined as the absence of LNs that should have been excised from more than 1 LN station. Major LN noncompliance was defined as more than 2 intended LN stations that were not removed.

8.12 Patient satisfaction with care

Patient satisfaction with care was measured before their discharge from hospital by the Modified EORTC IN-PATSAT14 scale. The Modified EORTC IN-PATSAT14 was conceptualized as containing five multi-item and three single-item scales.[19] All scores are linearly transformed to a 0–100 scale. A higher score reflects a higher level of satisfaction.

The modified EORTC IN-PATSAT14 Questionnaire

We are interested in your experience of the care received during your hospital stay. Please answer all the questions yourself by circling the number that best applies to you. There are no "right" or "wrong" answers. The information that you provide will remain strictly confidential. Thank you very much.

	During your hospital stay, how would you rate **doctors**, in terms of	Poor	Fair	Good	Very good	Excellent
1	Their knowledge and experience of your illness?	1	2	3	4	5
2	The treatment and medical follow-up they provided?	1	2	3	4	5
3	Their willingness to listen to all of your concerns?	1	2	3	4	5
4	The information they gave you about your illness, medical tests, and treatment?	1	2	3	4	5
5	The frequency of their visits/consultations?	1	2	3	4	5
6	The time they devoted to you during visits/consultations?	1	2	3	4	5
	During your hospital stay, how would you rate **services** in terms of	Poor	Fair	Good	Very good	Excellent
7	The information provided on your admission to the hospital?	1	2	3	4	5
8	The information provided on your discharge from the hospital?	1	2	3	4	5
9	The kindness and helpfulness of the technical, reception, laboratory personnel?	1	2	3	4	5
10	The waiting time for obtaining results of medical tests?	1	2	3	4	5
11	The speed of implementing medical tests and/or treatments?	1	2	3	4	5
	**Special item**	Quite a lot	Serious	Moderate	Middle	Not at all
12	Was any discomfort caused by the examination or treatment during the hospitalization period?	1	2	3	4	5
13	Is there any examination or treatment that you think is repeated and bother you?	1	2	3	4	5
	**Overall**	Poor	Fair	Good	Very good	Excellent
14	How would you rate the care received during your hospital stay?	1	2	3	4	5

The modified EORTC IN-PATSAT14 contains five multi-item and three single-item scales. These include the doctors' technical skills (items 1 and 2), interpersonal skills (item 3), information provision (item 4), availability (items 5 and 6) scales; other hospital staff interpersonal skills and information provision scale (items 7–9); waiting time scale (items 10 and 11); comfort special-item scale (items 12 and 13); and general satisfaction single-item scale (item 14).

9 Standard operating procedures (SOP)

9.1 Case selection

9.1.1 Selection assessment items

Clinical examination data of patients conducted from hospital admission to enrollment into this study (time period is usually 2 week) will be considered baseline data, and must include:

(1) Systemic status: ECOG PS score, height, weight

(2) Peripheral venous blood: Hb、RBC、WBC、LYM、NEU、NEU%、PLT、

MONO

(3) Blood biochemistry: albumin, total bilirubin, indirect bilirubin, direct bilirubin, AST, ALT, creatinine, urea nitrogen, total cholesterol, triglycerides, fasting glucose, potassium, sodium, chlorine, calcium

(4) Serum tumor markers: CEA、CA19-9、CA72-4、CA12-5、AFP

(5) Full abdominal (slice thickness of 10mm or less, in case of allergy to the contrast agent, CT horizontal scanning is allowed only)

(6) Upper gastrointestinal endoscopic ultrasonography (EUS) and biopsy, if no EUS, select ordinary upper gastrointestinal endoscopy and biopsy instead

(7) Chest X-ray (AP and lateral views): cardiopulmonary conditions

(8) Resting 12-lead ECG

(9) Respiratory function tests: FEV1, FVC

9.1.2 Selection application

For cases that meet all inclusion criteria and none of the exclusion criteria, talk to patients and their families, and sign informed consent. Application and confirmation of eligibility should be completed preoperatively; postoperative applications will not be accepted.

9.2 Preoperative management

After the eligibility is obtained, surgery should be performed within two weeks (including the 14th day)

- In case of any deterioration of the clinical conditions from the selected time to the expected day of surgery, whether to undergo an elective surgery as planned should be decided under the judgment of the doctor in charge; if emergency surgery is required, the case should be withdrawn from PP set according to 4.3 Withdrawal Criteria;
- For patients with nutritional risks, preoperative enteral/parenteral nutritional support is allowed.
- For elderly, smokers, high-risk patients with diabetes, obesity, and chronic cardiovascular/cerebrovascular or thromboembolic history, among others, perioperative low-molecular-weight heparin prophylaxis, lower-limb antithrombotic massage, active lower limb massage, training in respiratory function, and other preventive measures are recommended. For other potentially high-risk complications not specified in this study protocol, the doctor in charge of the research center can decide on the most appropriate approach according to clinical practice and should record it in the CRF.
- For the operative approach of the surgeries in this study should be selected by the doctor in charge according to his/her experience and the specific intraoperative circumstances.
- Preoperative fasting and water deprivation and other before-anesthesia

requirements on patients should follow the conventional anesthesia program of the research center, which is not specified in this study.

- For prophylactic antibiotics, the first intravenous infusion should begin 30 minutes before surgery. It is recommended to select a second-generation cephalosporin (there are no provisions on specific brands in this study); the preparation, concentration, and infusion rate should comply with routine practice; and prophylaxis should not exceed postoperative three days at a frequency of one infusion every 12 hours. If the patient is allergic to cephalosporins (including a history of allergy or allergy after cephalosporin administration), other types of antibiotics are allowed according to the specific clinical situation and when used over the same period mentioned.
- Patient data to be collected during the preoperative period also includes CRP
  - For patients who were assigned to the SSA group, after preoperative exploration, the indocyanine green powder (Dandong Yichuang Pharmaceutical Co) is dissolved in 0.5 mg/ml of sterile water and the prepared solution (1.5 ml for each point) is injected along the subserosal of the stomach at 6 specific points along the lesser and greater curvature of the stomach. If the tumor invades one or more of the six injection points, subserosal injection of ICG will be conducted at the tumor non-invasive sites along the greater or lesser curvature of the stomach next to the established injection point.

A Distal gastrectomy　　**B** Total gastrectomy

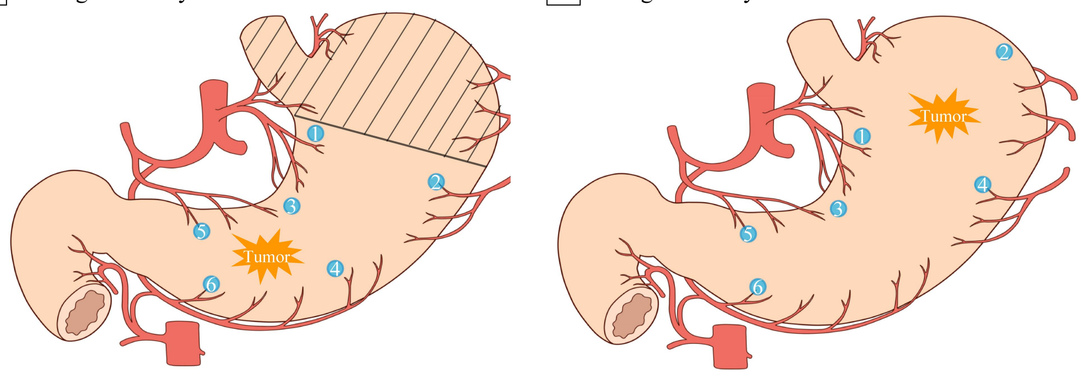

A:
① between the 1st and the 2nd gastric wall branches of the left gastric artery
② the 1st gastric wall branch of the left gastroepiploic artery
③ the gastric angular notch
④ the midpoint of two injection points (② and ⑥) in the greater curvature
⑤ the 1st gastric wall branch of the right gastric artery
⑥ the 1st gastric wall branch of the right gastroepiploic artery

B:
① between the 1st and the 2nd gastric wall branches of the left gastric artery
② the junction area between fundus and body in the greater curvature
③ the gastric angular notch
④ the 1st gastric wall branch of the left gastroepiploic artery
⑤ the 1st gastric wall branch of the right gastric artery
⑥ the 1st gastric wall branch of the right gastroepiploic artery

- For patients who were assigned to the SMA group, submucosal injection of indocyanine green One day before surgery, 1.25 mg/ml indocyanine green (Dandong Yichuang Pharmaceutical Co) was prepared in sterile water and 0.5 ml of the solution was injected into the submucosal layer at 4 quadrants around the primary tumor, amounting to 2.5 mg of indocyanine green.

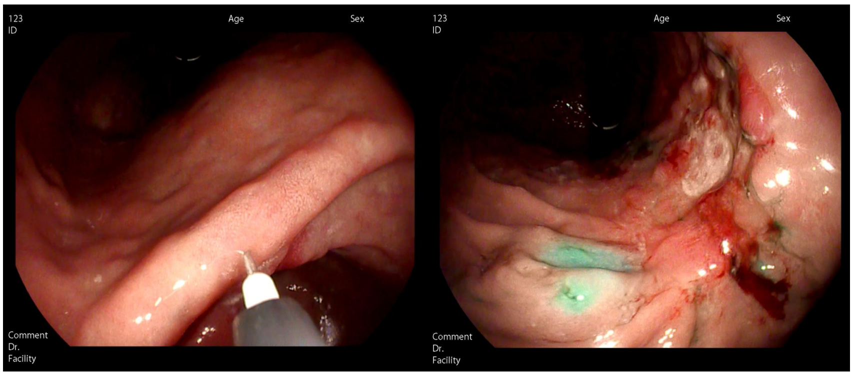

Figure 1. Endoscopic submucosal injection of ICG one day before surgery.

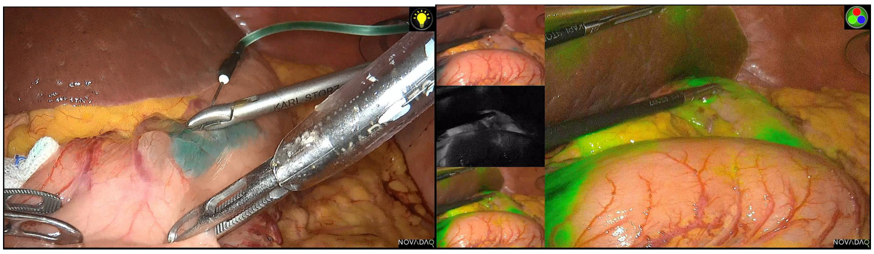

Figure 2. Laparoscopic subserosal injection of ICG at the begning of surgery

9.3 Standardization of surgical practice

9.3.1 Handling practices followed by both groups

9.3.1.1 Anesthesia

The operation is to be carried out with endotracheal intubation under general anesthesia; whether epidural assisted anesthesia is applied or not is left at the discretion of the anesthetist and is not specified in this study protocol.

9.3.1.2 Intraoperative exploration

Explore the abdominal cavity for any hepatic, peritoneal, mesenteric, or pelvic metastases and gastric serosal invasion

9.3.1.3 Regulations on the extent of the gastrectomy

If the oncological principles first can be satisfied, it is determined by the surgeon according to his experience and the specific circumstances of the operation.

9.3.1.4 Regulations on digestive tract reconstruction

The digestive tract reconstruction method is to be determined by the surgeon according to his/her own experience and the intraoperative situation. If instrumental anastomosis is used, whether the manual reinforced stitching is to be performed or not on anastomotic stoma is determined by the surgeon and not specified in this study protocol.

9.3.1.5 Regulations on surgery-related equipment and instruments

We used the NOVADAQ Fluorescence Surgical System (Stryker, US) equipped with the fluorescence mode to acquire NIR fluorescent images. A simple finger click can change a visible light into NIR fluorescent images (NIR imaging, green fluorescence, and color-segmented fluorescence) without the need to change any

equipment, because the surgical system contains a module for fluorescence imaging, the surgeon could turn on the NIR fluorescent mode during the LN dissection.

Energy equipment, vascular ligation method, digestive tract cutting closure, and digestive tract reconstruction instruments are determined by the surgeon in charge of the operation according to his/her own experience and actual needs and are not specified in this study protocol.

9.3.1.6 Regulations on ICG-guide LN dissection

Sequences of LN dissection were routinely performed as follow[20,21]: (1) for total gastrectomy: No. 6 → No.7, 9, 11p → No. 8a, 12a, 5 → No. 1 → No. 4sb → No. 4sa, 11d → No. 2; and for (2) distal gastrectomy: No. 6 → No. 7, 9, 11p → No. 3, 1 → No. 8a, 12a, 5 → No. 4sb.

No.10 LNs were performed a selective dissection when the primary tumor was located in the upper-middle part of the stomach and invading the greater curvature or preoperative imaging suggests splenic LN enlargement or No.10 LNs emitted fluorescence under the NIR mode.[22-25]

For patients in the ICG group, after finished the all LNs dissection, routine imaging of the surgical area was performed to determine whether there is residual fluorescent LN. When residual LNs containing fluorescence were detected in the dissected area, we performed complementary dissection of these LNs. Also, if fluorescent LNs were detected outside the planned dissection area (No. 10, and 14v), excessive dissection beyond the scope of D2 LN dissection was performed.

9.3.1.6 Regulations on the gastric canal and peritoneal drainage tube

Whether an indwelling gastric canal or peritoneal drainage tube is left or not after the operation is determined by the surgeon according to his/her own experience and actual needs and are not specified in this study protocol.

9.3.1.7 Regulations on simultaneous surgery for other diseases

If any other system/organ disease is found during surgery, the responsible surgeon and the consultants of relevant departments should jointly determine the performance of a concurrent operation if there is such necessity. The priority of operations is determined according to clinical routine; the patients meeting Exclusion Criteria will be excluded from the PP Set.

9.3.1.8 Regulations on the handling of excluded patients as identified intraoperatively

If the surgeon in charge judges and determines that the patient undergoing surgery belongs to the exclusion case group, then the research approach is suspended and the

surgeon will follow the routine clinical practice to decide subsequent treatment (therapeutic decisions as to whether to excise gastric primary focus and metastases are made by the surgeon in charge); whether to proceed with laparoscopic surgery or convert it to laparotomy will be determined by the surgeon in charge. The excluded cases still need to complete data collection and follow-up and included in the analysis study (ITTP population).

9.3.1.9 Regulations on imagery/photographing

A laparoscopic/digital camera (8 million pixels at least) will be used to take pictures should contain the following contents (see the example below):

(1) Field of LN dissection (15 or 17 pictures)

Inferior pylorus region (2 picture): The right gastroepiploic arteriovenous cut site should be included. Another screenshot in NIR fluorescent mode should be saved.

Right-side area of the superior margin of the pancreas (2 picture): The front top of the entire common hepatic artery, the half front of the inferior proper hepatic artery, and the cut site of the right gastric artery should be included. Another screenshot in NIR fluorescent mode should be saved.

Left-side region of the superior margin of the pancreas (2 picture): The left gastric arteriovenous cut position, celiac arterial trunk, and proximal splenic artery should be included. Another screenshot in NIR fluorescent mode should be saved.

The right side of the cardia and lesser gastric curvature side (2 picture). Another screenshot in NIR fluorescent mode should be saved.

Left gastroepiploic vessel dividing position (2 picture): The cut site of the left gastroepiploic artery and vein should be included. Another screenshot in NIR fluorescent mode should be saved.

Splenic hilus region (2 picture, if applicable); the cut sites of the distal splenic artery and the short gastric vessel should be included. Another screenshot in NIR fluorescent mode should be saved.

(2) After the skin incision is closed (1 picture, measuring scale serving as a reference object).

(3) Postoperative fresh specimens (4 pictures, measuring scale serving as a reference object); 1 picture before and 3 pictures after dissection (mark focus size; 1 picture each of distal and proximal incisional margins). After the specimen is cut open along the greater gastric curvature, a measuring scale is placed as a reference object before taking pictures to record the following items: the distance between the tumor edge and the

proximal incisional margin (1 picture), the distance between the tumor edge and the distal incisional margin (1 picture), and the focus size and appearance of the mucosal face after the specimen is unfolded (1 picture).

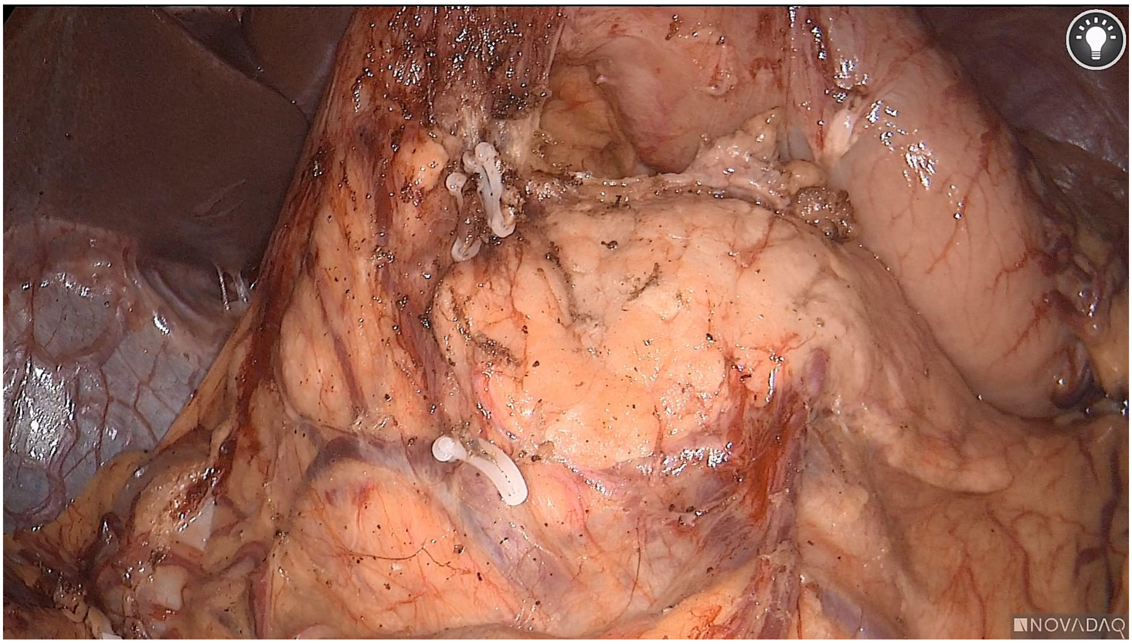

Figure 3-1-1. Inferior pyloric area (no. 6 LNs) under a natural light mode

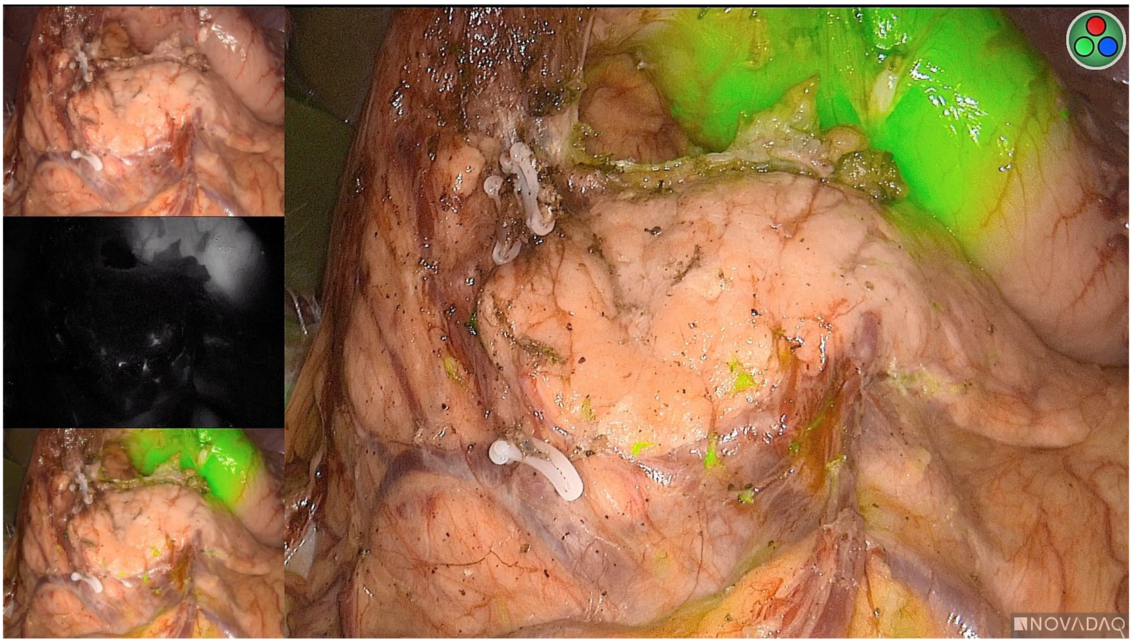

Figure 3-1-2. Inferior pyloric area (no. 6 LNs) under a NIR fluorescent light mode

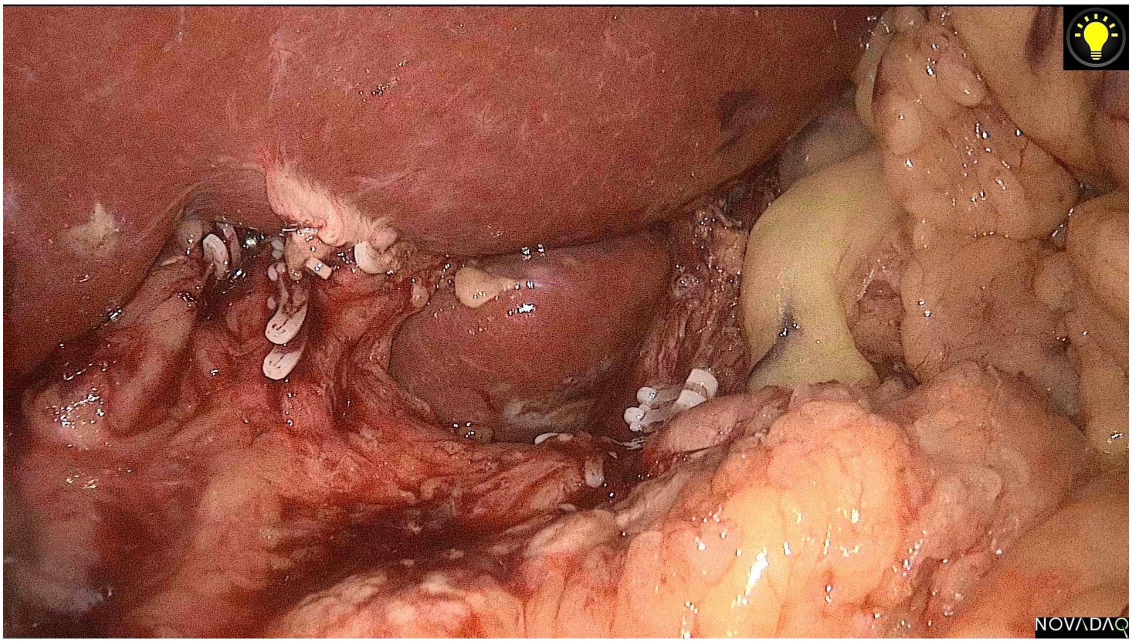

Figure 3-2-1. Right-side area of the superior margin of the pancreas (no. 5, no. 8a, and no. 12a LNs) under a natural light mode

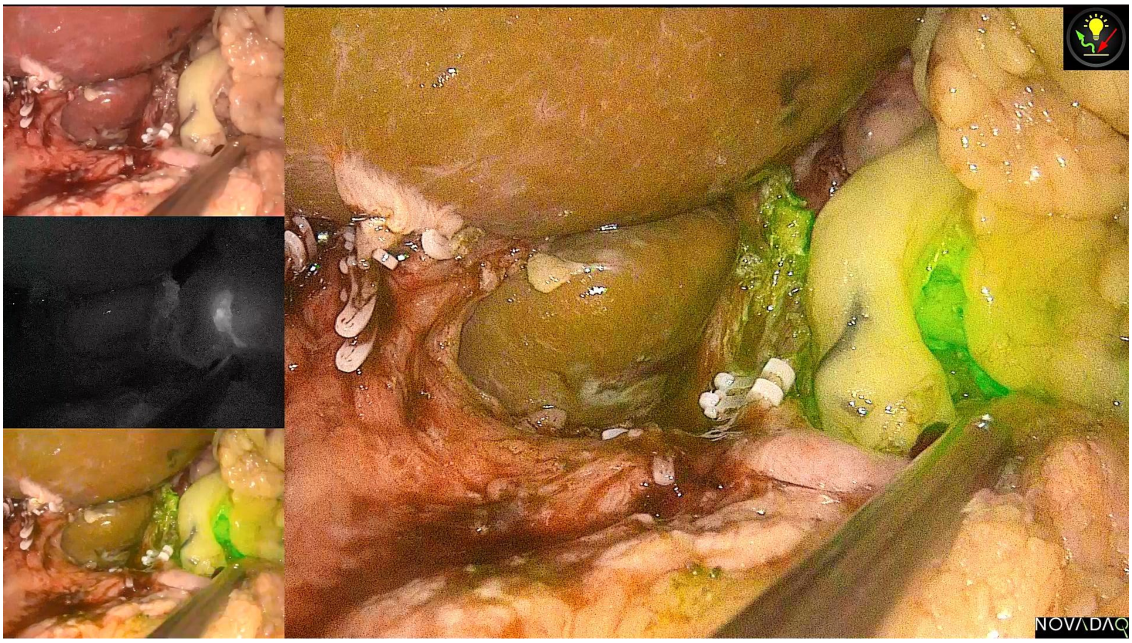

Figure 3-2-2. Right-side area of the superior margin of the pancreas (no. 5, no. 8a, and no. 12a LNs) under a NIR fluorescent light mode

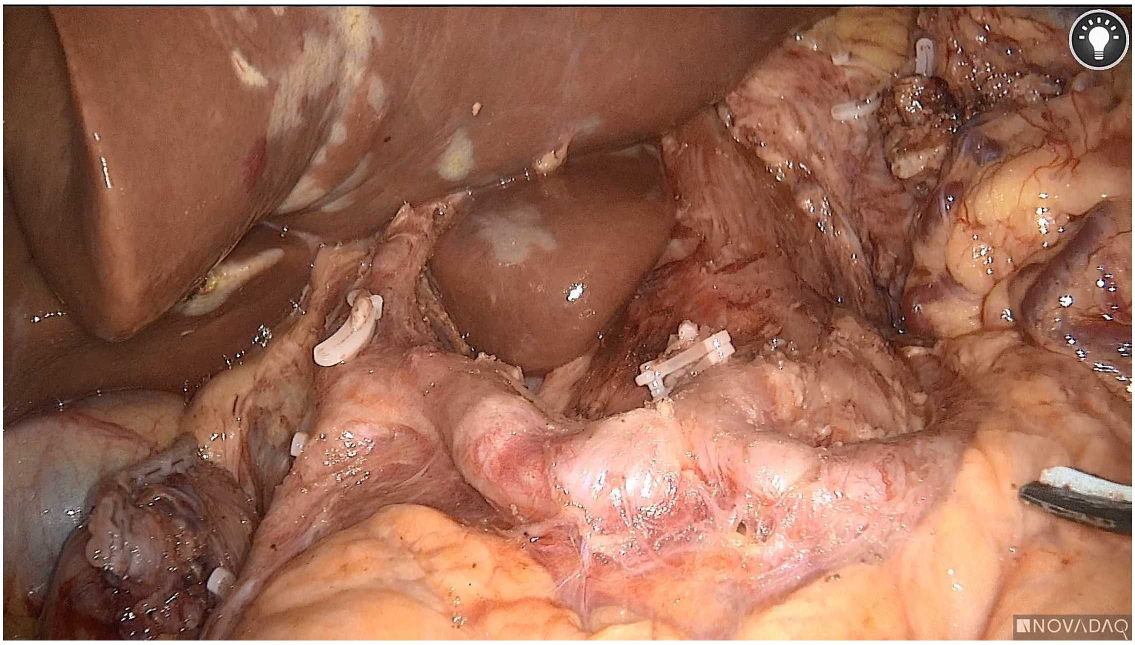

Figure 3-3-1. Left-side area of the superior margin of the pancreas (no. 7, no. 9, and no. 11p LNs) under a nature light mode

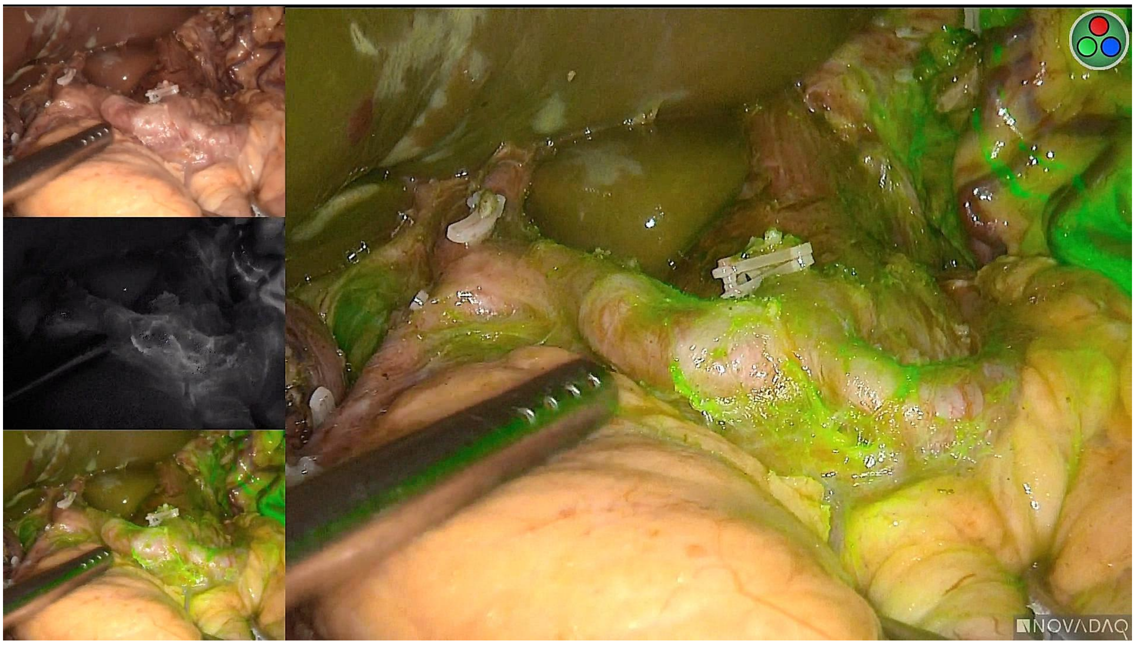

Figure 3-3-2. Left-side area of the superior margin of the pancreas (no. 7, no. 9, and no. 11p LNs) under a NIR fluorescent light mode

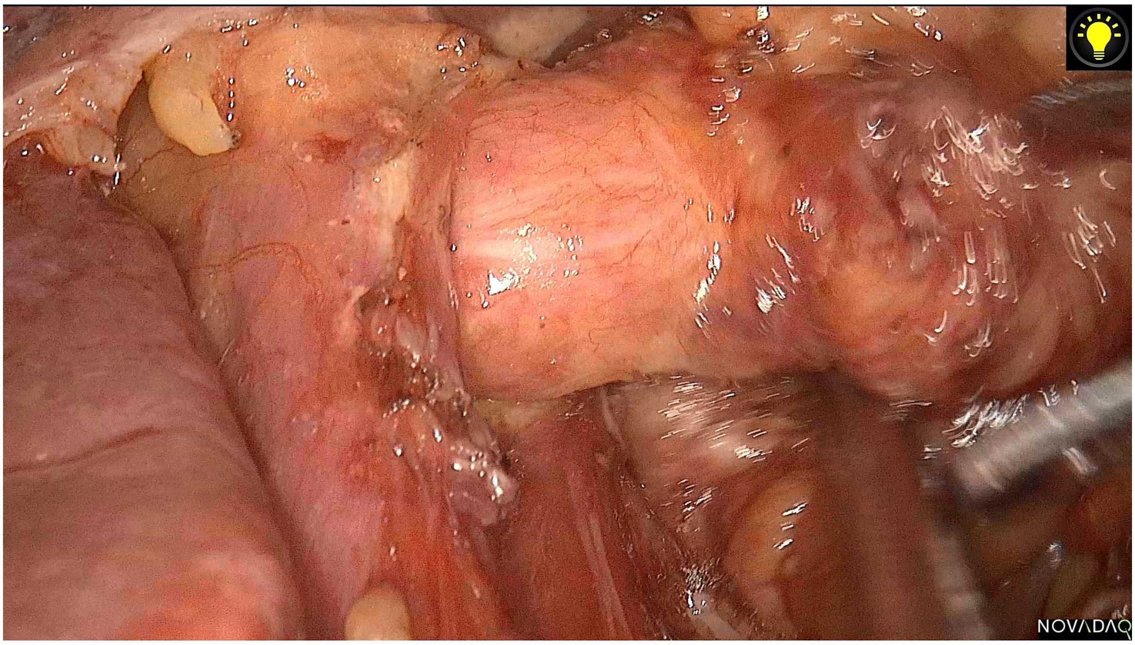

Figure 3-4-1. Right side of the cardia and lesser gastric curvature side (the no. 1 and no. 3 LNs) under a nature light mode

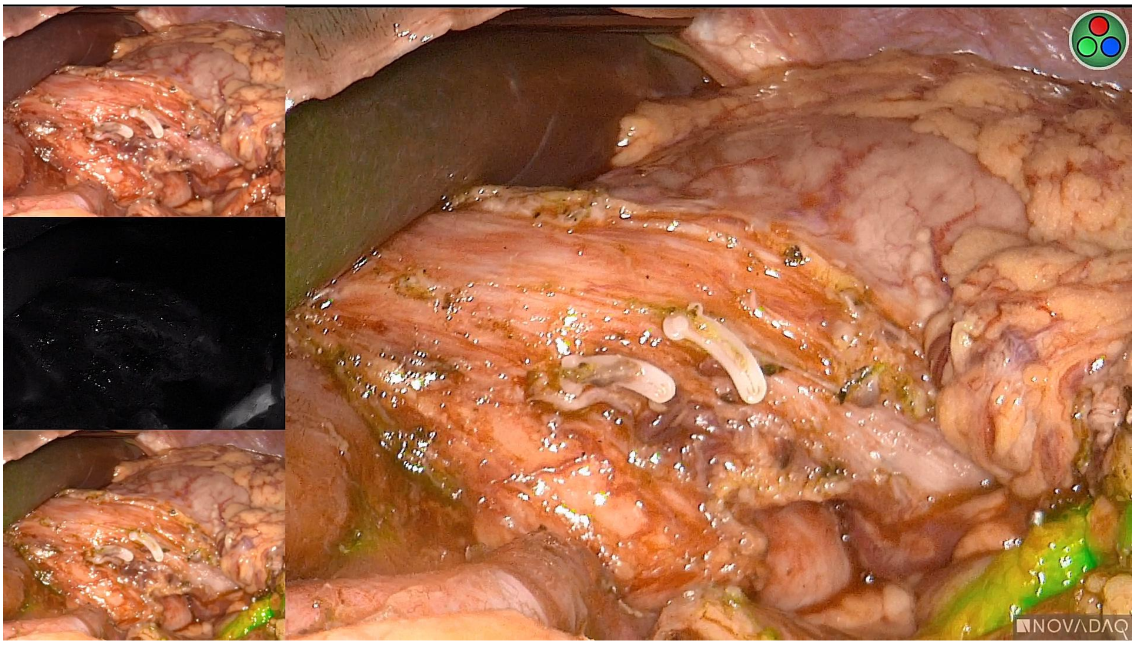

Figure 3-4-2. Right side of the cardia and lesser gastric curvature side (the no. 1 and no. 3 LNs) under a NIR fluorescent light mode

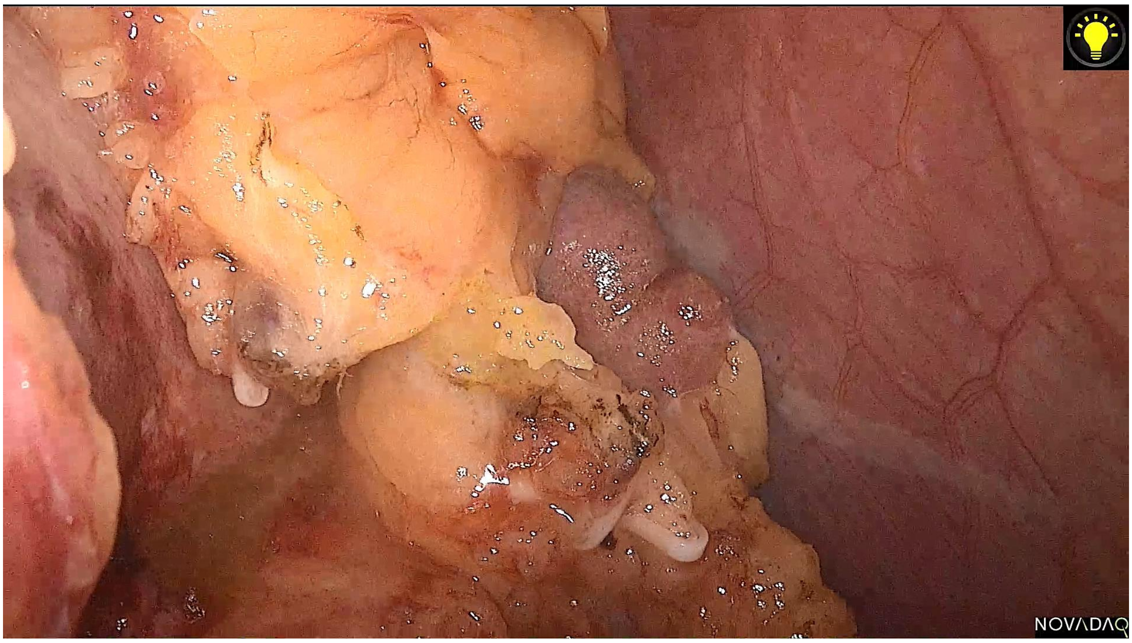

Figure 3-5-1. Cut site of the left gastroepiploic vessel (no. 4 sb LNs) under a nature light mode

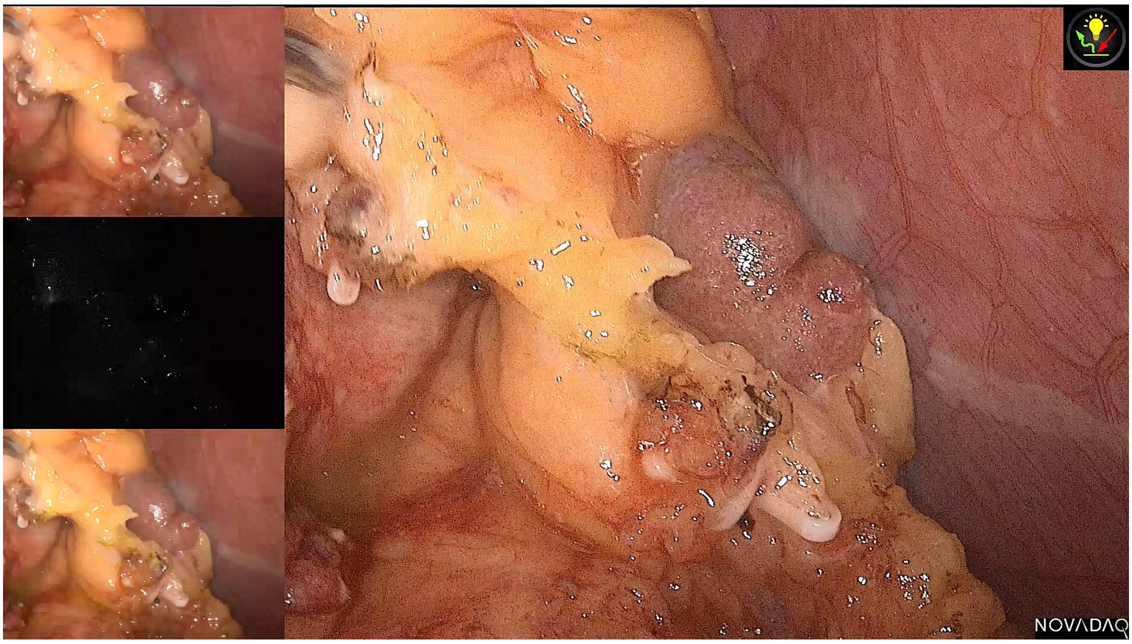

Figure 3-5-2. Cut site of the left gastroepiploic vessel (no. 4 sb LNs) under a NIR fluorescent light mode

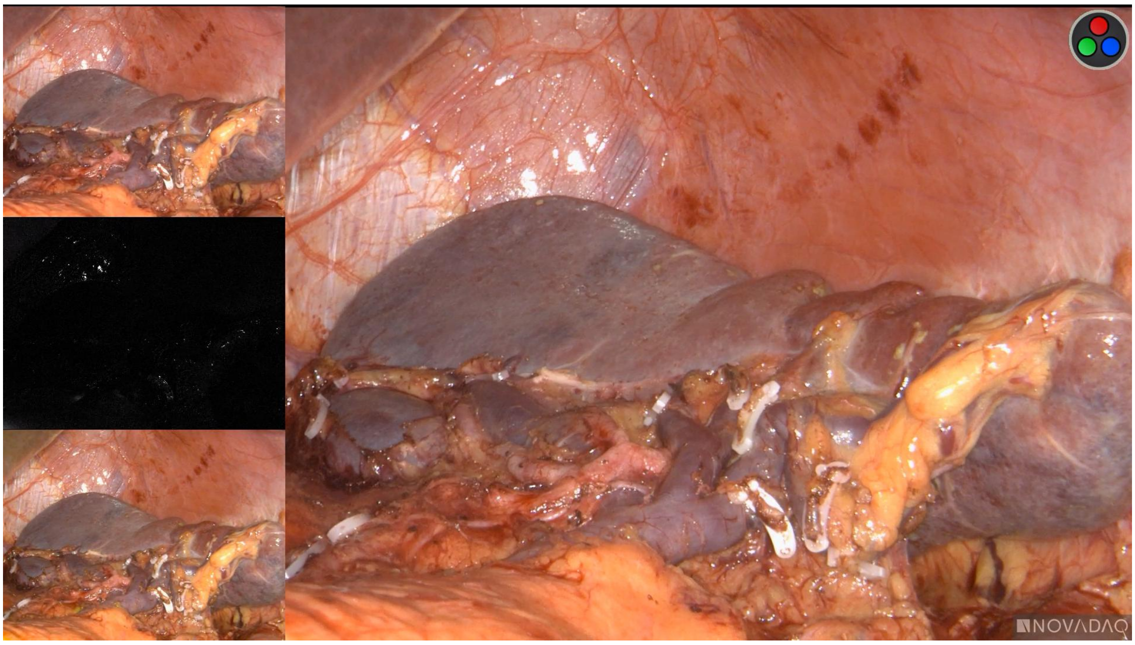

Figure 3-6-1. Splenic hilus area (no. 11d and no. 10 LNs) under a nature light mode

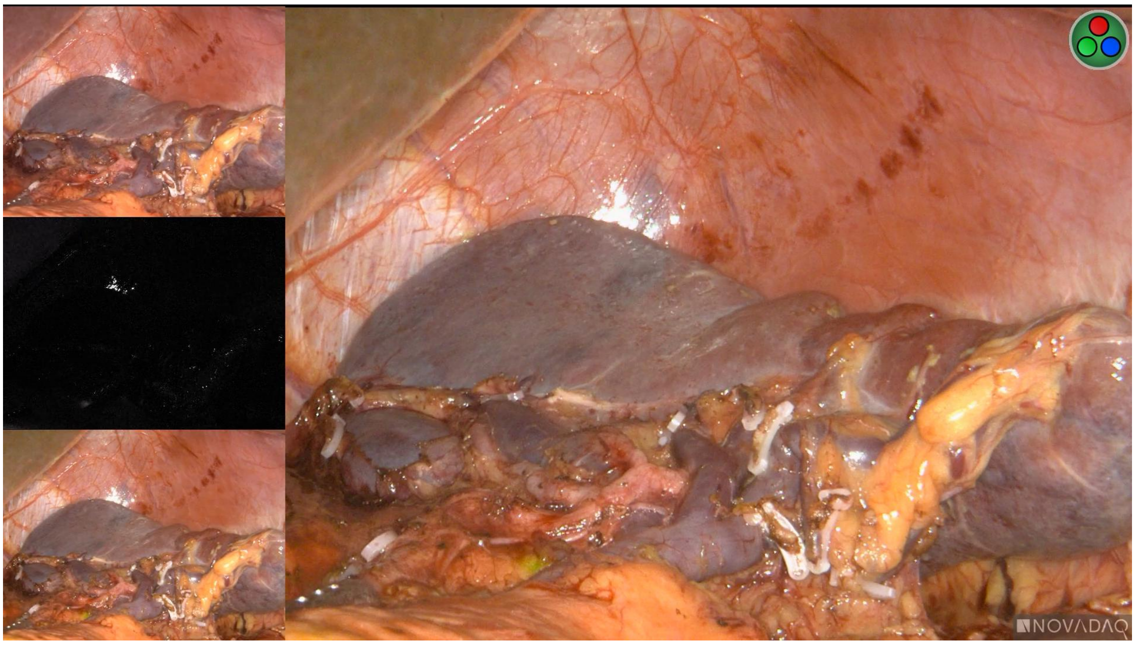

Figure 3-6-2. Splenic hilus area (no. 11d and no. 10 LNs) under a NIR fluorescent light mode

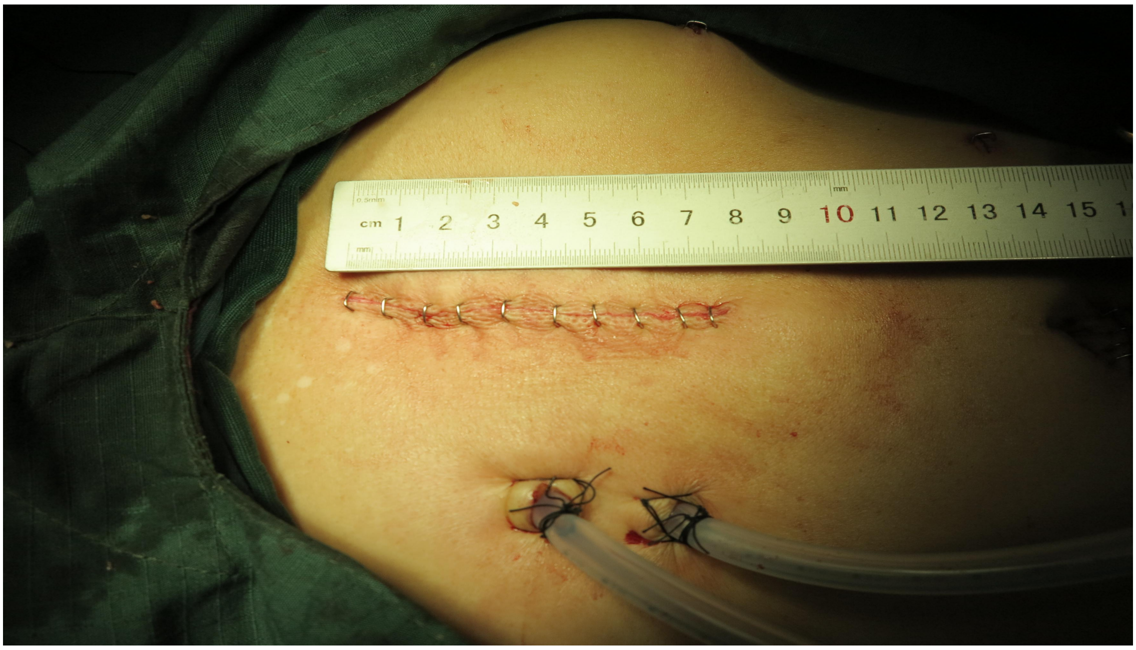

Figure 3-7. Incision appearance (mark the incision length)

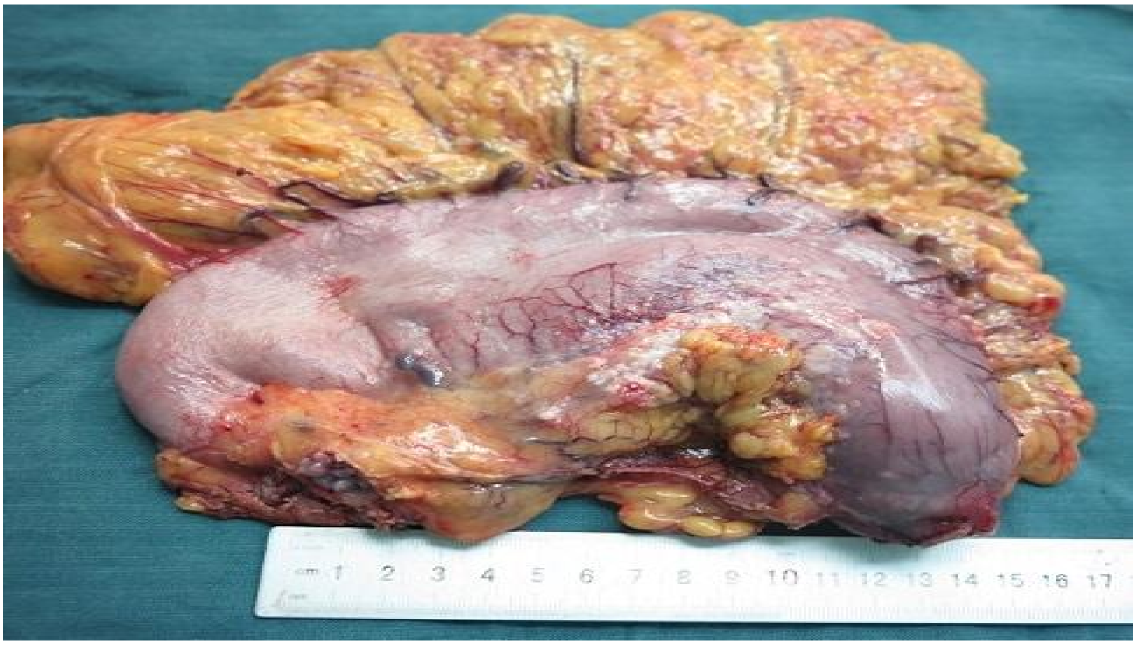

Figure 3-8. Specimen observation (before dissection)

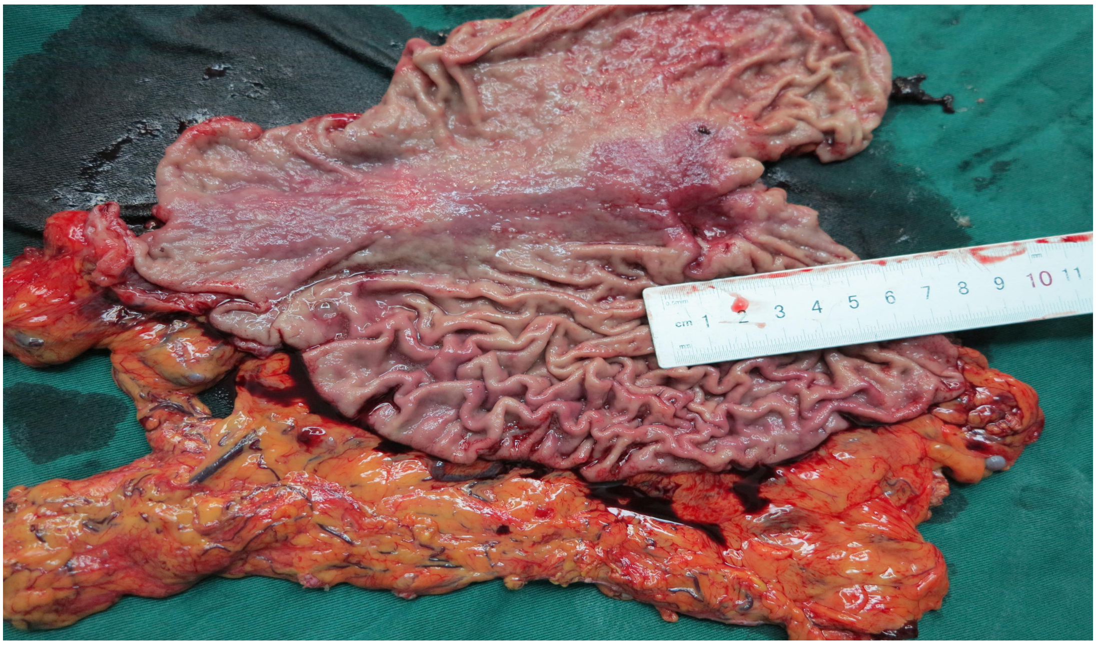

Figure 3-9. Specimen observation (focus size; the dissection is made along the greater gastric curvature, and the focus and incisional margin on the mucosal face are observed; if the tumor is located at the greater gastric curvature, then the dissection is made along the lesser curvature)

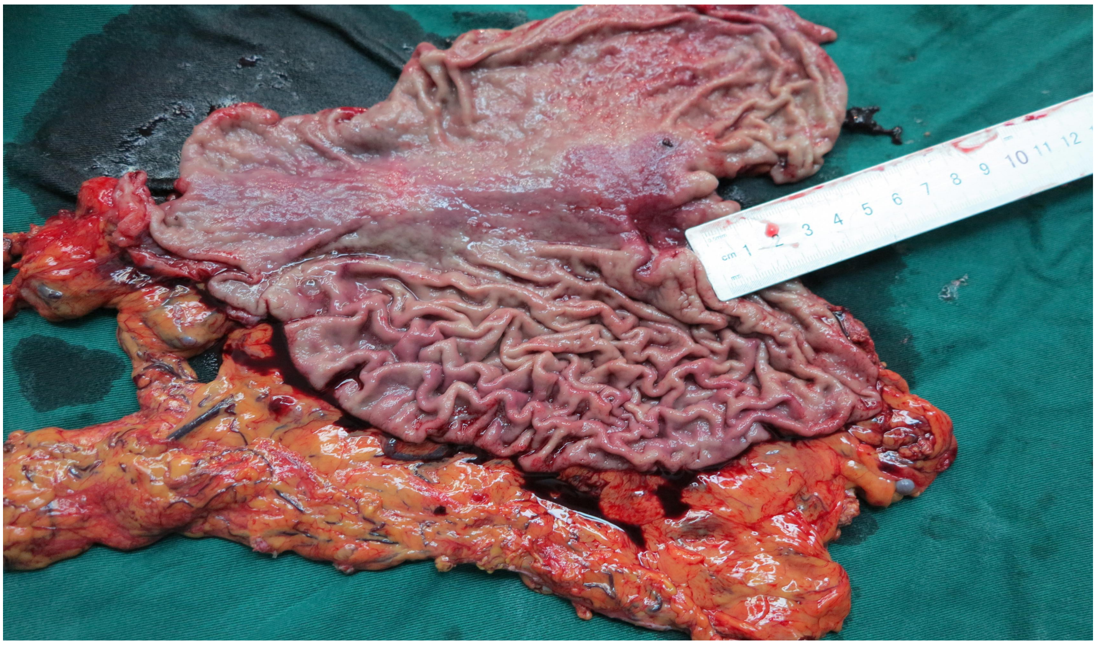

Figure 3-10. Specimen observation (the distance between the tumor edge and the proximal incisional
margin)

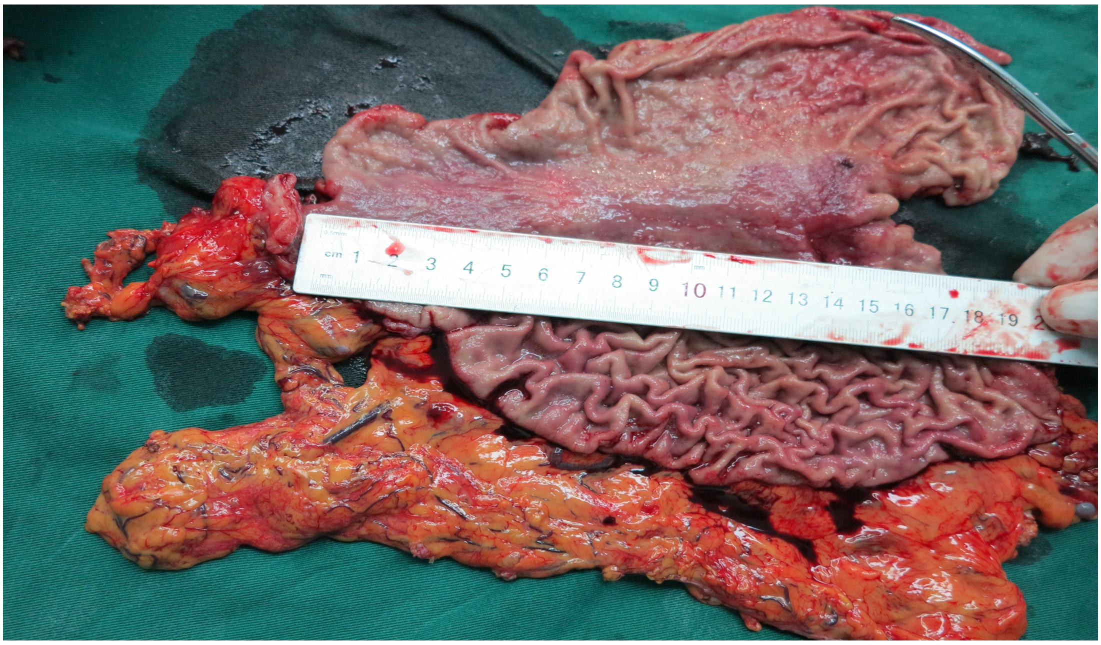

Figure 3-11. Specimen observation (the distance between the tumor edge and the distal incisional margin)

9.3.1.10 Regulations on the photo/ image privacy protection and naming

No image data shall disclose the personal information of patients.

When the photos/images are viewed or reviewed, the personal information must be processed with mosaics or be covered.

The photographed parts should be marked with unified Chinese name: inferior pylorus area (nature light/fluorescent light); left gastroepiploic vessel cut site (nature light/fluorescent light); right-side area of the superior margin of the pancreas (nature light/fluorescent light); left-side area of the superior margin of the pancreas (nature light/fluorescent light); right side of the cardia and lesser gastric curvature side (nature light/fluorescent light); splenic hilus area (nature light/fluorescent light); incision appearance; specimen observation (before dissection); specimen observation (focus size); specimen observation (the distance between the tumor edge and the proximal incisional margin); and specimen observation (the distance between the tumor edge and the distal incisional margin).

For example:

Photo Name: [SMA-subject's random number - Inferior pylorus area]/ [SMA-subject's random number - Inferior pylorus area]

Folder name: [SSA-subject's random number]/ [SSA-subject's random number]

9.3.1.11 Criteria for confirming operation quality

To confirm the appropriateness of the surgical procedure, surgery quality, (auxiliary) incision length and specimen integrity will be assessed in the photographs saved (as stated above) The whole laparoscopic surgery procedure will be videotaped,

and the unclipped image files will be saved.

9.3.1.12 Saving of imaging data

All photographs and data will be saved in the hard disk or portable digital carrier in digital form, and the surgical video required a specific hard drive to be saved for at least 3 years.

If failure to provide the complete photo according to "Regulations on imagery/photographing" is confirmed, the Research Committee will judge and record the surgery quality as unqualified; however, the case will remain in the PP set data of this study.

9.3.2 Regulations on laparoscopy

9.3.2.1 Regulations on pneumoperitoneum

Carbon dioxide pneumoperitoneum will be used to maintain the pressure at 12-14 mmHg.

9.3.2.2 Regulations on punctures and auxiliary incision

The positions of punctures and auxiliary small incision are not specified; the number of punctures should not exceed 5. There should be only one auxiliary small incision whose length shall not exceed the maximum tumor diameter and necessarily will be less than 10 cm in normal cases. If the auxiliary small incision needs to be longer than 10 cm, the surgeon in charge should make a decision and record the reasons in the CRF.

9.3.2.3 Definition of laparoscopic approach

The operations within the abdominal cavity must be performed using laparoscopic instruments with the support of a camera system. Perigastric disassociation, greater omental excision, omental bursa excision, LN dissection, and blood vessel handling should be completed under laparoscopic guidance. For gastrectomy and digestive tract reconstruction, the use of auxiliary small incisions is allowed and can be completed with an open abdomen.

9.3.2.4 Regulations on conversion to laparotomy

When intra-abdominal hemorrhage, organ damage and other serious/life-threatening complications that are difficult to control occur during laparoscopic surgery, it is necessary to actively convert to laparotomy. If the anesthesiologist and surgeon consider that intraoperative complications caused by carbon dioxide pneumoperitoneum may threaten the patient's life, it is necessary to actively convert to open surgery. The surgeon in charge can decide to convert to laparotomy driven by other technical or equipment reasons and will record said reasons. The reasons for conversion to open surgery must be clearly recorded in the CRF. The incision length of > 10 cm is defined as a case of conversion to open surgery in this study.

9.3.2.5 Subsequent treatment of excluded patients from the laparoscopic surgery

Whether the patients continue to undergo surgery under laparoscopy or are converted to open surgery should be at the surgeon's discretion according to his or her clinical experience.

9.3.3 Operative parameters (same for both groups)

Completed by the research assistant on the day of the operation. specific projects include:

(1) Name of responsible surgeons

(2) Operation time (min)

(3) Type of operation, digestive tract reconstruction, intraoperative damage and whether the tumor was ruptured during surgery (intact rupture of the capsule)

(4) Length of incision (cm)

(5) Conversion to open surgery or not and the reasons for this decision

(6) Intraoperative estimated blood loss (ml; from skin cutting to stitching, intraoperative blood loss = (postoperative gauze weight, grams - preoperative gauze weight, grams) *1ml/g+ suction fluid, ml)

(7) Blood transfusion (ml): in this study, the blood transfusion event is defined as transfusion of red cell suspension (ml) or whole blood (ml)

(8) Tumor location

(9) Tumor size (maximum tumor diameter, mm)

(10) Distant metastasis (location)

(11) Proximal resected margin (mm), distal resected margin (mm), radicality(R0/R1/R2)

(12) Intraoperative complications (occurring from skin incision to skin closure) including:

surgery-related complications: intraoperative hemorrhage and injury: A. Vascular injury: A vascular injury is defined as a blood vessel with either a blood vessel clamp or a titanium clamp closure and an intra-cavity suture or any other method to control the bleeding. B. Organ damage: maybe including diaphragmatic injury, esophageal injury, duodenal injury, colon injury, small intestine injury, spleen injury (excluding <1/3 spleen ischemia), liver injury, pancreatic injury, gallbladder injury, kidney damage etc.

C. Tumor rupture: tumor envelope Integrity damage

air abdominal-related complications: high-blood carbonate, mediastinal emphysema, subcutaneous emphysema, air embolism, respiratory circulation instability caused by abdominal pressure.

Anesthesia-related complications: Allergic reactions.

(13) Intraoperative death (occurring during the time period from skin cutting to skin stitching completion) regardless of reason.

9.3.4 Postoperative LN harvest

LN–bearing soft tissue was separated from the resected specimens in vitro in accordance with the definitions of the Japanese classification guidelines.[17] Fluorescent LNs were retrieved from each station directly through NIR imaging. LNs emitted fluorescence were classified as fluorescent LNs. Stations containing fluorescent LNs were classified as fluorescent stations. Surgeons examined all specimens, which were immediately sent to the pathology department. All pathological examinations were performed in a standard manner.[17]

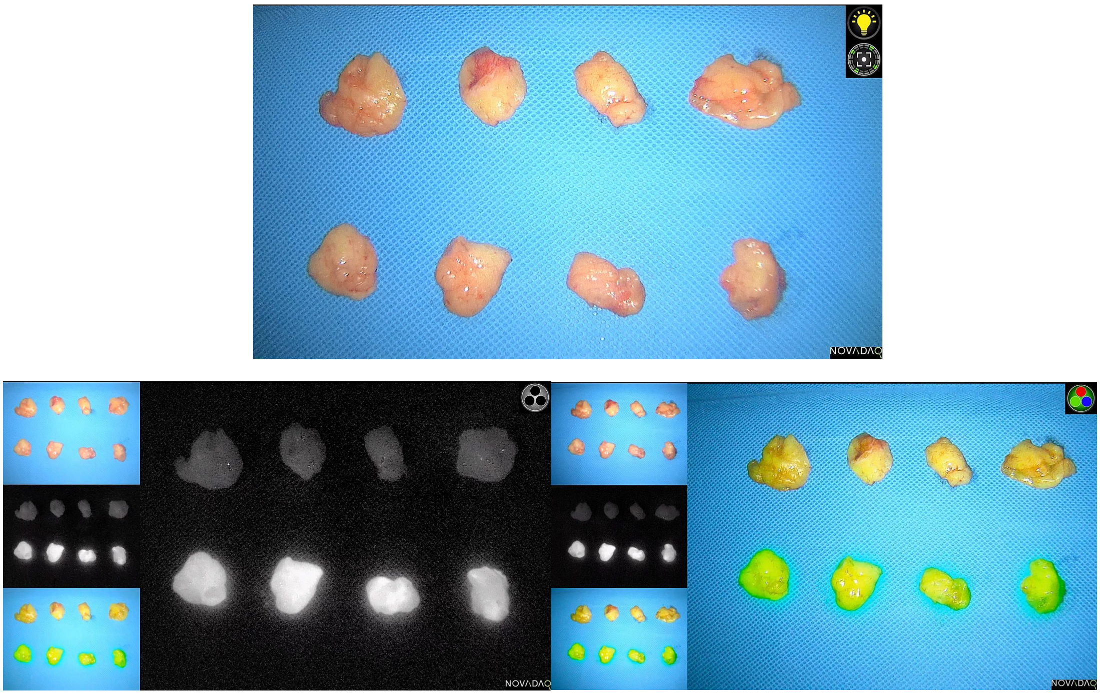

Figure 4. Postoperative LN sorting (fluorescent LNs and non-fluorescent LNs)

9.4 Postoperative management (same for both groups)

9.4.1 The use of prophylactic analgesics

Continuous postoperative prophylactic intravenous analgesia is allowable but not mandatory within postoperative 48 hours; its dose, type, and rate of infusion should be determined by the anesthesiologist according to clinical practices and specific patient conditions. The repeated use of prophylactic analgesics is not allowed beyond 48 hours after the end of the surgery unless it is judged necessary

9.4.2 Fluid replacement and nutritional support

Postoperative fluid infusion (including glucose, insulin, electrolytes, vitamins, etc.) or nutritional support (enteral/parenteral) will be performed based on the doctor's

experience and routine clinical practices and is not specified in this study. After oral feeding, it is allowable to stop or gradually reduce fluid infusion/nutritional support.

9.4.3 Post-operative rehabilitation management

Management methods of incision, stomach, and abdominal drainage tube: Follow regular diagnosis and treatment approaches. Eating recovery time, diet transition strategies: Follow regular diagnosis and treatment approaches.

9.4.4 Discharge standard

Patients needed to meet the following criteria for discharge: 1) satisfactory intake of a soft diet. 2) move around their bed. and 3) absence of complications by routine clinical examinations. This information will be recorded in the CRF.

9.4.5 Postoperative observation items

Definition of “postoperative day n”: One day from 0:00 to up to 24:00. Up to 24:00 on the day of surgery is “postoperative day 0;” the next day from 0:00 to up to 24:00 is “postoperative day 1;” and so on. From the first postoperative day until hospital discharge, the research assistant should timely fill in the following items and specific observation items including:

(1) Pathologic results:

Original lesion tissue typing, Distant metastasis, and parts, NIH Hazard grading, Radical surgery degree (R0/R1/R2)

(2) Postoperative complications:

Postoperative complications are divided into and short-term complications after surgery and long-term complications after surgery. Short-term is defined as within 30 days of surgery or the first discharge if the hospital days > 30 days. Long-term is defined as the period from 30 days or more after the operation, or the first discharge (the hospital days after surgery >30 days) to 3 years after the operation.

Classification and name of complication	Diagnostic criteria
Abdominal bleeding	Intra-abdominal hemorrhage requires a blood transfusion, emergency endoscopy, or surgical intervention to eliminate anastomotic bleeding.
Anastomotic bleeding	The postoperative gastrointestinal decompression tube continued to have fresh red blood outflow; the hemoglobin drops more than 1g/dl.
Gastrointestinal anastomotic stoma Fistula	Using gastrointestinal angiography to see contrast agent leak out from the anastomosis, or the blue drainage outflow through tube after oral Methylene blue to eliminate the possibility duodenal stump fistula and intestinal fistula.
Duodenal Stump Fistula	Using gastrointestinal angiography to see contrast agent leak out from the duodenal stump to eliminate the anastomotic fistula or intestinal

	fistula.
Intestinal fistula	Using gastrointestinal angiography to see the blue drainage outflow through tube after oral Methylene blue to eliminate anastomotic fistula and duodenal stump fistula.
Stenosis of Anastomosis	Endoscopic examination with a 9.2-mm endoscopy not passing through the anastomosis to eliminate recurrence of tumors.
Input jejunal loop obstruction	Abdominal pain, abdominal distension, vomiting, and other symptoms. Abdominal flat to see the right upper abdomen expansion of the intestinal loop, and there is a liquid plane or a visible input loop jejunum giant expansion by barium meal examination.
Intestinal obstruction after the operation	Abdominal X-ray shows a plurality of liquid planes and the phenomenon of intestinal effusion with visible isolated, fixed, swelling of the intestinal loop. Total Abdominal CT showed edema, thickening, adhesion of intestinal wall, accumulation of gas in the intestinal cavity, uniform expansion of bowel, and intra-abdominal exudation.
Early dumping syndrome	Combined the symptoms of sweating, heat, weakness, dizziness, palpitations, heart-swelling feeling, vomiting, abdominal colic or diarrhea with the signs of tachycardia, blood pressure micro-rise, breathing a little faster sign after meal 15-30 minutes, and solid-phase radionuclide gastric emptying scanning tips stomach quickly emptying.
Late dumping syndrome	Feeling hungry, flustered, out of sweating 2-3 hours after the meal. Blood sugar is less than 2.9mmol/L, excluding other diseases that cause hypoglycemia.
Intestinal ischemia and necrosis	Under the digestive endoscopy, the intestinal mucosa congestion, edema, bruising, mucosal hemorrhage, the mucous membrane being dark red, the vascular network disappearing, can have part mucosal necrosis, following with mucosal shedding, ulcer formation with annular, longitudinal, snake and scattered in the ulcer erosion.
Internal hernia	Postoperative CT findings of cystic or cystic and solid mass, and intestinal aggregation, stretching, translocation, abnormal mesenteric movement, and thickening of the blood vessel.
Alkaline reflux esophagitis	1. Endoscopic examination and biopsy of the upper gastrointestinal tract showed evidence of inflammation of the mucous membranes and gastrointestinal metaplasia; 2. CT scan and gastrointestinal barium meal examination showed no expansion or obstruction of the input loop.
Incision splitting	Including partial dehiscence of the incision and full-layer dehiscence.
Incisional hernia of the abdominal wall	The swelling tumor showing in the surgical scar area or abdominal wall swelling when standing or force. CT shows ventral wall continuity interruption and hernia content extravasation.
Incision infection	Thickening of the soft tissue at the incision, in or below the incision of gas, exudation, swelling of the incision or pus from the incision

	extrusion, or secretion culture of pathogenic bacteria.
Lymphatic leakage	A chyle test when abdominal drainage fluid exceeded 300 ml/day for 5 consecutive days after postoperative day 3.
Pneumonia	Complies with one of the following two diagnostic Criteria: 1. Auscultation/percussion voiced + one of the following: fresh sputum or sputum character changes; blood culture (+); bronchoalveolar lavage fluid, anti-pollution sample brush, biopsy specimens cultured pathogenic bacteria. 2. Chest film hints of new or progressive infiltration + one of the following: fresh sputum or sputum character changes, blood culture (+), bronchoalveolar lavage fluid, anti-pollution sample brush, biopsy specimens cultured pathogenic bacteria; isolate the virus or detect IgM, IgG (+) of respiratory viral.
Acute pancreatitis	Irritability, abdominal pain, anti-jumping pain, fever, leukocyte increase, and blood amylase increased occurring and diagnosed by ultrasound or CT within 3 days after surgery.
Acute cholecystitis	Serum bilirubin exceeding 85μmol/l and ultrasound examination shows gallbladder enlargement, wall thickness, signal and sound shadow of gallbladder stone, bile internal sediment, gallbladder contraction bad, etc.
Pleural effusion/infection	CT scan showed the localized fluid low density area of the thoracic cavity, which could accompany with gas, and culture pathogenic bacteria in thoracic endocrine.
Abdominal infection	There is at least one of the following evidence in the abdominal cavity within 30 days after operation: 1. discharge of pus, with/without microbiological examination; 2. bacterial culture positive; 3. diagnosed by detection, pathology, imaging findings.
Pelvic infection	Symptoms of systemic infection or rectal irritation, combined with a rectal finger examination and touching tenderness, or a married woman with a posterior vault to extract pus-based fluid.
Sepsis	The following two conditions are available: 1. There is evidence of active bacterial infection, but the blood culture does not necessarily appear pathogenic bacteria; 2. meeting two of the following four items at the same time: (1). body temperature >39. 0°C or ＜35.5 °C for 3 consecutive days, (2). heart rate > 120 times/min; (3). total white blood cells >12. 0*10^9/L or <4.0*10^9/l, wherein neutrophils >0. 80, or naïve granular cells >0. 10; (4). Respiratory frequency > 28 times/min.
Urinary system infection	Symptoms of urine frequency, urgency and urine pain, etc. and urine bacteria culture colony count 1000~10 million/ml in the absence of antibiotics; No symptoms of urine frequency, urgency and urine pain, etc, urine bacterial culture colony count ≥ 100,000/ml.
Pancreatic fistula	The level of amylase in the drainage fluid is more than three times the

	normal level.
Bile fistula	Symptoms of abdominal distension, Abdominal pain, tenderness, anti-jumping pain, muscle tension, abdominal puncture, or drainage fluid for bile.
Celiac fistula	The drainage fluid is milky white, and more than 200ml/d and does not decrease for 48 hours, the celiac qualitative test is positive, and the level of triglyceride >110 mg/dL at the same time.
Nutritional disorder after gastrectomy	In the presence of weight loss, anemia, malnutrition bone disease, vitamin A deficiency, and other symptoms, laboratory tests suggest that the intestinal absorption function test is abnormal, excluding other causes of nutritional disorders.
Bone disease after gastrectomy	Lumbar back pain, length shortening, kyphosis, bone fractures, and other symptoms. Bone density decreased combining with elevated alkaline phosphatase and serum calcium reduction, the concentration of serum 25-(O1) D3 and 1,25-(O1) 2D3 increasing and the serum parathyroid hormone increasing. Exclusion of bone disease caused by other causes.
Subcutaneous emphysema	visible the irregular speckle shadow under the skin in the horizontal flat sheet.
Mediastinal emphysema	In the posterior and anterior flat fame, a long narrow gas shadow rises to the neck soft tissue along the mediastinal side, forming a thin-line dense shadow. In the lateral flat, there was a visible and clear band between the heart and the sternum. The CT examination, if necessary, shows a gas density line-like shadow around the mediastinal and mediastinal pleura closing to the direction of the lung field.
Postoperative hemorrhage	An amount of hemorrhage exceeding 300 ml.
Postoperative cardiac dysfunction	The symptom of sinus tachycardia, sinus bradycardia, supraventricular tachycardia, ventricular tachycardia, and other arrhythmias, or heart failure preoperatively non-existing and postoperatively appearing, and other causes of the above-mentioned manifestations are excluded.
Hepatic dysfunction	Bilirubin increasing and the levels of AST and ALT >5 times after operation and these symptoms no existing before surgery.
Kidney function failure	Postoperative continuing renal function insufficiency, blood creatinine rising 2mg/dl, or acute renal failure needing dialysis treatment.
Cerebral embolism	Acute onset, hemiplegia, aphasia, and other focal neurological function deficits. Embolism site has low-density infarction, of which border is not clear and no obstructions performance within 24-48 hours after the onset.
Pulmonary embolism	Characteristics of dyspnea, chest pain, syncope, shortness of breath, right ventricular insufficiency and hypotension, pulmonary angiography revealed a filling defect.

Venous thrombosis of lower extremities	Local tenderness, swelling, purple skin color, combined with intravenous angiography to show the filling defect.
Mesenteric arterial embolization	For patients with acute abdominal pain, vomiting, diarrhea, and abdominal x-ray of intestinal tract filling with gas or existing liquid level, abdominal angiography revealed a filling defect.
DIC	1.There are basic diseases easily leading to DIC, 2. There are more than two clinical performances: (1) severe or multiple bleeding tendencies; (2) Microcirculation disorder or shock cannot be explained by the original disease. (3) Extensive skin mucosal embolism, focal ischemic necrosis, shedding and ulcer formation, or unexplained lung, kidney, brain, and other organ failure. (4) anticoagulant treatment.is effective. 3. The laboratory meets the following conditions: (1) there are 3 or more experimental abnormalities: platelet count, prothrombin time, activated partial coagulation enzyme time, thrombin time, fibrinogen level, D-two poly, and (2) difficult or special cases for a special examination.
Other	Complications are other than the above complications, which do not exist before surgery but appear after surgery.

The severity of complication is graded according to Clavien–Dindo complication scoring system,[26]

IIIa level and above are defined as a serious complication

I: Any deviation from the normal postoperative course without the need for pharmacologic treatment or surgical, endoscopic, and radiologic interventions. Allowed therapeutic regimens are drugs as antiemetics, antipyretics, analgesics, and diuretics, and electrolytes and physiotherapy. This grade also includes wound infections opened at the bedside.

II: Requiring pharmacologic treatment with drugs other than such allowed for grade I complications. Blood transfusions and total parenteral nutrition are also included.

III: Requiring surgical, endoscopic, or radiologic intervention.

IIIa: Intervention not under general anesthesia.

IIIb: Intervention under general anesthesia.

IV: Life-threatening complication (including CNS complications) requiring IC (intermediate care) / ICU (intensive care unit) management.

IVa: Single organ dysfunction (including dialysis).

IVb: Multiple organ dysfunction.

V: Death as a result of complications.

(3) Blood test items (At postoperative day 1, 3, 5)

Peripheral blood routine assessment: Hb, RBC, WBC, LYM, NEU, NEU%, and PLT、MONO. Blood biochemistry: albumin, total bilirubin, AST, ALT, creatinine, urea nitrogen, fasting blood glucose, potassium, sodium, chlorine, calcium, and CRP.

(4) Postoperative rehabilitation evaluation:

Time to first ambulation (hours), time to first flatus (hour), time to a liquid diet,

time to semi-liquid diet (hour), daily body temperature maximum from surgery to out-patient (℃), time to removal of a gastric tube (d), the daily volume of gastric drainage (ml), time to removal of the abdominal drainage tube (d), the daily volume of drainage (ml). Blood transfusion volume (ml) from the end of surgery to postoperative discharge: a transfusion event is defined as an infusion of the red blood cell suspension (ml) or whole blood (ml). Postoperative hospital stays (days): periods form surgery day to first discharge day

9.5 Follow-Up

9.5.1 Follow-up Period and strategy

Follow-up visits will be completed by special persons for all cases selected in this study. All patients are followed up with every 3 months during the first 2 years and then every 6 months beyond the third year (1、 3、 6、 9、 12、 15、18、21、24、30 and 36 months after the operation). This study suggests that the above examinations should be conducted in the patient's primary surgical research center, but does not exclude outer court review. For Outer Court review, it recommended that visiting the hospital as a three-level hospital, and this information will be recorded by the follow-up specialist. The occurrence of tumor recurrence or metastasis and the survival status of all patients are evaluated and recorded according to the results of the various examinations. Patients who refuse to follow the protocol should be recorded as lost to follow-up, and at the end of the study, these cases should be analyzed together with cases lost to follow-up in line with the criteria of this study.

9.5.2 Assessment items during the follow-up

(1) Systematic physical examination:

The doctor in charge will regularly conduct a systematic physical examination at the time of each follow-up, giving particular attention to superficial LNs, abdomen, and signs of metastases, among others.

(2) Blood test items:

Peripheral blood routine assessment: Hb、RBC、WBC、LYM、NEU、NEU%、PLT、MONO

Biochemistry: Albumin, total bilirubin, indirect bilirubin, direct bilirubin, AST, ALT, creatinine, urea nitrogen, total cholesterol, triglycerides, fasting blood glucose, potassium, sodium, chlorine, calcium, serum tumor markers: CEA、CA19-9、CA72-4、CA12-5、AFP

(3) Imaging items:

Whole abdomen (including cavity) CT (thickness of 10 mm or less, in case of contrast agent allergy, CT horizontal scanning is only allowable or conversion to MRI). Upper gastrointestinal endoscopy (histopathological biopsy, endoscopic ultrasonography when necessary). Chest X-ray (AP and lateral views): lung field condition. Other means of evaluation: gastrointestinal radiography, ultrasonography of other organs, whole-body bone scanning, and PET-CT, among others used at physician’s discretion.

9.5.3 Follow-up process

Postoperative	3 months	6 months	9 months	12 months	15 months	18 months	21 months	2 years	2 years and a half	3 years
Date of the actual visit										
Physical examination										
Blood Routine										
Blood biochemistry										
Tumor Markers										
Chest slices										
Upper digestive tract endoscopy										
Abdominal CT										
Full abdominal ultrasound										
Other (if necessary)										

9.6 Post-operative adjuvant therapy

9.6.1 Indications for postoperative adjuvant chemotherapy

After completion of the surgical treatment, according to the postoperative pathological results, subjects among the R0 resection cases that are stage II and above are administered postoperative adjuvant chemotherapy according to the provisions of this program.

For cases of non-R0 resection or recurrence after R0 resection, this study does not stipulate the follow-up treatment plan; the research center decides on the action to be taken according to the clinical treatment routine.

9.6.2 Postoperative adjuvant chemotherapy

This study uses a combination of chemotherapy based on 5-FU (5-fluorouracil) and recommends the SOX regimen.

The adjuvant chemotherapy cycle is half a year (6 months postoperatively).

In cases of good physical and tolerable conditions, chemotherapy is first started within 8 weeks after surgery and then according to the regularity of the chemotherapy cycle.

During the chemotherapy period, tumor recurrence should be assessed according

to the follow-up plan.

When tumor recurrence occurs during chemotherapy, the adjuvant chemotherapy regimen of this study is discontinued. The follow-up treatment is decided by the research center according to the clinical treatment routine. This study does not make regulations, but the cause and follow-up treatment plan should be recorded in the CRF.

If there is no recurrence during chemotherapy, adjuvant chemotherapy is terminated after 6 months, and the follow-up plan continues.

Adjuvant chemotherapy requires written approval from the patient.

Subjects that refuse postoperative adjuvant chemotherapy or do not complete the adjuvant chemotherapy are not excluded from this study, but the cause is marked and recorded in the CRF.

For elderly patients (70 years and older), considering differences in the physical fitness of the elderly and ensuring the safety of patients, the research center decides according to the clinical treatment routine. This study does not recommend or stipulate any chemotherapy regimen for patients of this age.

Patients who choose adjuvant chemotherapy, irregular chemotherapy, or a non-first-line regimen are not excluded from the study, but the FUGES-019 Efficacy and Safety Evaluation Committee is obliged to monitor patient safety during follow-up. The patient's chemotherapy medication must be recorded in the CRF.

The principles of processing in terms of the method of administration of adjuvant chemotherapy, toxic reactions, and dose adjustment with intolerance are implemented according to the original literature on drug toxicity and dose adjustment for each chemotherapy regimen. This study does not regulate these principles.

9.6.3 Safety Evaluation Indicators of Postoperative Adjuvant Chemotherapy

The safety evaluation indicators for patients enrolled in the study should be immediately filled out by the investigators before and after each postoperative adjuvant chemotherapy cycle, with specific items including the following:

(1) Performance Status (ECOG)

(2) Subjective and objective status (according to the records of CTCAE v3.0 Short Name)

(3) Blood tests:

Peripheral venous blood assessment: Hb, RBC, WBC, LYM, NEU, NEU%, PLT, MONO.

Blood biochemistry: albumin, total bilirubin, AST, ALT, creatinine, urea nitrogen,

fasting blood glucose, serum tumor markers (CEA, CA19-9, CA72-4, CA12-5, AFP)

(4) Safety evaluation items to be implemented during chemotherapy when necessary (refer to CTCAE v3.0):

Neurotoxicity

Cardiovascular system (cardiac toxicity, ischemic heart disease, etc.)

Bone marrow suppression and infections due to immune dysfunction

Others

9.7 Study calendar

Observation Stage	Performance Status	Blood biochemistry	Tumor markers	Electrocardiogram, respiratory function	Upper gastrointestinal endoscopy	Chest X-ray, full abdominal CT Or ultrasound	Eligibility confirmation notice	Preoperative, postoperative complications	Adverse chemotherapy events	CRF- Preoperative	CRF-Intraoperative	CRF- Postoperative	CRF- treatment end	CRF- follow-up observation surgery
Selection Application	○	○	○	○	○	○								
After selection and prior to surgery							○			○				

Intraoperative period									○			○			
Early postoperative period									○				○	○	
Before postoperative first chemotherapy		○	○	○			○								
Regular chemotherapy		○	○	○						○					
Follow-up period Postoperative advanced stage	At postoperative 1 month (±7 days)	○	○	○			○		○						○
	At postoperative 3 months (±15 days)	○	○	○					○						○
	At postoperative 6 months (±15 days)	○	○	○			○		○						○
	At postoperative 9 months (±15 days)	○	○	○					○						○
	At postoperative 1 year (±15 days)	○	○	○			○		○						○
	At postoperative 15 months (±15 days)	○	○	○					○						○
	At postoperative 18 months (±15 days)	○	○	○			○		○						○

	At postoperative 21 months (±15 days)	○	○	○					○						○
	At postoperative 2 years (±15 days)	○	○	○				○	○						○
	At postoperative 2.5 years (±15 days)	○	○	○				○	○						○
	At postoperative 3 years (±15 days)	○	○	○				○	○						○

○: must do

9.8 Definitions involved in SOP

9.8.1 ECOG performance status score

According to the simplified performance status score scale developed by the ECOG, the patients' performance status can be classified into 6 levels, namely 0-5, as follows:

0: Fully active, able to carry on all pre-disease performance without restriction

1: Restricted in physically strenuous activity but ambulatory and able to carry out work of a light or sedentary nature, e.g., light housework, office work

2: Ambulatory and capable of all self-care but unable to carry out any work activities. Up and about more than 50% of waking hours

3: Capable of only limited self-care, confined to bed or chair more than 50% of waking hours

4: Completely disabled. Cannot carry on any self-care. In total, confined to bed or chair

5: Dead

Patients at levels 3, 4, and 5 are generally considered to be unsuitable for surgical treatment or chemotherapy.

9.8.2 ASA classification

According to the patients' physical status and surgical risk before anesthesia, the American Society of Anesthesiologists (ASA) has categorized patients into 5 levels (I-V levels):

Class I: Well-developed patients with physical health and normal function of various organs, with a perioperative mortality rate of 0.06% -0.08%.

Class II: Patients with mild complications and good functional compensation in addition to surgical diseases, with a perioperative mortality rate of 0.27% -0.40%.

Class III: Patients with severe complications and restricted physical activity but still capable of coping with day-to-day activities, with a perioperative mortality rate of 1.82% -4.30%.

Class IV: Patients with serious complications who have lost the ability to perform day-to-day activities, often have life-threatening conditions, and a perioperative mortality rate of 7.80% -23.0%.

Class V: Moribund patients either receiving surgery or not, have little chance for survival, and a perioperative mortality rate of 9.40% -50.70%.

Generally, Class I/II patients are considered good for anesthesia and surgical tolerance, with a smooth anesthesia process. Class III patients are exposed to some anesthesia risks; therefore, good preparations should be fully made before anesthesia, and effective measures should be taken to prevent potential complications during anesthesia. Class IV patients are exposed to the most risks, even if good preoperative preparations are made, and have a very high perioperative mortality rate. Class V patients are moribund patients and should not undergo elective surgery.

9.8.3 Oncology-related definitions

In this study, tumor staging is based on AJCC-8th; surgical treatment follows the Japanese gastric cancer treatment guidelines 2018 (5th edition), and other writing and recording principles follow the Japanese classification of gastric carcinoma: 3rd English edition.

9.8.3.1 Primary focus location

The greater and lesser curvature of the stomach is divided into three equal parts, the U (upper), M (middle), and L (lower) areas, connected to the corresponding points. Esophagus and duodenum infiltration are recorded as E (esophagus), and D (duodenum), respectively. If the lesions are located in two or more adjacent areas, they should be recorded in the order of the main portions of the lesions.

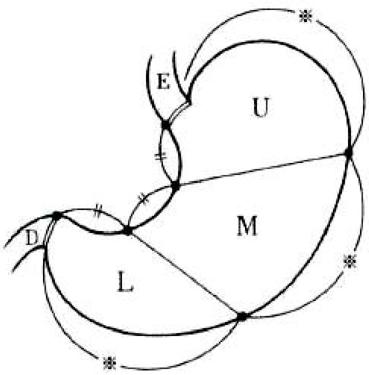

図1. 胃の3領域区分

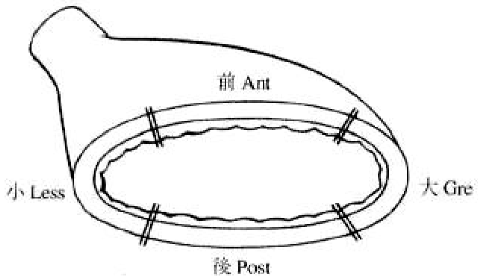

図2. 胃壁の断面区分

Figure 5. Division of the Three Areas of the Stomach

9.8.3.2 Tumor staging record

9.8.3.2.1 Recording principle

The two staging records for clinical classification and pathological classification involve T (invasion depth), N (regional LN), and M (distant metastasis), which are expressed in Arabic numerals and denoted as x if indefinite.

Clinical classification	Pathological classification
Physical examination X-ray, endoscopy, diagnostic imaging<br>laparoscopy, intraoperative observations (laparotomy/laparoscopy), biopsy, cytology, biochemistry, biology examination	Pathological diagnosis of the endoscopic/surgical specimens<br>Intraperitoneal exfoliative cytology

9.8.3.2.2 Records of tumor invasion depth

Tumor invasion depth is defined as follows:

TX: Unknown cancer invasion depth

T0: No cancer found

T1: Cancer invasion is only confined to the mucosa (M) or the submucosal tissue (SM)

- T1a: Cancer invasion is only confined to the mucosa (M)
- T1b: Cancer invasion is confined to the submucosal tissue (SM)

T2: Cancer invasion exceeds the submucosal tissue but is only confined to the inherent muscular layer (MP)

T3: Cancer invasion exceeds the inherent muscular layer (MP) but is only confined to the subserosal tissue (SS)

T4: Cancer invasion involves the serosa (SE) or direct invasion of adjacent structures (SI)

- T4a: Cancer invasion involves only the serosa (SE)

◆ T4b: Cancer directly invades the adjacent structures (SI)

9.8.3.2.3 Records of tumor metastasis

(1) LN metastasis:

NX: Number of LN metastases is unknown

N0: No LN metastasis

N1: LN metastasis of 1-2 areas

N2: LN metastasis of 3-6 areas

N3: LN metastasis of 7 and more areas

◆ N3a: LN metastasis of 7-15 areas

◆ N3b: LN metastasis of 16 and more areas

Anatomical definitions of LN stations

No.	Name	Definition
1	Right paracardial LNs	LNs including those along the first branch of the ascending limb of the left gastric artery
2	Left paracardial LNs (along the left gastric artery)	LNs including those along the esophagocardiac branch of the left subphrenic artery
3a	Lesser curvature LNs	LNs along the branches of the left gastric artery
3b	Lesser curvature LNs (along the right gastric artery)	LNs along the 2nd branch and distal part of the right gastric artery
4sa	Left greater curvature LNs (short gastric artery)	LNs along the short gastric arteries (perigastric area)
4sb	Left greater curvature LNs (along the left gastroepiploic artery)	LNs along the left gastroepiploic artery (perigastric area)
4d	Right greater curvature LNs (along the right gastroepiploic artery)	LNs along the 2nd branch and distal part of the right gastroepiploic artery
5	Suprapyloric LNs	LNs along the 1st branch and proximal part of the right gastric artery
6	Infrapyloric LNs	LNs along the first branch and proximal part of the right gastroepiploic artery down to the confluence of the right gastroepiploic vein and the anterior superior pancreatoduodenal vein
7	Left gastric artery trunk LNs	LNs along the trunk of left gastric artery between its root and

		the origin of its ascending branch
8a	Anterosuperior part of the common hepatic artery LNs	Anterosuperior LNs along the common hepatic artery
8p	Posterior part of the common hepatic artery LNs	Posterior LNs along the common hepatic artery
9	Celiac artery LNs	Celiac artery LNs
10	Splenic hilar LNs	Splenic hilar LNs including those adjacent to the splenic artery distal to the pancreatic tail, and those on the roots of the short gastric arteries and those along the left gastroepiploic artery proximal to its 1st gastric branch
11p	Proximal splenic artery LNs	LNs from its origin to halfway between its origin and the pancreatic tail end
11d	Distal splenic artery LNs	LNs from halfway between its origin and the pancreatic tail end to the end of the pancreatic tail
12a	Hepatoduodenal ligament LNs (along the proper hepatic artery)	LNs along the proper hepatic artery, in the caudal half between the confluence of the right and left hepatic ducts and the upper border of the pancreas
12b	Hepatoduodenal ligament LNs (along the bile duct)	LNs along the bile duct, in the caudal half between the confluence of the right and left hepatic ducts and the upper border of the pancreas
12p	Hepatoduodenal ligament LNs (along the portal vein)	LNs along the portal vein in the caudal half between the confluence of the right and left hepatic ducts and the upper border of the pancreas
13	LNs on the posterior surface of the pancreatic head cranial	LNs on the posterior surface of the pancreatic head cranial to the duodenal papilla
14v	LNs along the superior mesenteric vein	LNs along the superior mesenteric vein
15	LNs along the middle colic vessels	LNs along the middle colic vessels
16a1	Paraaortic LNs a1	Paraaortic LNs in the diaphragmatic aortic hiatus
16a2	Paraaortic LNs a2	Paraaortic LNs between the upper margin of the origin of the celiac artery and the lower border of the left renal vein
16b1	Paraaortic LNs b1	Paraaortic LNs between the lower border of the left renal

		vein and the upper border of the origin of the inferior mesenteric artery
16b2	Paraaortic LNs b2	Paraaortic LNs between the upper border of the origin of the inferior mesenteric artery and the aortic bifurcation
17	LNs on the anterior surface of the pancreatic head	LNs on the anterior surface of the pancreatic head beneath the pancreatic sheath
18	Below the pancreas	LNs along the inferior border of the pancreatic body
19	Infradiaphragmatic LNs	Infradiaphragmatic LNs predominantly along the subphrenic artery
20	Paraesophageal LNs	Paraesophageal LNs in the diaphragmatic esophageal hiatus
110	Paraesophageal LNs in the lower thorax	Paraesophageal LNs in the lower thorax
111	Supradiaphragmatic LNs	Supradiaphragmatic LNs separate from the esophagus
112	Posterior mediastinal LNs	Posterior mediastinal LNs separate from the esophagus and the esophageal hiatus

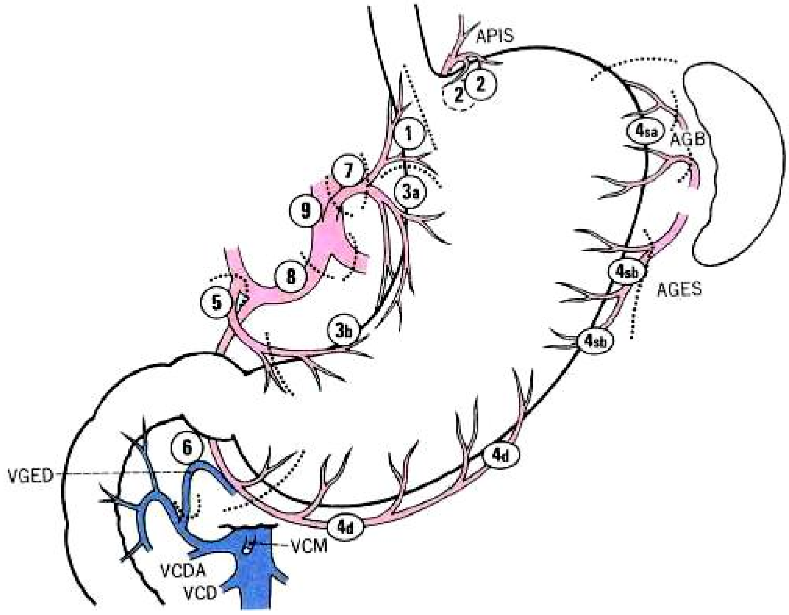

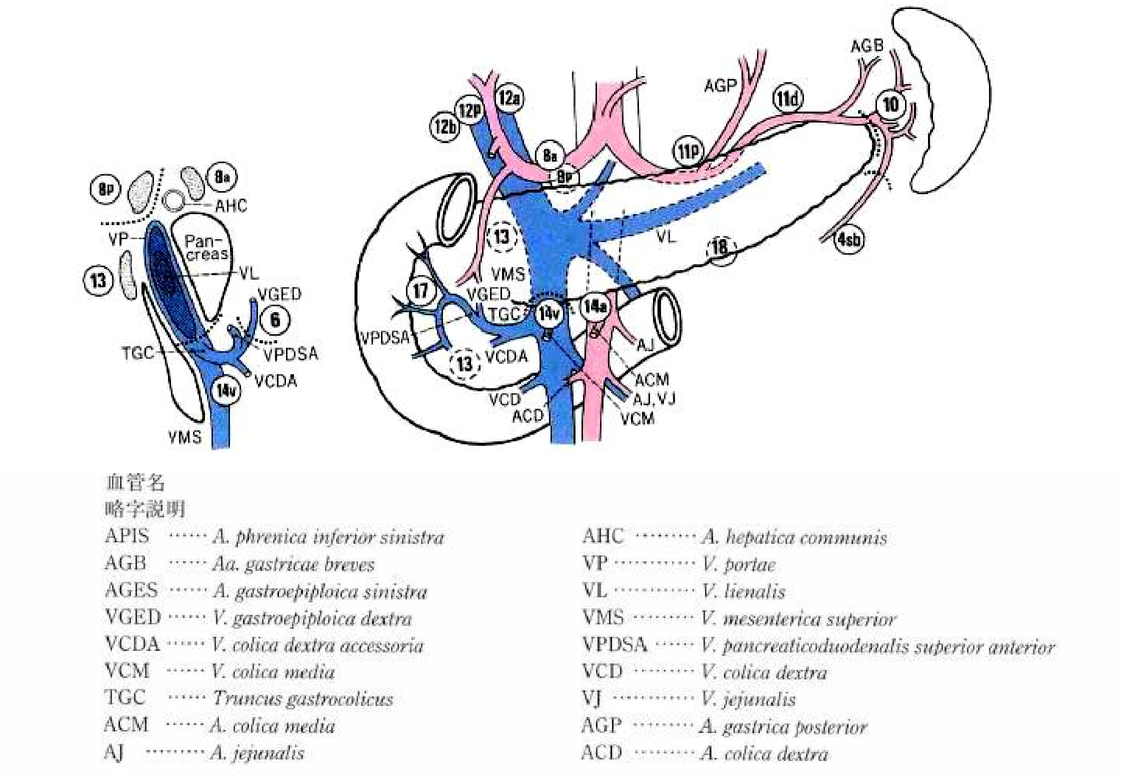

Figure 6. Lymph node station

(2) Distant metastasis

M0: No distant metastasis outside of the regional LNs

M1: Distant metastasis outside of the regional LNs

MX: The Presence of distant metastasis is unclear

Record the specific sites under the M1 condition: peritoneum (PER), liver (HEP), distant LN (LYM), skin (SKI), lung (PUL), bone marrow (MAR), bone (OSS), pleura (PLE), brain (BRA) and meninges (MEN), intraperitoneal exfoliated cells (CY), and others (OTH). Note: A positive examination result for intraperitoneal exfoliated cells is recorded as M1.

9.8.3.2.4 Tumor Staging

Pathological (pTNM)					
T/M	N0	N1	N2	N3a	N3b
T1	IA	IB	IIA	IIB	IIIB
T2	IB	IIA	IIB	IIIA	IIIB
T3	IIA	IIB	IIIA	IIIB	IIIC
T4a	IIB	IIIA	IIIA	IIIB	IIIC
T4b	IIIA	IIIB	IIIB	IIIC	IIIC
M1	IV	IV	IV	IV	IV

9.8.3.3 Pathologic types and classifications

9.8.3.3.1 Type

Papillary adenocarcinoma

Tubular adenocarcinoma

Mucinous adenocarcinoma

Signet ring cell carcinoma

Poorly differentiated carcinoma

9.8.3.3.2 Grading

GX classification is not possible to assess

G1 well-differentiated

G2 moderately differentiated

G3 poorly differentiated

G4 undifferentiated

9.8.3.4 Evaluation of Radical Level (Degree)

9.8.3.4.1 Recording the Presence or Absence of Cancer Invasion on the Resection Stump

(1) Proximal incisional margin (PM: proximal margin)

PM (-): No cancer invasion found on the proximal incisional margin

PM (+): Cancer invasion found on the proximal incisional margin

PM X: Unknown cancer invasion on the proximal incisional margin

(2) Distal incisional margin (DM: distal margin)

DM (-): No cancer invasion found on the distal incisional margin

DM (+): Cancer invasion found on the distal incisional margin

DM X: Unknown cancer invasion on the distal incisional margin

9.8.3.4.2 Radical Records

Postoperative residual tumor, denoted with R (residual tumor): R0: curative resection; R1, R2: non-curative resection.

RX: cannot be evaluated

R0: no residual cancer

R1: microscopic residual cancer (positive margins, peritoneal lavage cytology positive)

R2: macroscopic residual cancer

10 Statistical analysis

10.1 Definition of the population

(1) ITTP, intent-to-treat population

(2) MITTP, modified intent-to-treat population

(3) PPP, per-protocol population

(4) SAP, safety analysis population

10.2 Statistical analysis plan

- Statistical software: We will use Epidata3.0 to establish a database and to input data, and we will use the SPSS statistical software, version 22.0 (SPSS Inc) and the R software environment, (R Foundation for Statistical Computing) to perform statistical analyses.
- Basic principle: The method of differential testing was adopted. The safety population of the study consists of the patients who receive safety evaluation data after the intervention. Descriptive statistics and two-sided tests were conducted for the safety indicators and the incidence of adverse reactions. A p-value <0.05 is considered statistically significant. The confidence interval of the parameters is estimated with a 95% confidence interval.
- Shedding analysis: Total shedding rate of two groups and loss rate due to adverse events will be compared using pearsonχ^2 test
- Statistical analysis of population division: baseline data and effective analysis using MITT analysis. The main therapeutic indicators are analyzed using both MITT and PP analysis. But based on the conclusion of MITT analysis. If MITT analysis and PP analysis of the conclusions are consistent, it can increase the credibility of the conclusion. The data of laboratory examination, adverse events, and adverse reactions were analyzed by SAP. The incidence rate of adverse reactions uses SAP as the denominator.
- Method of outlier determination: the observation value is greater than P75 or less than P25, and the exceed value more than 3 times of the quartile spacing (=p75-p25), which will be sentenced to outlier data. During the analysis, the sensitivity analysis is used for outlier data, namely analyzing outcomes including or excluding, outlier data. and if the results are not contradictory, the data is retained; if the contradiction, it depends on the specific circumstances.
- Descriptive statistics: The measurement data gives the mean, the standard deviation, and the confidence interval, and the minimum value, the maximum value, the P25, the median, and the P75 are given when necessary; matched data also gives the mean and standard deviation of the gap-value, and the median and average rank of the Non-parametric method. The nominal-scale data gives the frequency distribution and the corresponding percentages. The level data gives the frequency distribution and the corresponding percentages, as well as the median and the average rank. Qualitative data give a positive rate, positive number, and denominator numbers. The survival data gives the number of events, the number of deletions, the median survival time, and the survival rate.
- Subgroup analysis: Sub-group analysis is to find the factors that may affect prognostic according to the specific circumstances of the data。

- Missing values handling: This study does not fill in missing values
- Effective analysis: Using Log-rank test for single-factor analysis of Survival Time Data, using Cox regression model Analysis for multi-factor analysis. Quantitative data using t-test or t' Test (variance is not homogeneous), qualitative data using Pearson c2 test, grade data using Wilcoxon rank test.
- Safety analysis: counting adverse response incidence and incidence of adverse events and make a list to describe the adverse events occurring in the study. describe the results of the laboratory tests before and after the normal/abnormal changes and the relationship between the abnormal changes and drugs in the research, and make a list of the "normal/abnormal" changes that occurred in the study. More detailed statistical analysis is shown in the statistical analysis plan.

11 Data management

11.1 Case Report Form (CRF)

11.1.1 CRF Types and Submission Deadline

CRFs used in this study and their submission deadlines are as follows:

(1) Case Screening: 7 days before surgery (time frame of three days)

(2) Enrolling: submitted to the data center two days before surgery

(3) Surgery: within 1 day after surgery

(4) Postoperative discharge: within three days after the first discharge

(5) Follow-up records: 7 days after each specified follow-up time point

11.1.2 Method of transmission of CRF

In this study, the paper CRF form is used for information and data transmittal.

11.1.3 Revision of CRF

After the start of the study, if the CRF is found to lack items that are then deemed pertinent, under the premises of ensuring the amendment of the CRF does not cause medical and economic burden and increased risks to the selected patients, the CRF can be modified after the Research Committee adopt it through discussion at the meeting. If the amendment of the CRF requires no changes to this study protocol, the latter will not be modified.

11.2 Monitoring and Supervising

To assess whether study implementation follows protocol and data are being collected properly, monitoring should be conducted every February during the follow-up period. Monitoring is to complete through visiting a hospital and comparing the original Data.

11.2.1 Monitoring item

- Data Collection Completion Status: By selected registration numbers (cumulative and for each period)
- Eligibility: Not eligible patients/potentially ineligible patients

- Different end of treatment, the reasons for the suspension/end of the study protocol
- Background factors, pre-treatment report factors, post-treatment report factors when selected for registration
- Severe adverse events
- Adverse events/adverse reactions
- Laparoscopic surgery completion percentage
- Proportion of conversion to laparotomy
- Protocol deviation
- Disease-free survival /overall survival (all enrolled patients)
- Progress and safety of the study, other issues

11.2.2 Acceptable range of adverse events

Treatment-related death and life-threatening complications caused by surgeries occur relatively rarely and partly are dependent on the qualifications of the research participating hospitals and their staff; a rate of over 3% is considered unacceptable. If treatment-related death is suspected or non-hematologic Grade 4 toxicity having a causal relationship with the surgery is determined, adverse events should be reported to the Efficacy and Safety Evaluation Committee. If the number of treatment-related deaths or the number of patients with determined non-hematologic Grade 4 toxicity having a causal relationship with the surgery reached 9, the final incidence proportion of adverse events would be expected to exceed 3%, and therefore the inclusion of patients must be immediately suspended. Whether the study can continue should be determined by the Efficacy and Safety Evaluation Committee.

12 Relevant Provisions on adverse events

12.1 Surgery-related adverse events

See the adverse events mentioned for surgical complications in 8.3Definition of the study endpoint.

12.2 Various forms of adverse events caused by original incidence

Adverse events relating to various forms of deterioration in primary diseases should be recorded according to the short name of CTCAEv3.0.

12.3 Evaluation of adverse events

- Evaluation of adverse event/adverse reaction is based on [Accordion Severity Grading System] and [CTCAE v3.0].
- Adverse events will be graded 0 ~ 4 as per definition. For treatment-related death, fatal adverse events are classified as Grade 5 in the original CTCAE.
- Toxicity items specified in the [surgery-related adverse events], Grade, and the discovery date of Grade should be recorded in the treatment process report. For other

toxicity items observed, observed Grade 3 toxicity items are only recorded in the freedom registration column of the treatment process report, as well as Grade and the discovery date of Grade. The grade recorded in the treatment process report must be recorded in the case report form.

- CTCAE v3.0, the so-called “Adverse Event”, “all observed, unexpected bad signs, symptoms and diseases (abnormal value of clinical examination are also included) in the treatment or disposal, regardless of a causal relationship with the treatment or handling, including determining whether there is a causal relationship or not”.

- Therefore, even if events were “obviously caused by primary disease (cancer)” or caused by supportive therapy or combination therapy rather than the study regimen treatment (protocol treatment), they are “adverse events”.

- For adverse event data collection strategy, the following principles should be complied with in this study:1) Adverse events within 30 days from the last treatment day of the study regimen treatment (protocol treatment), regardless of the presence or absence of a causal relationship should be completely collected. (When adverse events are reported, the causality and classification of adverse events are separately discussed)2) Adverse events within 30 days from the last treatment day of the study regimen treatment (protocol treatment), regardless of the presence or absence of a causal relationship should be completely collected. (When adverse events are reported, the causality and classification of adverse events are separately discussed).

12.4 Reporting of Adverse Events

- When “severe adverse events” or “unexpected adverse events” occur, the Research Responsible Person of the research participating unit should report them to the Research Committee (Chang-Ming Huang).
- Based on the relevant laws and regulations, adverse events should be reported to the province (city) Health Department at the location of the research center. Severe adverse events based on clinical research-related ethical guidelines should be reported to the person in overall charge of the medical institution. The appropriate reporting procedures should be completed in accordance with the relevant provisions of all medical institutions at the same time. The person in charge of the research should hold accountability and responsibility for the emergency treatment of patients with any degree of adverse events to ensure patient safety.

2) Adverse events within 30 days from the last treatment day of the study regimen treatment (protocol treatment), regardless of the presence or absence of a causal

relationship should be completely collected. (When adverse events are reported, the causality and classification of adverse events are separately discussed).

12.4.1 Adverse Events with Reporting Obligations

12.4.1.1 Adverse Events with Emergency Reporting Obligations

Any of the following adverse events should be reported on an emergent basis:

- All patients who die during the course of treatment or within 30 days from the last treatment day, regardless of the presence or absence of a causal relationship with the study regimen treatment. Also, cases of discontinuation of treatment, even if within 30 days from the last treatment day, those patients are also emergent reporting objects. ("30 days" refers to day 0, the final treatment day, 30 days starting from the next day).
- Those patients with unexpected Grade 4 non-hematologic toxicity (CTCAE v3.0 adverse events other than the blood/bone marrow group), having a causality of treatment (any of definite, probable, possible) who emergent reporting objects are.

12.4.1.2 Adverse Events with Regular Reporting Obligations

One of the following adverse events are regular reporting objects:

(1) After 31 days from the last treatment day, deaths for which a causal relationship with treatment cannot be denied, including suspected treatment-related death; death due to obvious primary disease is included.

(2) Expected Grade 4 non-hematologic toxicity (CTCAE v3.0 adverse events other than the blood/bone marrow group).

(3) Unexpected Grade 3adverse events: Grade 3 adverse events are not recorded in the 12.1 expected adverse events.

(4) Other significant medical events: adverse events that the study group deems cause important and potentially permanent, significant impact on their offspring (MDS myelodysplastic syndrome, except for secondary cancer) Adverse events among above (2)-(4), determined to have a causal relationship (any of definite, probable, possible) with the study regimen is regular reporting objects.

12.4.2 Reporting Procedure

12.4.2.1 Emergency Reporting

- In case of any adverse event on emergency study reporting objects, the doctor in charge will quickly report it to the Research Responsible Person of the research participating hospitals. When the Research Responsible Person of the hospital cannot be contacted, the coordinator or the doctor in charge of the hospital must assume the responsibility on behalf of the Research Responsible Person of the hospital.
- First Reporting: Within 72 hours after the occurrence of adverse events, the

Research Responsible Person of the hospital should complete the "AE/AR/ADR first emergency report" and send it to the Research Committee by email and telephone.

- Second Reporting: The Research Responsible Person of the research participating hospital completes the "AE/AR/ADR Report" and a more detailed case information report (A4 format) and then faxes the two reports to the Research Committee within 15 days after the occurrence of adverse events. If any autopsy examination, the autopsy result report should be submitted to the Research Committee.

12.4.2.2 General Reports

- The Research Responsible Person of the research participating hospital completes the "AE/AR/ADR report", and then faxes it to the Research Committee within 15 days after the occurrence of adverse events.

12.5 Review of Efficacy and Safety Evaluation Committee

The Efficacy and Safety Evaluation Committee reviews and discusses the report in accordance with the procedures recorded in the *Clinical Safety Information Management Guideline*, and makes recommendations in writing for the Research Responsible Person, including whether to continue to include study objects or to modify the study protocol.

13 Ethical Considerations

13.1 Responsibilities of researchers

The investigators are responsible for the conduction of this study. The investigators will ensure the implementation of this study in accordance with the study protocol and in compliance with the Declaration of Helsinki, as well as domestic and international ethical guiding principles and applicable regulatory requirements. It is especially noted that the investigators must ensure that only subjects providing informed consent can be enrolled in this study.

13.2 Information and Informed Consent of Subjects

An unconditional prerequisite for subjects to participate in this study is his/her written informed consent. The written informed consent of subjects participating in this study must be given before study-related activities are conducted. Therefore, before obtaining informed consent, the investigators must provide sufficient information to the subjects. In order to obtain the informed consent, the investigators will provide the information page to subjects, and the information required to comply with the applicable regulatory requirements. While providing written information, the investigators will orally inform the subjects of all the relevant circumstances of this study. In this process, the information must be fully and easily understood by non-professionals, so that they can sign the informed consent form according to their

own will on the basis of their full understanding of this study.

The informed consent form must be signed and dated personally by the subjects and investigators. All subjects will be asked to sign the informed consent form to prove that they agree to participate in the study. The signed informed consent form should be kept at the research center and must be properly safe kept for future review at any time during audit and inspection throughout the inspection period. Before participating in the study, the subjects should provide a copy of the signed and dated informed consent form.

At any time, if important new information becomes available that may be related to the consent of the subjects, the investigators will revise the information pages and any other written information which must be submitted to the IEC/IRB for review and approval. The revised information approved will be provided to each subject participating in the study. The researchers will explain the changes made to the previous version of ICF to the subjects.

13.3 Identity and Privacy of Subjects

After obtaining an informed consent form, each selected subject is assigned a subject number (Allocation Number). This number will represent the identity of the subject during the entire study and for the clinical research database of the study. The collected data of subjects in the study will be stored in the ID.

Throughout the entire study, several measures will be taken to minimize any breaches of personal information, including 1) only the investigators will be able to link to the research data of the subjects to themselves through the identity table kept at the research center after authorization; 2) during onsite auditing of raw data by the supervisors of this study, as well as relevant inspection and inspection visits by the supervision departments, the person engaging in the above activities may view the original medical information of subjects that will be kept strictly confidential.

Collection, transmission, handling, and storage of data on study subjects will comply with the data protection and privacy regulations. This information will be provided to the study subjects when their informed consent is being obtained for treatment procedures in accordance with national regulations.

13.4 Independent Ethics Committee or Institutional Review Committee

Before beginning the study, the research center will be responsible for submitting the study protocol and relevant documents (informed consent form, subject information page, CRF, and other documents that may be required) to the Independent Ethics Committee (IEC)/ Institutional Review Board (IRB) to obtain their favorable opinion/approval. The favorable opinions/approval documents of the IEC/IRB will be archived in the research center folders of the investigators.

Before beginning the study at the center, the investigators must obtain written proof of favorable opinions/approval by the IEC/IRB and should provide written proof

of the date of the favorable opinions/approval meeting, written proof of the members presenting at the meeting, and voting members, written proof of recording the reviewed study, protocol version, and Informed Consent Form version, and if possible, a copy of the minutes.

In the case of major revisions to this study, the amendment of the study protocol will be submitted to the IEC/IRB prior to performing the study. In the course of the study, the relevant safety information will be submitted to the IEC/IRB in accordance with national regulations and requirements.

13.5 Supervising

The research approach of the authorities and any associated files (such as the research protocol, subjects' informed consent) will be in accordance with the requirements of the ethical review board of biomedical research involving humans (Trial) (2007) and the applicable Chinese laws and regulations. Studies should provide the main references or inform the ethics review guidance advisory organization of the provincial health administrative department in the province the research center is in.

14 Organizations and Responsibilities of Study

14.1 Research Committee

- Responsible for developing study protocol, auditing eligibility for inclusion, and guiding the interpretation of informed consent; also responsible for the collection of adverse event reports, guiding the clinical diagnosis and treatment of such events, and the emergency intervention of serious adverse events.
- Person in Charge of Research Committee: Changming Huang (Department of Gastric Surgery, Fujian Medical University Union Hospital)
  Add: Department of Gastric Surgery, Fujian Medical University Union Hospital, No.29 Xinquan Road, Fuzhou 350001, Fujian Province, China.; Post code: 350001; Tel: 0591-83357896-8011； Fax: 0591-83363366； Mobile: +8613805069676; E-mail:hcmlr2002@163.com
  Central Contact Backup: Qi-Yue Chen, PhD, Telephone: +8615980235636. Email: 690934662@qq.com
- Chief Statistical Expert of Research Committee: Zhijian Hu (Department of Preventive Medicine statistics, School of Public health, Fujian Medical University)

14.2 Efficacy and Safety Evaluation Committee

Responsible for the supervision/monitoring of treatment safety and efficacy of this study.

- Person in Charge of Efficacy and Safety Evaluation Committee: Changming Huang (Department of Gastric Surgery, Fujian Medical University Union Hospital)

14.3 Independent Ethics Committee/Institutional Review Board (IEC/IRB)

Responsible for evaluating this study to determine if risks to which subjects are exposed have been duly minimized and whether these risks are reasonable compared to expected benefits.

15 References

1. Kitano S, Iso Y, Moriyama M, Sugimachi K. Laparoscopy-assisted Billroth I gastrectomy. *Surgical laparoscopy & endoscopy.* 1994;4(2):146-148.
2. Yu J, Huang C, Sun Y, et al. Effect of Laparoscopic vs Open Distal Gastrectomy on 3-Year Disease-Free Survival in Patients With Locally Advanced Gastric Cancer: The CLASS-01 Randomized Clinical Trial. *Jama.* 2019;321(20):1983-1992.
3. Kim HH, Han SU, Kim MC, et al. Effect of Laparoscopic Distal Gastrectomy vs Open Distal Gastrectomy on Long-term Survival Among Patients With Stage I Gastric Cancer: The KLASS-01 Randomized Clinical Trial. *JAMA oncology.* 2019.
4. Bandoh T, Shiraishi N, Yamashita Y, et al. Endoscopic surgery in Japan: The 12th national survey(2012–2013) by the Japan Society for Endoscopic Surgery. *Asian Journal of Endoscopic Surgery.* 2017;10(4).
5. Vahrmeijer AL, Hutteman M, van der Vorst JR, van de Velde CJ, Frangioni JV. Image-guided cancer surgery using near-infrared fluorescence. *Nature reviews Clinical oncology.* 2013;10(9):507-518.
6. Valente SA, Al-Hilli Z, Radford DM, Yanda C, Tu C, Grobmyer SR. Near Infrared Fluorescent LN Mapping with Indocyanine Green in Breast Cancer Patients: A Prospective Trial. *Journal of the American College of Surgeons.* 2019;228(4):672-678.
7. Yamashita S, Tokuishi K, Anami K, et al. Video-assisted thoracoscopic indocyanine green fluorescence imaging system shows sentinel LNs in non-small-cell lung cancer. *The Journal of thoracic and cardiovascular surgery.* 2011;141(1):141-144.
8. Brouwer OR, Klop WM, Buckle T, et al. Feasibility of sentinel node biopsy in head and neck melanoma using a hybrid radioactive and fluorescent tracer. *Ann Surg Oncol.* 2012;19(6):1988-1994.
9. Gioux S, Choi HS, Frangioni JV. Image-guided surgery using invisible near-infrared light: fundamentals of clinical translation. *Molecular imaging.* 2010;9(5):237-255.
10. Schaafsma BE, Mieog JS, Hutteman M, et al. The clinical use of indocyanine green as a near-infrared fluorescent contrast agent for image-guided oncologic surgery. *J Surg Oncol.* 2011;104(3):323-332.
11. Desiderio J, Trastulli S, Gemini A, et al. Fluorescence image-guided lymphadenectomy using indocyanine green and near infrared technology in robotic gastrectomy. *Chinese journal of*

cancer research = Chung-kuo yen cheng yen chiu. 2018;30(5):568-570.

12. Liu M, Xing J, Xu K, et al. Application of Near-Infrared Fluorescence Imaging with Indocyanine Green in Totally Laparoscopic Distal Gastrectomy. *Journal of gastric cancer.* 2020;20(3):290-299.
13. Roh CK, Choi S, Seo WJ, et al. Indocyanine green fluorescence lymphography during gastrectomy after initial endoscopic submucosal dissection for early gastric cancer. *The British journal of surgery.* 2020.
14. Cianchi F, Indennitate G, Paoli B, et al. The Clinical Value of Fluorescent Lymphography with Indocyanine Green During Robotic Surgery for Gastric Cancer: a Matched Cohort Study. *Journal of gastrointestinal surgery : official journal of the Society for Surgery of the Alimentary Tract.* 2019.
15. Herrera-Almario G, Patane M, Sarkaria I, Strong VE. Initial report of near-infrared fluorescence imaging as an intraoperative adjunct for LN harvesting during robot-assisted laparoscopic gastrectomy. *J Surg Oncol.* 2016;113(7):768-770.
16. Baiocchi GL, Molfino S, Molteni B, et al. Fluorescence-guided lymphadenectomy in gastric cancer: a prospective western series. *Updates in surgery.* 2020;72(3):761-772.
17. Association JGC. Japanese classification of gastric carcinoma: 3rd English edition. *Gastric Cancer.* 2011;14(2):101.
18. Wilson MR, Poolton JM, Malhotra N, Ngo K, Bright E, Masters RS. Development and validation of a surgical workload measure: the surgery task load index (SURG-TLX). *World J Surg.* 2011;35(9):1961-1969.
19. Brédart A, Bottomley A, Blazeby JM, et al. An international prospective study of the EORTC cancer in-patient satisfaction with care measure (EORTC IN-PATSAT32). *European journal of cancer (Oxford, England : 1990).* 2005;41(14):2120-2131.
20. Huang CM, Zheng CH. *Laparoscopic Gastrectomy for Gastric Cancer.* Springer; 2015.
21. Huang CM, Chen QY, Lin JX, et al. Laparoscopic Suprapancreatic Lymph Node Dissection for Advanced Gastric Cancer Using a Left-Sided Approach. *Ann Surg Oncol.* 2015;22(7):2351.
22. Huang CM, Zhang JR, Zheng CH, et al. A 346 Case Analysis for Laparoscopic Spleen-Preserving No.10 lymph node Dissection for Proximal Gastric Cancer: A Single Center Study. *Plos One.* 2014;9(9):e108480.
23. Chen QY, Huang CM, Zheng CH, et al. Strategies of laparoscopic spleen-preserving splenic hilar LN dissection for advanced proximal gastric cancer. *World journal of gastrointestinal surgery.* 2016;8(6):402-406.
24. Maezawa Y, Aoyama T, Yamada T, et al. Priority of LN dissection for proximal gastric cancer

invading the greater curvature. *Gastric Cancer.* 2018;21(3):569-572.

25. Zhong Q, Chen QY, Xu YC, et al. Reappraise role of No. 10 lymphadenectomy for proximal gastric cancer in the era of minimal invasive surgery during total gastrectomy: a pooled analysis of 4 prospective trial. *Gastric Cancer.* 2020.

26. Dindo D, Demartines N, Clavien PA. Classification of surgical complications: a new proposal with evaluation in a cohort of 6336 patients and results of a survey. *Ann Surg.* 2004;240(2):205-213.
