# Supplementary material for: Comparison of submucosal and subserosal approaches toward optimized indocyanine green tracer-guided laparoscopic lymphadenectomy for patients with gastric cancer (FUGES-019): a randomized controlled trial
Source: BMC Med. 2021 Oct 27;19:276. doi: 10.1186/s12916-021-02125-y (PMC8549272; doi:10.1186/s12916-021-02125-y)
Supplement: Supplementary file 2 — Additional file 2: Table S1-Table S13. [file 12916_2021_2125_MOESM2_ESM.pdf]

Supplemental Content 2. eTables

eTable 1. Eligibility Criteria for Enrolling Patients

eTable 2. Checklist for Determining the Success of D2 Lymphadenectomy

eTable 3. The modified EORTC IN-PATSAT14 Questionnaire

eTable 4. Basic Characteristics of Patients in the SMA and SSA Groups Who Underwent Distal Gastrectomy and Total Gastrectomy

eTable 5. Number of Retrieved Lymph Nodes in the SMA and SSA Groups According to the D Station

eTable 6. Extent of Lymph Node Compliance in the SMA and SSA Groups

eTable 7. Number of Metastatic Lymph Nodes in the SMA and SSA Groups

eTable 8. Validity of ICG That Emitted Fluorescence and Lymph Node Metastasis

eTable 9. Diagnostic Value of Fluorescent Lymphography for the Detection of Metastatic Stations According to the Total Number of Stations

eTable 10. Surgical Outcomes, Postoperative Recovery, Morbidity, and Mortality in Patients in the SMA and SSA Groups Who Underwent Distal Gastrectomy or Total Gastrectomy

eTable 11. Cost Difference Between the SMA and SSA Groups

eTable 12. Descriptive Statistics of In-patient Satisfaction Scales

eTable 13. Comparison of the Mean Surg-TLX Subscales Between the SMA and SSA Groups

eTable 1. Eligibility Criteria for Enrolling Patients

Inclusion criteria:

1. Age from 18 to 75 years
2. Primary gastric adenocarcinoma (papillary, tubular, mucinous, signet ring cell, or poorly differentiated) confirmed pathologically by endoscopic biopsy
3. A tumor stage of T1-4a (cT1-4a), N0/+, M0 at preoperative evaluation according to the American Joint Committee on Cancer (AJCC) Cancer Staging Manual Eighth Edition. Preoperative staging was made by conducting mandatory computed tomography (CT) scans and an optional endoscopic ultrasound
4. No distant metastasis, no direct invasion of pancreas, spleen, or other organs nearby in the preoperative examinations
5. Performance status of 0 or 1 on Eastern Cooperative Oncology Group scale (ECOG)
6. American Society of Anesthesiology score (ASA) class I, II, or III
7. Written informed consent

Exclusion criteria:

1. Women during pregnancy or breast-feeding
2. Severe mental disorder
3. History of previous upper abdominal surgery (except laparoscopic cholecystectomy)
4. History of previous gastrectomy, endoscopic mucosal resection, or endoscopic submucosal dissection
5. Rejection of laparoscopic resection
6. History of allergy to iodine agents
7. Enlarged or bulky regional lymph node diameter over 3cm by preoperative imaging
8. History of other malignant diseases within the past five years
9. History of previous neoadjuvant chemotherapy or radiotherapy
10. History of unstable angina or myocardial infarction within the past six months
11. History of a cerebrovascular accident within the past six months
12. History of continuous systematic administration of corticosteroids within one month
13. Requirement of simultaneous surgery for other diseases
14. Emergency surgery due to complication (bleeding, obstruction, or perforation) caused by gastric cancer
15. Forced expiratory volume in 1 second (FEV1) < 50% of predicted values
16. Linitis plastica, Widespread

eTable 2. Checklist for Determining the Success of D2 Lymphadenectomy

Scoring Method for D2 Lymph Node Dissection	Complete<br>10	Incomplete<br>5	None<br>0
1. Properly full omentectomy	□	□	□
2. Ligation of the left gastroepiploic artery at origin	□	□	□
3. Ligation of the right gastroepiploic artery at origin	□	□	□
4. Full exposure of the common hepatic artery	□	□	□
5. Ligation of the right gastric artery at origin	□	□	□
6. Exposure of the portal vein	□	□	□
7. Exposure of the splenic artery	□	□	□
8. Identification of the splenic vein	□	□	□
9. Ligation of the left gastric artery at origin	□	□	□
10. Exposure of the gastroesophageal junction	□	□	□

1. Properly full omentectomy
    a. Omentectomy was performed close to the transverse colon
    b. Omentectomy was performed from hepatic flexure to splenic flexure
    c. The anterior layer of the transverse colonic mesentery and anterior pancreatic peritoneum were dissected.
2. Ligation of the left gastroepiploic artery at origin
3. Ligation of the right gastroepiploic artery at origin
4. Full exposure of common hepatic artery
    a. More than half of the anterior part of the common hepatic artery was exposed.
5. Ligation of the right gastric artery at origin
6. Exposure of the portal vein
7. Exposure of the splenic artery
    a. The anterior part in the splenic artery was exposed
    b. The splenic artery was exposed from the celiac trunk to the posterior gastric artery (distal gastrectomy)
    c. The splenic artery was exposed from the celiac trunk to the terminal branch of the splenic artery (total gastrectomy)
8. Identification of the splenic vein
9. Ligation of the left gastric artery at origin
10. Exposure of the gastroesophageal junction

a. The anterior and right side of the abdominal esophagus were exposed (distal gastrectomy)

b. The anterior, posterior, left, and right sides of the abdominal esophagus were exposed (total gastrectomy)

- D2 lymphadenectomy was accepted if all three randomly assigned investigators rated ≥85 points regarding checklists in the unedited video review.

eTable 3. The modified EORTC IN-PATSAT14 Questionnaire

We are interested in your experience of the care received during your hospital stay. Please answer all the questions yourself by circling the number that best applies to you. There are no "right" or "wrong" answers. The information that you provide will remain strictly confidential. Thank you very much.

	During your hospital stay, how would you rate **doctors**, in terms of	Poor	Fair	Good	Very good	Excellent
1	Their knowledge and experience of your illness?	1	2	3	4	5
2	The treatment and medical follow-up they provided?	1	2	3	4	5
3	Their willingness to listen to all of your concerns?	1	2	3	4	5
4	The information they gave you about your illness, medical tests, and treatment?	1	2	3	4	5
5	The frequency of their visits/consultations?	1	2	3	4	5
6	The time they devoted to you during visits/consultations?	1	2	3	4	5
	During your hospital stay, how would you rate **services** in terms of	Poor	Fair	Good	Very good	Excellent
7	The information provided on your admission to the hospital?	1	2	3	4	5
8	The information provided on your discharge from the hospital?	1	2	3	4	5
9	The kindness and helpfulness of the technical, reception, laboratory personnel?	1	2	3	4	5
10	The waiting time for obtaining results of medical tests?	1	2	3	4	5
11	The speed of implementing medical tests and/or treatments?	1	2	3	4	5
	**Special item**	Quite a lot	Serious	Moderate	Middle	Not at all
12	Was any discomfort caused by the examination or treatment during the hospitalization period?	1	2	3	4	5
13	Is there any examination or treatment that you think is repeated and bother you?	1	2	3	4	5
	**Overall**	Poor	Fair	Good	Very good	Excellent
14	How would you rate the care received during your hospital stay?	1	2	3	4	5

The modified EORTC IN-PATSAT14 contains five multi-item and three single-item scales. These include the doctors' technical skills (items 1 and 2), interpersonal skills (item 3), information provision (item 4), availability (items 5 and 6) scales; other hospital staff interpersonal skills and information provision scale (items 7–9); waiting time scale (items 10 and 11); comfort special-item scale (items 12 and 13); and general satisfaction single-item scale (item 14).

eTable 4. Basic Characteristics of Patients in the SMA and SSA Groups Who Underwent Distal Gastrectomy and Total Gastrectomy

	Distal gastrectomy			Total gastrectomy		
	Mean (SD) / No. (%)			Mean (SD) / No. (%)		
Characteristic	SMA (n=71)	SSA (n=65)	*P* Value	SMA (n=59)	SSA (n=64)	*P* Value
**Age, y**	57.0 (12.2)	58.3 (9.6)	0.517	60.9 (9.7)	59.7 (11.0)	0.526
**BMI, kg/m^2**	21.8 (3.3)	22.2 (2.8)	0.433	23.3 (3.0)	22.5 (3.3)	0.156
**Sex**			0.612			0.752
Male	44 (62.0)	43 (66.2)		43 (72.9)	45 (70.3)	
Female	27 (38.0)	22 (33.8)		16 (27.1)	19 (29.7)	
**ECOG performance status**			0.832			0.972
0	58 (81.7)	54 (83.1)		49 (83.1)	53 (82.8)	
1	13 (18.3)	11 (16.9)		10 (16.9)	11 (17.2)	
**Tumor location**			0.444			0.181
Upper	0 (0.0)	0 (0.0)		27 (45.8)	21 (32.8)	
Middle	5 (7.0)	7 (10.8)		26 (44.1)	30 (46.9)	
Lower	66 (93.0)	58 (89.2)		6 (10.2)	13 (20.3)	
**Reconstruction**			0.121			-
Billroth I	5 (7.0)	10 (15.4)		0 (0.0)	0 (0.0)	
Billroth II	66 (93.0)	55 (84.6)		0 (0.0)	0 (0.0)	
Roux-en-Y	0 (0.0)	0 (0.0)		59(100.0)	64(100.0)	
**Histology**			0.246			0.459
Differentiated	29 (40.8)	33 (50.8)		26 (44.1)	24 (37.5)	
Undifferentiated	42 (59.2)	32 (49.2)		33 (55.9)	40 (62.5)	
**Lymphvascular invasion**			0.881			0.255
Negative	45 (63.4)	42 (64.6)		29 (49.2)	38 (59.4)	
Positive	26 (36.6)	23 (35.4)		30 (50.8)	26 (40.6)	
**Size, cm**			0.597			0.260
≤4	45 (63.4)	44 (67.7)		30 (50.8)	39 (60.9)	
>4	26 (36.6)	21 (32.3)		29 (49.2)	25 (39.1)	
**cT category**			0.642			0.233
cT1	30 (42.3)	24 (36.9)		11 (18.6)	22 (34.4)	
cT2	12 (16.9)	8 (12.3)		7 (11.9)	8 (12.5)	
cT3	15 (21.1)	19 (29.2)		29 (49.2)	23 (35.9)	
cT4a	14 (19.7)	14 (21.5)		12 (20.3)	11 (17.2)	
**cN category**			0.982			0.242
cN0	34 (47.9)	31 (47.7)		18 (30.5)	26 (40.6)	
cN+	37 (52.1)	34 (52.3)		41 (69.5)	38 (59.4)	
**pT category**			0.819			0.070
pT1	32 (45.1)	27 (41.5)		7 (11.9)	20 (31.3)	
pT2	9 (12.7)	6 (9.2)		10 (16.9)	7 (10.9)	
pT3	19 (26.8)	19 (29.2)		29 (49.2)	24 (37.5)	
pT4a	11 (15.5)	13 (20.0)		13 (22.0)	13 (20.3)	

**pN category**			0.949			0.545
pN0	35 (49.3)	31 (47.7)		17 (28.8)	27 (42.2)	
pN1	12 (16.9)	14 (21.5)		15 (25.4)	10 (15.6)	
pN2	11 (15.5)	8 (12.3)		12 (20.3)	12 (18.8)	
pN3a	8 (11.3)	8 (12.3)		12 (20.3)	12 (18.8)	
pN3b	5 (7.0)	4 (6.2)		3 (5.1)	3 (4.7)	
**AJCC 8th pTNM staging**			0.231			0.091
I	35 (49.3)	26 (40.0)		11 (18.6)	23 (35.9)	
II	13 (18.3)	20 (30.8)		21 (35.6)	16 (25.0)	
III	23 (32.4)	19 (29.2)		27 (45.8)	25 (39.1)	
**CEA, ng/ml**			0.237			0.997
<5	63 (88.7)	53 (81.5)		47 (79.7)	51 (79.7)	
≥5	8 (11.3)	12 (18.5)		12 (20.3)	13 (20.3)	
**CA19-9, U/ml**			0.650			0.239
<37	63 (88.7)	56 (86.2)		47 (79.7)	56 (87.5)	
≥37	8 (11.3)	9 (13.8)		12 (20.3)	8 (12.5)	
**CA72-4, U/ml**			0.611			0.717
<6.9	68 (95.8)	61 (93.8)		52 (88.1)	55 (85.9)	
≥6.9	3 (4.2)	4 (6.2)		7 (11.9)	9 (14.1)	
**AFP, ng/ml**			0.950			0.350
<20	70 (98.6)	64 (98.5)		58 (98.3)	61 (95.3)	
≥20	1 (1.4)	1 (1.5)		1 (1.7)	3 (4.7)	
**CA125, U/ml**			0.950			0.934
<35	70 (98.6)	64 (98.5)		57 (96.6)	62 (96.9)	
≥35	1 (1.4)	1 (1.5)		2 (3.4)	2 (3.1)	

Abbreviations: AJCC, American Joint Committee on Cancer; BMI, body mass index (calculated as weight in kilograms divided by height in meters squared); ECOG, Eastern Cooperative Oncology; LN, lymph node; CEA, Carcinoembryonic antigen; CA199, Carbohydrate antigen 19-9. CA72-4, Carbohydrate antigen 72-4; AFP, Alpha fetoprotein; CA125, Carbohydrate antigen 125; SMA, submucosa approach; SSA, subserosa approach; SD, standard deviation.

eTable 5. Number of Retrieved Lymph Nodes in the SMA and SSA Groups According to the D Station

	All patients			Total gastrectomy			Distal gastrectomy		
	Mean. (SD)			Mean (SD)			Mean (SD)		
Variable	SMA (n=130)	SSA (n=129)	*P Value*	SMA (n=59)	SSA (n=64)	*P Value*	SMA (n=71)	SSA (n=65)	*P Value*
Metastatic lymph nodes of D1 station	3.3 (5.5)	2.9 (4.8)	0.569	3.8 (5.8)	3.3 (5.2)	0.393	2.8 (5.2)	2.5 (4.4)	0.989
Number of D1 station lymph nodes retrieved	32.8 (12.0)	32.5 (11.5)	0.860	35.4 (13.7)	34.3 (12.2)	0.960	30.6 (10.0)	30.7 (10.5)	0.889
Metastatic lymph nodes of D1+ station	0.5 (1.3)	0.6 (2.2)	0.334	0.6 (1.0)	0.8 (2.9)	0.267	0.5 (1.5)	0.4 (0.9)	0.740
Number of D1+ station lymph nodes retrieved	10.5 (5.3)	10.6 (5.0)	0.667	11.1 (5.3)	11.3 (5.3)	0.770	9.9 (5.3)	9.9 (4.6)	0.836
Metastatic lymph nodes of D2 station	0.3 (0.8)	0.2 (0.8)	0.724	0.1 (0.4)	0.1 (0.4)	0.883	0.4 (1.0)	0.3 (1.0)	0.807
Number of D2 station lymph nodes retrieved	5.9 (4.8)	5.0 (3.8)	0.281	3.3 (2.9)	3.6 (2.8)	0.509	8.0 (5.0)	6.4 (4.1)	0.056

D1 stations (No. 1, 2, 3, 4sb, 4d, 4sa, 5, 6, and 7 for total gastrectomy; No. 1, 3, 4sb, 4d, 5, 6, and 7 for distal gastrectomy)

D1+ stations (No. 8a, 9 and 11p for total gastrectomy; No. 8a and 9 for distal gastrectomy)

D2 stations (No. 11d and 12a for total gastrectomy; No. 11p and 12a for distal gastrectomy)

eTable 6. Extent of Lymph Node Compliance in the SMA and SSA Groups

	No. (%)		
**Variable**	**SMA**	**SSA**	***P Value***
**All patients**			
Compliance (≤1)[a]	88 (67.7)	86 (66.7)	
Non-compliance (≥2)[a]	42 (32.3)	43 (33.3)	0.860
Minor non-compliance (2)[a]	24 (18.5)	20 (15.5)	0.640
Major non-compliance (≥3)[a]	18 (13.8)	23 (17.8)	0.380
3	9 (6.9)	17 (13.2)	0.142
≥4	9 (6.9)	6 (4.7)	0.605
**Distal gastrectomy**			
Compliance (≤1)[a]	52 (73.2)	47 (72.3)	
Non-compliance (≥2)[a]	19 (26.8)	18 (27.7)	0.903
Minor non-compliance (2)[a]	14 (19.7)	11 (16.9)	0.842
Major non-compliance (≥3)[a]	5 (7.0)	7 (10.8)	0.643
3	4 (5.6)	5 (7.7)	0.891
≥4	1 (1.4)	2 (3.1)	0.938
**Total gastrectomy**			
Compliance (≤1)[a]	36 (61.0)	39 (60.9)	
Non-compliance (≥2)[a]	23 (39.0)	25 (39.1)	0.993
Minor non-compliance (2)[a]	10 (16.9)	9 (14.1)	0.847
Major non-compliance (≥3)[a]	13 (22.0)	16 (25.0)	0.699
3	5 (8.5)	12 (18.8)	0.165
≥4	8 (13.6)	4 (6.3)	0.289

[a] Number of nodes not removed. SMA, submucosa approach; SSA, subserosa approach.

eTable 7. Number of Metastatic Lymph Nodes in the SMA and SSA Groups

	All patients			Total gastrectomy			Distal gastrectomy		
	Mean (SD)			Mean (SD)			Mean (SD)		
Metastatic LNs	SMA (n=130)	SSA (n=129)	*P Value*	SMA (n=59)	SSA (n=64)	*P Value*	SMA (n=71)	SSA (n=65)	*P Value*
Total	4.1 (6.8)	3.8 (6.8)	0.709	4.6 (6.6)	4.3 (8.0)	0.832	3.7 (7.0)	3.3 (5.5)	0.695
NO.1	0.2 (0.5)	0.1 (0.3)	0.070	0.1 (0.3)	0.1 (0.3)	0.912	0.2 (0.6)	0.0 (0.2)	0.057
NO.2	0.0 (0.2)	0.1 (0.6)	0.369	0.1 (0.4)	0.2 (0.9)	0.425	0.0 (0.0)	0.0 (0.0)	NA
NO.3	1.4 (2.7)	1.2 (2.2)	0.453	2.2 (3.4)	1.5 (2.3)	0.146	0.7 (1.8)	0.9 (2.1)	0.626
NO.4	0.7 (1.6)	0.7 (1.8)	0.961	0.6 (1.4)	1.0 (2.3)	0.310	0.7 (1.7)	0.4 (0.9)	0.131
NO.4sa	0.0 (0.2)	0.0 (0.1)	0.416	0.1 (0.3)	0.0 (0.2)	0.351	0.0 (0.0)	0.0 (0.0)	NA
NO.4sb	0.1 (0.2)	0.1 (0.3)	0.990	0.1 (0.3)	0.1 (0.5)	0.754	0.0 (0.2)	0.0 (0.0)	0.175
NO.4d	0.6 (1.5)	0.6 (1.5)	0.980	0.5 (1.2)	0.8 (2.0)	0.213	0.7 (1.7)	0.4 (0.9)	0.152
NO.5	0.2 (0.7)	0.2 (0.6)	0.522	0.1 (0.3)	0.1 (0.4)	0.700	0.3 (0.9)	0.3 (0.7)	0.662
NO.6	0.5 (1.6)	0.5 (1.4)	0.949	0.5 (1.9)	0.3 (1.1)	0.519	0.6 (1.3)	0.8 (1.6)	0.402
NO.7	0.2 (0.7)	0.2 (0.5)	0.562	0.2 (0.6)	0.1 (0.5)	0.906	0.2 (0.8)	0.2 (0.5)	0.549
NO.8a	0.2 (0.6)	0.2 (1.2)	0.488	0.2 (0.5)	0.3 (1.5)	0.598	0.2 (0.6)	0.2 (0.6)	0.673
NO.9	0.3 (1.0)	0.2 (0.9)	0.703	0.2 (0.6)	0.3 (1.1)	0.525	0.4 (1.2)	0.2 (0.5)	0.253
NO.11p	0.2 (0.6)	0.2 (0.7)	0.840	0.2 (0.5)	0.2 (0.6)	0.876	0.2 (0.7)	0.2 (0.8)	0.721
NO.11d	0.0 (0.2)	0.0 (0.2)	0.711	0.1 (0.3)	0.0 (0.2)	0.620	0.0 (0.0)	0.0 (0.0)	NA
NO.12a	0.1 (0.5)	0.1 (0.3)	0.453	0.1 (0.3)	0.1 (0.3)	0.845	0.2 (0.6)	0.1 (0.3)	0.358

Abbreviations: SMA, submucosa approach; SSA, subserosa approach; SD, standard deviation.

eTable 8. Validity of ICG That Emitted Fluorescence and Lymph Node Metastasis

	SMA			SSA		
**Variable**	**All patients**	**DG group**	**TG group**	**All patients**	**DG group**	**TG group**
**Sensitivity (true positive rate)**	62.2% (333/535)	64.3% (169/263)	60.3% (164/272)	58.0% (284/490)	56.8% (121/213)	58.8% (163/277)
**False negative rate**	37.8% (202/535)	35.7% (94/263)	39.7% (108/272)	42.0% (206/490)	43.2% (92/213)	41.2% (114/277)
**Specificity (true negative rate)**	48.5% (2878/5940)	45.5% (1468/3226)	52.0% (1410/2714)	41.2% (2408/5851)	41.2% (1177/2857)	41.1% (1231/2994)
**False positive rate**	51.5% (3062/5940)	54.5% (1758/3226)	48.0% (1304/2714)	58.8% (3443/5851)	58.8% (1680/2857)	58.9% (1763/2994)
**Positive likelihood ratio**	1.207	1.179	1.255	0.985	0.966	0.999
**Negative likelihood ratio**	0.779	0.785	0.764	1.022	1.048	1.001
**Youden index**	0.107	0.098	0.122	-0.009	-0.020	0.000
**Kappa value**	0.031	0.025	0.041	-0.002	-0.004	0.000

Abbreviations: ICG, Indocyanine green; LN, Lymph node; DG, Distal gastrectomy; TG, Total gastrectomy.

eTable 9. Diagnostic Value of Fluorescent Lymphography for the Detection of Metastatic Stations According to the Total Number of Stations

Variable	Total number of stations, n	Metastatic stations, n	Nonmetastatic stations, n	Sensitivity[a]	Specificity[b]	NPV[c]	PPV[d]
**SMA**							
All							
Fluorescent	1038	206 (TP)	832 (FP)	89.6%	31.2%	94.0%	19.8%
Non-fluorescent	402	24 (FN)	378 (TN)				
Distal gastrectomy							
Fluorescent	568	111 (TP)	457 (FP)	94.1%	24.2%	95.4%	19.5%
Non-fluorescent	153	7 (FN)	146 (TN)				
Total gastrectomy							
Fluorescent	470	95 (TP)	375 (FP)	84.8%	38.2%	93.2%	20.2%
Non-fluorescent	249	17 (FN)	232 (TN)				
**SSA**							
All							
Fluorescent	1129	192 (TP)	937 (FP)	90.1%	24.3%	93.5%	17.0%
Non-fluorescent	322	21 (FN)	301 (TN)				
Distal gastrectomy							
Fluorescent	519	87 (TP)	432 (FP)	87.9%	22.2%	91.1%	16.8%
Non-fluorescent	135	12 (FN)	123 (TN)				
Total gastrectomy							
Fluorescent	610	105 (TP)	505 (FP)	92.1%	26.1%	95.2%	17.2%
Non-fluorescent	187	9 (FN)	178 (TN)				

TP, true positive; FP, false positive; FN, false negative; TN, true negative;

[a] Sensitivity was calculated by [TP / (TP + FN)]

[b] Specificity was calculated by [TN / (TN + FP)]

[c] NPV, negative predictive value was calculated by [TN / (TN + FN)]

[d] PPV, positive predictive value was calculated by [TP / (TP + FP)]

eTable 10. Surgical Outcomes, Postoperative Recovery, Morbidity, and Mortality in Patients in the SMA and SSA Groups Who Underwent Distal Gastrectomy or Total Gastrectomy

Outcome	Distal gastrectomy Mean (SD) / No. (%) SMA (n=71)	SSA (n=65)	*P* Value	Total gastrectomy Mean (SD) / No. (%) SMA (n=59)	SSA (n=64)	*P* Value
**Surgical outcome**						
Estimated blood loss (mL)	47.3 (46.3)	47.7 (55.9)	0.960	55.8 (46.1)	57.3 (63.3)	0.882
Surgical time (minutes)	189.1 (40.7)	197.5 (51.0)	0.287	207.7 (45.4)	216.7 (51.8)	0.306
**Postoperative recovery**						
Time to first flatus (days)	3.1 (0.9)	3.3 (0.7)	0.109	3.2 (1.4)	3.3 (0.7)	0.634
Time to ambulation (days)	2.1 (0.5)	2.1 (0.5)	0.825	2.2 (0.7)	2.3 (1.5)	0.507
Time to first liquid intake (days)	3.5 (0.8)	3.6 (0.9)	0.163	3.7 (1.2)	3.9 (1.6)	0.520
Time to first semifluid intake (days)	5.1 (0.5)	5.7 (5.1)	0.307	6.0 (2.8)	5.7 (2.5)	0.489
Postoperative hospital stays (days)	7.1 (2.1)	7.6 (6.3)	0.537	9.7 (6.9)	8.9 (4.7)	0.457
Postoperative transfusion	4 (5.6)	3 (4.6)	0.788	5 (8.5)	4 (6.3)	0.636
Reoperation[a]	1 (1.4)	0 (0.0)	0.337	0 (0.0)	0 (0.0)	-
Unplanned readmission[b]	0 (0.0)	1 (1.5)	0.294	1 (1.7)	2 (3.1)	0.607
**Morbidity type**						
Postoperative complication	10 (14.1)	11 (16.9)	0.647	13 (22.0)	10 (15.6)	0.362
Clavien-Dindo classification			0.516			0.767
I	0 (0.0)	2 (3.1)		1 (1.7)	0 (0.0)	
II	8 (11.3)	7 (10.8)		9 (15.3)	8 (12.5)	
IIIa	1 (1.4)	1 (1.5)		2 (3.4)	1 (1.6)	
IIIb	1 (1.4)	0 (0.0)		0 (0.0)	0 (0.0)	
IV	0 (0.0)	1 (1.5)		1 (1.7)	1 (1.6)	
V	0 (0.0)	0 (0.0)		0 (0.0)	0 (0.0)	

Abbreviations: LN, lymph node; SMA, submucosa approach; SSA, subserosa approach; SD, standard deviation.

[a] Due to one case of postoperative intra-abdominal hemorrhage in the SMA group.

[b] Due to one case of anastomotic stenosis in the SSA group underwent distal gastrectomy, one case of postoperative abdominal infection and one case of postoperative ileus in the SSA group underwent total gastrectomy; one case of postoperative abdominal infection in the SMA group underwent total gastrectomy.

eTable 11. Cost Difference Between the SMA and SSA Groups

	All patients			Distal gastrectomy			Total gastrectomy		
	Mean (SD)			Mean (SD)			Mean (SD)		
Variable	SMA (n=130)	SSA (n=129)	*P Value*	SMA (n=71)	SSA (n=65)	*P Value*	SMA (n=59)	SSA (n=64)	*P Value*
**Total cost ($)**	9860.3 (970.0)	9640.6 (1276.2)	0.120	9440.8 (822.2)	9231.3 (790.3)	0.133	10365.2 (895.5)	10056.4 (1525.1)	0.178
**Total indirect cost ($)**[a]	935.7 (244.2)	925.1 (321.2)	0.765	818.7 (212.8)	800.1 (328.7)	0.693	1076.5 (202.7)	1052.1 (259.6)	0.564
**Total direct cost ($)**	8924.6 (913.5)	8715.5 (1180.6)	0.112	8622.1 (832.1)	8431.2 (739.6)	0.161	9288.7 (879.6)	9004.3 (1451.9)	0.196
Hospitalization ($)	252.9 (51.5)	244.9 (67.4)	0.284	231.3 (45.3)	222.7 (73.2)	0.406	278.8 (46.5)	267.4 (52.6)	0.206
Surgical cost ($)	1416.2 (346.9)	1396.7 (469.6)	0.704	1289.2 (308.7)	1254.3 (340.2)	0.532	1469.0 (330.3)	1441.2 (537.0)	0.733
Laboratory test ($)	426.4 (101.3)	413.5 (109.3)	0.325	378.5 (82.9)	364.3 (82.1)	0.320	484.2 (91.1)	463.5 (111.4)	0.265
Radiology test ($)	1092.0 (248.9)	1111.3 (248.4)	0.533	993.2 (231.0)	1005.6 (218.9)	0.749	1210.9 (216.9)	1218.6 (231.2)	0.849
Medication and injections ($)	2389.4 (569.3)	2474.5 (813.5)	0.330	2074.9 (447.0)	2152.4 (424.5)	0.303	2767.9 (461.5)	2801.6 (972.5)	0.809
Fluorescence related fees ($)[b]	335.3 (5.5)	182.4 (4.4)	<0.001	335.2 (6.1)	183.0 (4.8)	<0.001	335.4 (4.7)	181.9 (4.0)	<0.001
Others ($)	3012.4 (721.4)	2892.2 (835.2)	0.216	3319.8 (624.9)	3248.8 (695.0)	0.532	2642.5 (656.6)	2530.0 (813.6)	0.403

Exchange Rate Calculation 1 US dollar = 6.5751 RMB, Date 20/11/2020

Abbreviations: SMA, submucosa approach; SSA, subserosa approach; SD, Standard deviation.

[a] Including overhead cost of the building, the amortization of capital equipment, and supplies, maintenance of services, utilities and administrative staff.

[b] The fluorescence-related cost for the SMA included the cost of endoscopy, tracers, materials, and treatment, while that for the SSA included the cost of tracers, materials, and treatment.

eTable 12. Descriptive Statistics of In-patient Satisfaction Scales

	Mean. (SD)			n (%) floor		n (%) ceiling	
**Scales**	**SMA (n=130)**	**SSA (n=129)**	***P* Value**	**SMA (n=130)**	**SSA (n=129)**	**SMA (n=130)**	**SSA (n=129)**
SATDTS	76.8 (18.4)	76.4 (18.5)	0.884	1 (0.8)	1 (0.8)	30 (23.1)	31 (24.0)
SATDIS	78.5 (21.8)	79.4 (20.6)	0.728	4 (3.1)	2 (1.6)	47 (36.2)	50 (38.8)
SATDIP	70.5 (23.9)	69.8 (23.5)	0.814	2 (1.5)	1 (0.8)	37 (28.5)	34 (26.4)
SATDAV	67.7 (22.6)	67.4 (22.6)	0.907	4 (3.1)	4 (3.1)	21 (16.2)	20 (15.5)
SATOTH	67.5 (19.4)	68.5 (19.9)	0.701	1 (0.8)	0 (0.0)	19 (14.6)	19 (14.7)
SATWAI	65.4 (19.2)	66.8 (21.0)	0.566	3 (2.3)	2 (1.6)	8 (6.2)	15 (11.6)
SATCSI	63.2 (18.7)	72.8 (19.1)	<0.001	5 (3.8)	2 (1.6)	7 (5.4)	16 (12.4)
SATGEN	70.5 (24.3)	76.1 (21.5)	0.048	1 (0.8)	0 (0.0)	40 (30.8)	43 (33.3)

% floor = percentage of respondents at lowest scale rating. % ceiling = percentage of respondents at highest scale rating.

Modified EORTC IN-PATSAT14 scales (high score = high satisfaction rating): SATDTS = doctor's technical skills, SATDIS = doctor's interpersonal skills, SATDIP = doctor's information provision, SATDAV = doctor's availability, SATOTH = other personal interpersonal skills and information provision, SATWAI = waiting time, SATCSI = comfort special-item, SATGEN = overall quality rating.

Abbreviations: SMA, submucosa approach; SSA, subserosa approach; SD, Standard deviation.

eTable 13. Comparison of the Mean Surg-TLX Subscales Between the SMA and SSA Groups

	Mean (SD)		
**Variable**	**SMA (n=130)**	**SSA (n=129)**	***P Value***
Distractions	4.3 (1.4)	4.3 (1.4)	0.776
Situational Stress	4.8 (1.7)	4.7 (1.9)	0.910
Task Complexity	6.9 (2.9)	6.9 (2.8)	0.863
Temporal Demands	5.1 (1.9)	5.0 (1.9)	0.554
Physical Demands	8.5 (3.4)	8.6 (3.3)	0.791
Mental Demands	6.9 (3.4)	7.0 (3.4)	0.812
Total	36.4 (7.2)	36.6 (7.3)	0.861

Abbreviations: SMA, submucosa approach; SSA, subserosa approach; SD, Standard deviation.
