# Supplementary material for: Comparison of submucosal and subserosal approaches toward optimized indocyanine green tracer-guided laparoscopic lymphadenectomy for patients with gastric cancer (FUGES-019): a randomized controlled trial
Source: BMC Med. 2021 Oct 27;19:276. doi: 10.1186/s12916-021-02125-y (PMC8549272; doi:10.1186/s12916-021-02125-y)
Supplement: Supplementary file 3 — Additional file 3: Fig. S1-Fig. S9. [file 12916_2021_2125_MOESM3_ESM.pdf]

Supplemental Content 3. eFigures

eFigure 1. Illustration of Six Points for Subserosal Injection of ICG

A. Distal gastrectomy. B. Total gastrectomy.

eFigure 2. The Surg-TLX Questionnaire

eFigure 3. View of Lymph Nodes Under Near-Infrared Fluorescent Imaging After Subserosal Injection of ICG

A. Green fluorescence. B. Near-Infrared imaging. C. Color-Segmented fluorescence.

eFigure 4. Mean Number of Fluorescent Lymph Nodes, Nonfluorescent Lymph Nodes in Fluorescent Stations, and Lymph Nodes in Nonfluorescent Stations According to the Lymph Node Station in the SMA and SSA Groups

[a]P<.05, [b]P<.001.

eFigure 5. Mean Number of Fluorescent Lymph Nodes and Nonfluorescent Lymph Nodes in Fluorescent Stations According to the Lymph Node Station in the SMA and SSA Groups.

eFigure 6. Lymph Node Dissection Rate at Each Station in the SMA and SSA Groups During Distal and Total Gastrectomy with D2 Lymph Node Dissection

A. Distal gastrectomy with D2 lymph node dissection. B. Total gastrectomy with D2 lymph node dissection. C. Noncompliance with lymph node dissection in the SMA group is shown in dark blue text and that in the SSA group is shown in gray text. SMA, submucosa approach; SSA, subserosa approach.

eFigure 7. Laboratory Findings Estimated Before Surgery and on Postoperative Days 1, 3, and 5.

(A) White blood cell count, (B) hemoglobin level, (C) platelet count, (D) total bilirubin level, and (E) albumin level.

eFigure 8. Total Number of Retrieved Lymph Nodes in the SMA, SSA and Non-ICG Groups

SMA, submucosa approach; SSA, subserosa approach.

eFigure 9. The Lymphatic System in the Gastric Wall. A, submucous plexus; B, lymphatic vessels among the muscular fibers; C, subserous plexus. LYVE1 indicates lymphatic vessels, CD31 indicates blood vessels, α-SMA indicates smooth muscle. Abbreviations: LMM: lamina muscularis mucosae. The yellow arrow represents lymphatic vessels. Scale bar:100um

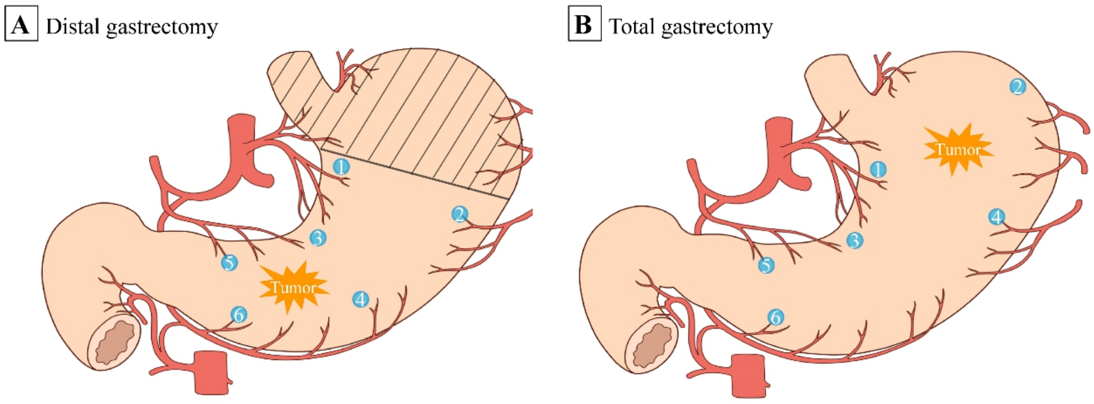

① between the 1st and the 2nd gastric wall branches of the left gastric artery
② the 1st gastric wall branch of the left gastroepiploic artery
③ the gastric angular notch
④ the midpoint of two injection points (② and ⑥) in the greater curvature
⑤ the 1st gastric wall branch of the right gastric artery
⑥ the 1st gastric wall branch of the right gastroepiploic artery

① between the 1st and the 2nd gastric wall branches of the left gastric artery
② the junction area between fundus and body in the greater curvature
③ the gastric angular notch
④ the 1st gastric wall branch of the left gastroepiploic artery
⑤ the 1st gastric wall branch of the right gastric artery
⑥ the 1st gastric wall branch of the right gastroepiploic artery

eFigure 1. Illustration of Six Points for Subserosal Injection of ICG
A. Distal gastrectomy. B. Total gastrectomy.

The SURG-TLX

There are six rating scales which are meant for evaluating your experience during the laparoscopic surgery procedure. Please evaluate the procedure by marking 'X' on each of the six scales at the point which best fits your experience. The Surg-TLX subscale item were rated on a 20-point scale (0 = low, 20 = high). Please read the descriptions carefully.

Mental Demands

How mentally fatiguing was the procedure?

0 Very Low — Very Hig 20

Physical Demands

How physically fatiguing was the procedure?

0 Very Low — Very High 20

Temporal Demands

How hurried or rushed was the pace of the procedure?

0 Very Low — Very High 20

Task Complexity

How complex was the procedure?

0 Not Very Complex — Very Complex 20

Situational Stress

How anxious did you feel while performing the procedure?

0 Not Very Anxious — Very Anxious 20

Distractions

How distracting was the operating environment?

0 Not Very — Very 20

eFigure 2. The Surg-TLX Questionnaire

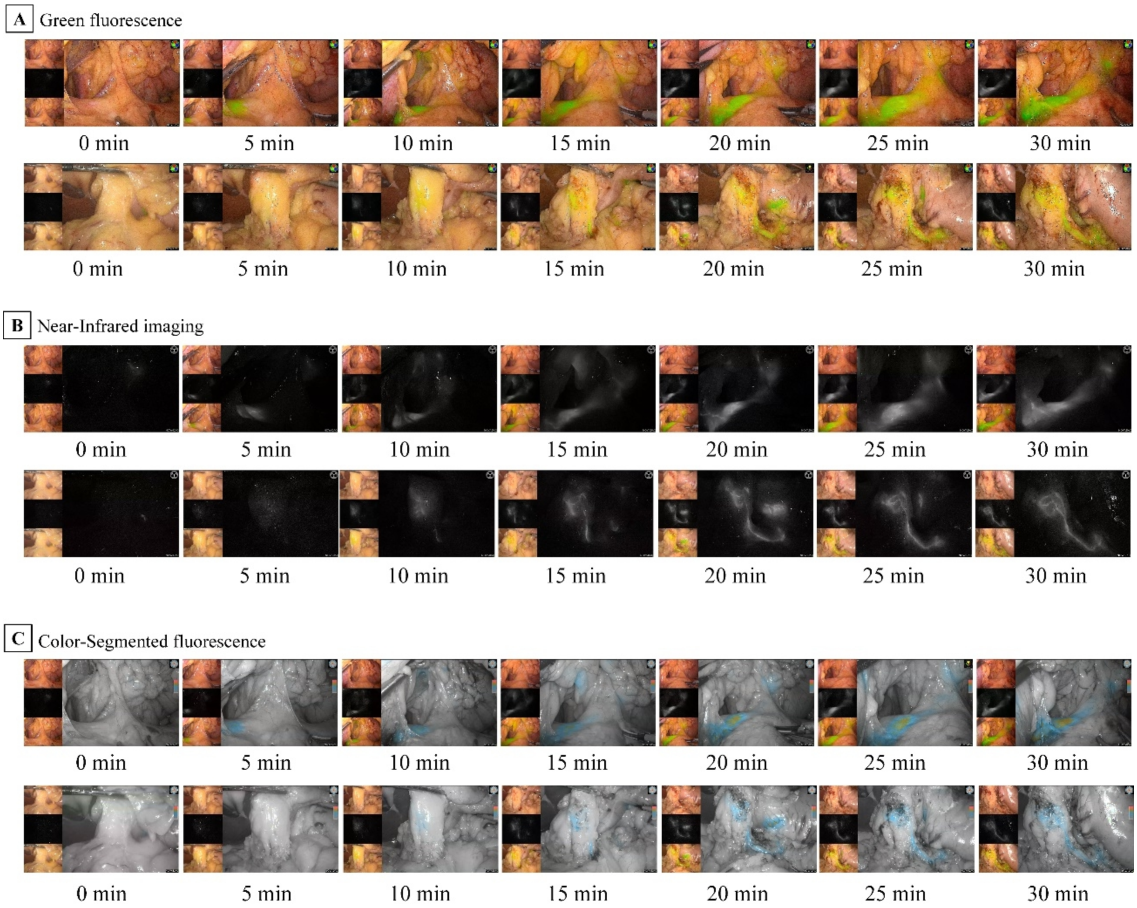

eFigure 3. View of Lymph Nodes Under Near-Infrared Fluorescent Imaging After Subserosal Injection of ICG

A. Green fluorescence. B. Near-Infrared imaging. C. Color-Segmented fluorescence.

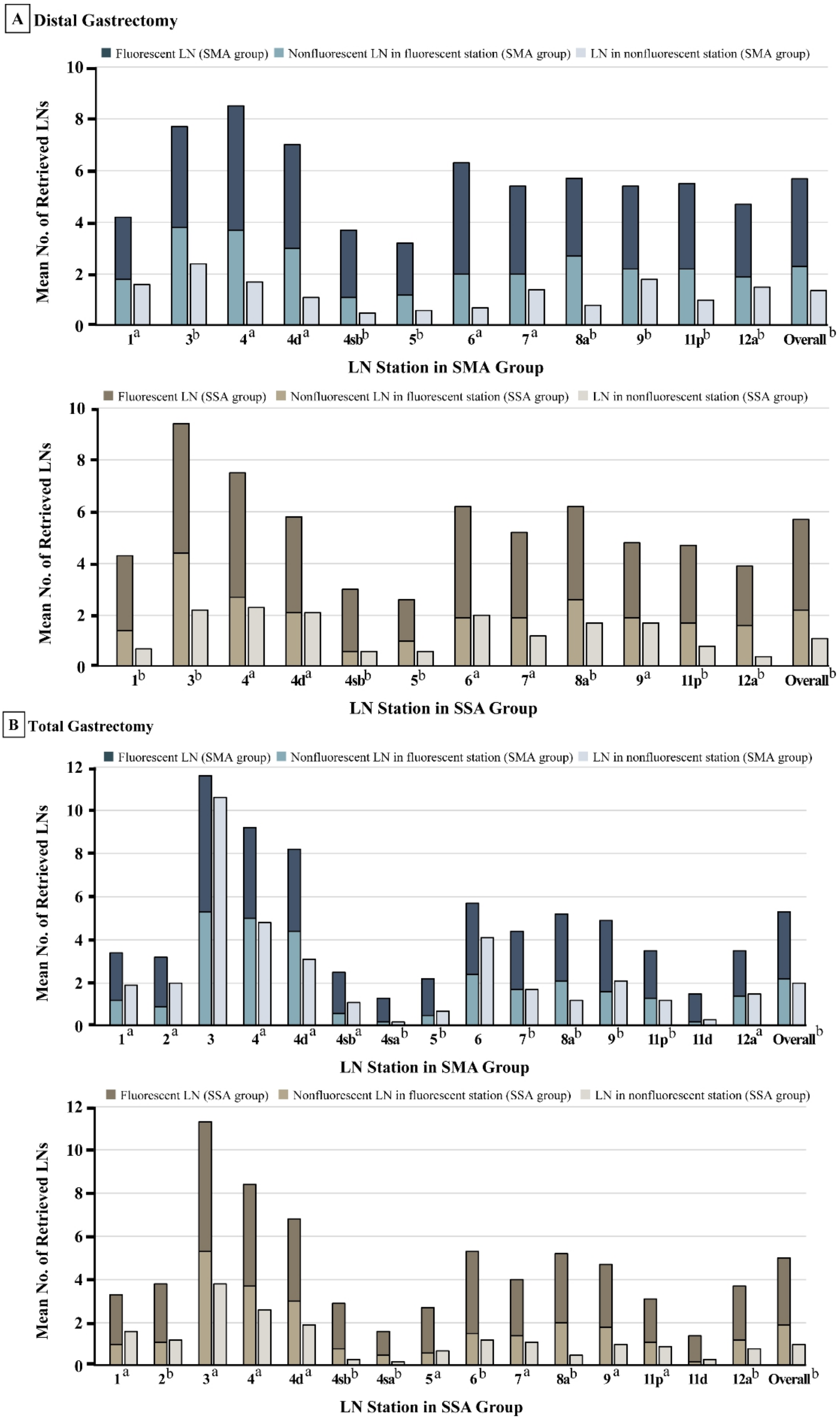

eFigure 4. Mean Number of Fluorescent Lymph Nodes, Nonfluorescent Lymph Nodes in Fluorescent Stations, and Lymph Nodes in Nonfluorescent Stations According to the Lymph Node Station in the SMA and SSA Groups

[a]*P*<.05, [b]*P*<.001.

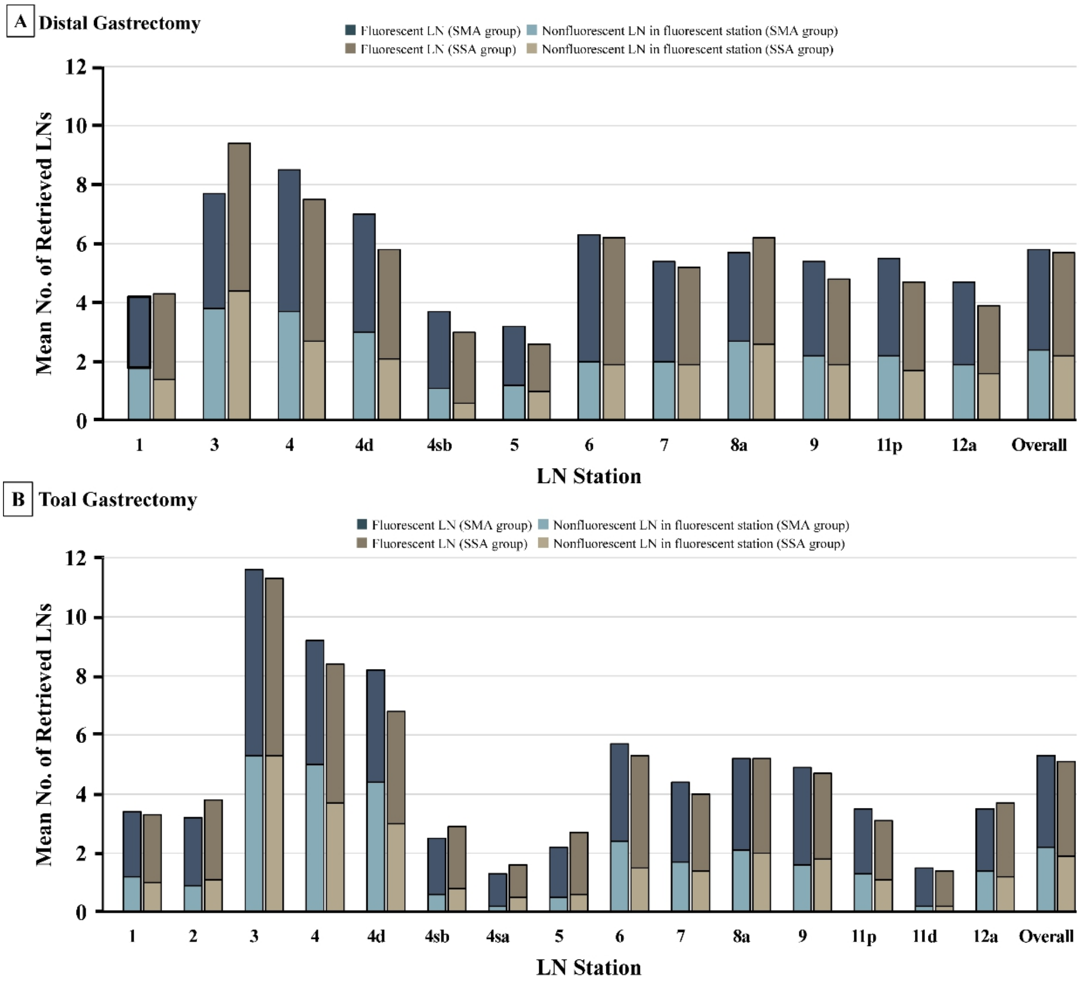

eFigure 5. Mean Number of Fluorescent Lymph Nodes and Nonfluorescent Lymph Nodes in Fluorescent Stations According to the Lymph Node Station in the SMA and SSA Groups

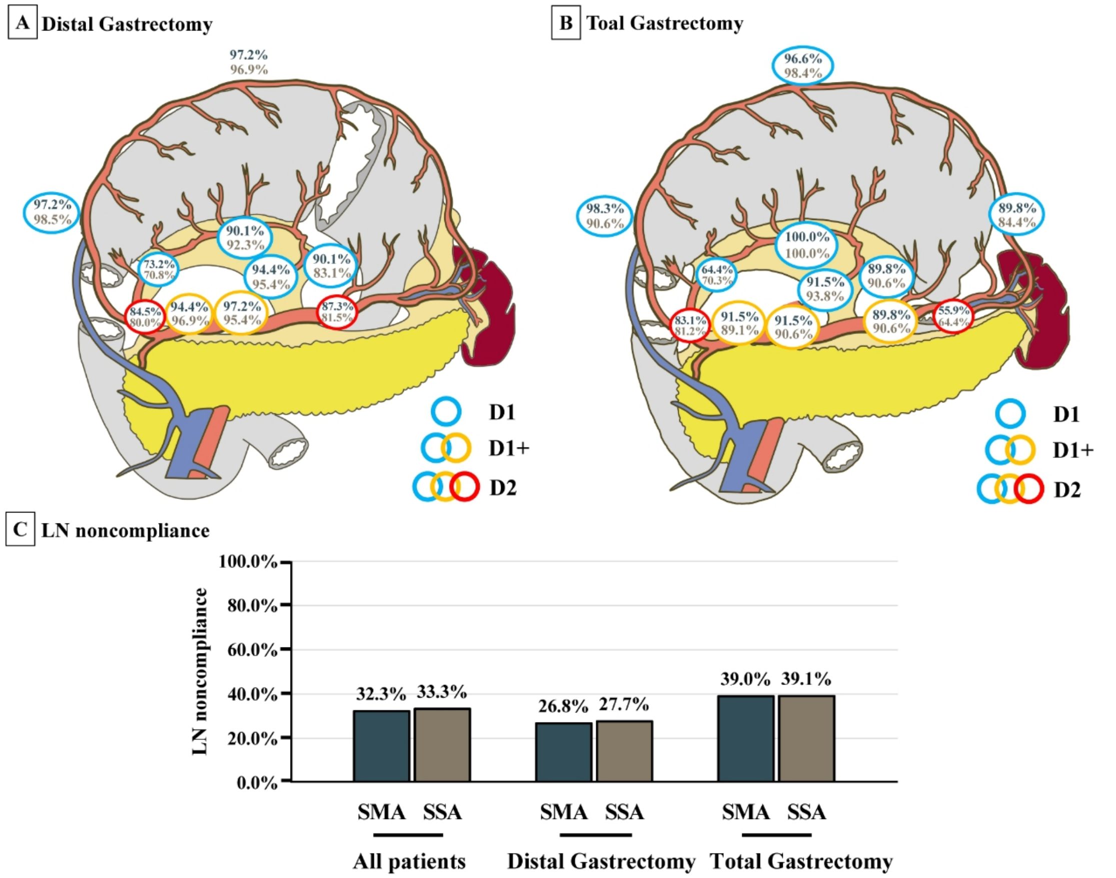

eFigure 6. Lymph Node Dissection Rate at Each Station in the SMA and SSA Groups During Distal and Total Gastrectomy with D2 Lymph Node Dissection

A. Distal gastrectomy with D2 lymph node dissection. B. Total gastrectomy with D2 lymph node dissection. C. Noncompliance with lymph node dissection in the SMA group is shown in dark blue text and that in the SSA group is shown in gray text. SMA, submucosa approach; SSA, subserosa approach.

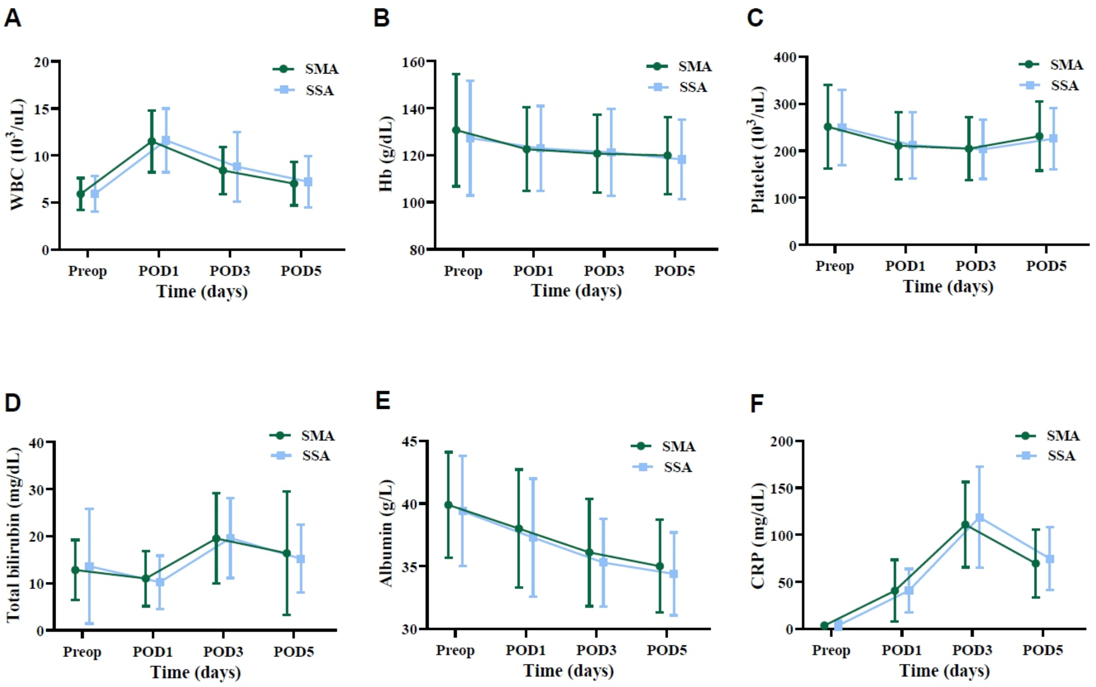

eFigure 7. Laboratory Findings Estimated Before Surgery and on Postoperative Days 1, 3, and 5.

(A) White blood cell (WBC) count, (B) hemoglobin (Hb) level, (C) platelet count, (D) total bilirubin level, (E) albumin level, and (F) C-reactive protein (CRP).

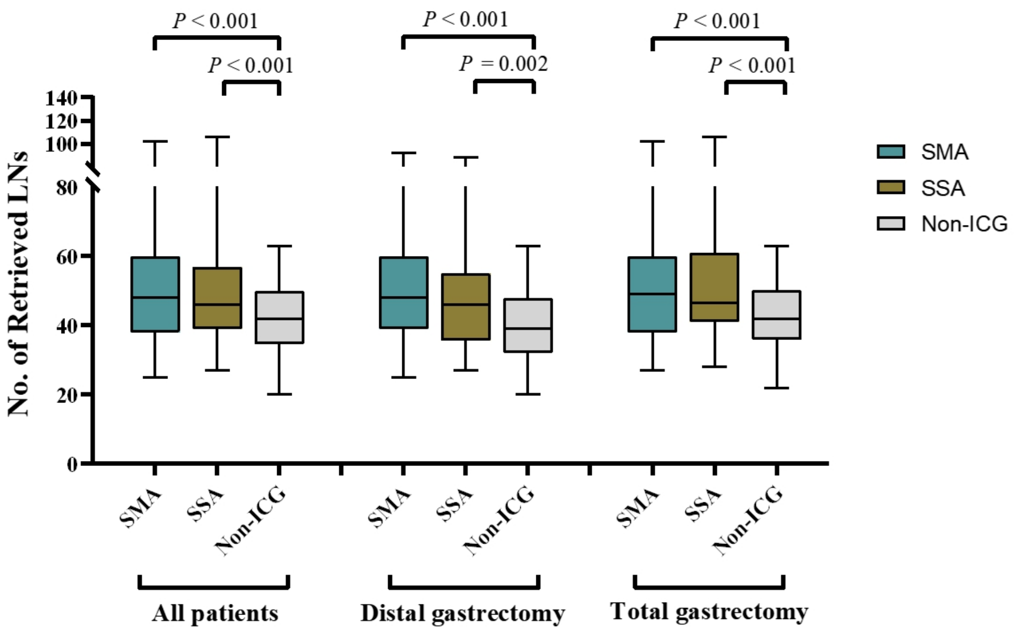

eFigure 8. Total Number of Retrieved Lymph Nodes in the SMA, SSA and Non-ICG Groups

SMA, submucosa approach; SSA, subserosa approach.

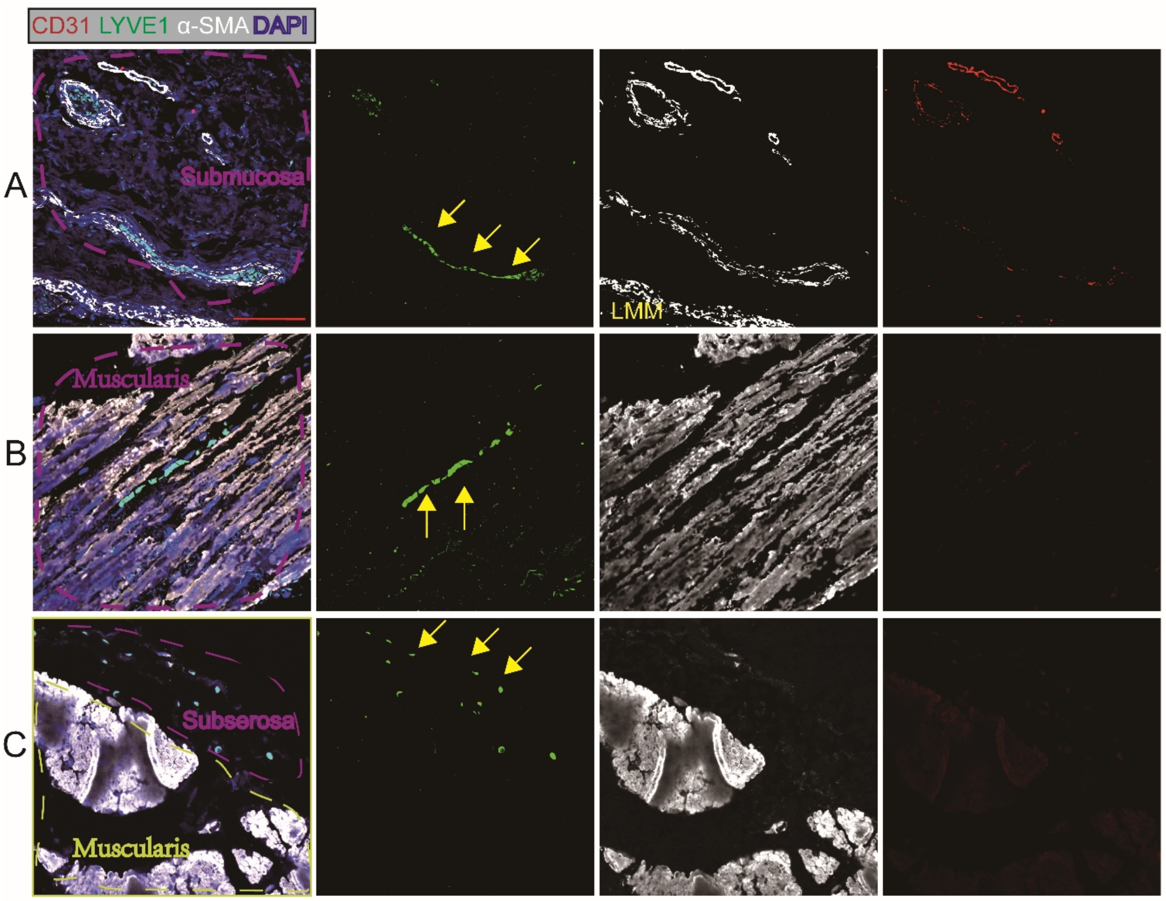

eFigure 9. The Lymphatic System in the Gastric Wall. A, submucous plexus; B, lymphatic vessels among the muscular fibers; C, subserous plexus. LYVE1 indicates lymphatic vessels, CD31 indicates blood vessels, α-SMA indicates smooth muscle. Abbreviations: LMM: lamina muscularis mucosae. The yellow arrow represents lymphatic vessels. Scale bar: 100um
