# Supplementary material for: Comparison of submucosal and subserosal approaches toward optimized indocyanine green tracer-guided laparoscopic lymphadenectomy for patients with gastric cancer (FUGES-019): a randomized controlled trial
Source: BMC Med. 2021 Oct 27;19:276. doi: 10.1186/s12916-021-02125-y (PMC8549272; doi:10.1186/s12916-021-02125-y)
Supplement: Supplementary file 4 — Additional file 4. Detailed method. [file 12916_2021_2125_MOESM4_ESM.docx]

**Supplemental Content 3.** **Detailed method**

**Immunohistochemistry**

Tissue specimens were fixed with formalin, embedded in paraffin, and serially sectioned at 10 μm. The sections were deparaffinized with dimethyl benzene and rehydrated with a graded series of ethanol. Antigen retrieval was performed with sodium citrate buffer, and endogenous peroxidase activity was blocked by hydrogen peroxide. The sections were then blocked with 10% goat serum and incubated with primary antibody at 4°C overnight. The primary antibodies used were mouse anti-D2-40 (MAB-0567, MXB Biotechnologies). Subsequently, the sections were incubated with secondary antibodies at room temperature. The signal was developed with a diaminobenzidine (DAB) solution, and the slides were counterstained with hematoxylin. Finally, the slides were scanned and analyzed.

**Immunofluorescence**

Immunofluorescence was performed according to standard protocols. In summary, seven-micrometer sections from the paraffin blocks and tissue slides were deparaffinated and rehydrated, followed by antigen retrieval via heating to 121 °C in a pressure cooker in standard 10 mM citric acid pH6 buffer, a commercial citrate pH 6.1 buffer (S1699, DAKO). Primary antibodies used were rabbit anti-LYVE1 (1:100, ReliaTech GmbH, 102-PA50S), mouse anti-α-SMA (1:200, Sigma, A2547), rat anti-CD31 (1:100, BD Bioscience, 550274). Secondary antibodies used were anti-rabbit, anti-rat or -mouse Alexa 488, cy3 or cy5 IgG (1:200, Invitrogen) for immunofluorescence. Confocal images were obtained with a Zeiss LSM T-PMT confocal laser-scanning microscope (Carl Zeiss).
